# Developing nucleoside tailoring strategies against SARS-CoV-2 via ribonuclease targeting chimera

**DOI:** 10.1126/sciadv.adl4393

**Published:** 2024-04-10

**Authors:** Yuanqin Min, Wei Xiong, Wei Shen, Xingyu Liu, Qianqian Qi, Yuanyuan Zhang, Ruochen Fan, Fang Fu, Heng Xue, Hang Yang, Xiulian Sun, Yunjia Ning, Tian Tian, Xiang Zhou

**Affiliations:** ^1^ Wuhan Institute of Virology; Hubei Jiangxia Laboratory; Center for Biosafety Mega-Science, Chinese Academy of Sciences, Wuhan 430200, Hubei, China.; ^2^ Key Laboratory of Biomedical Polymers of Ministry of Education, College of Chemistry and Molecular Sciences, Hubei Province Key Laboratory of Allergy and Immunology, Wuhan University, Wuhan 430072, Hubei, China.

## Abstract

In response to the urgent need for potent severe acute respiratory syndrome coronavirus 2 (SARS-CoV-2) therapeutics, this study introduces an innovative nucleoside tailoring strategy leveraging ribonuclease targeting chimeras. By seamlessly integrating ribonuclease L recruiters into nucleosides, we address RNA recognition challenges and effectively inhibit severe acute respiratory syndrome coronavirus 2 replication in human cells. Notably, nucleosides tailored at the ribose 2′-position outperform those modified at the nucleobase. Our in vivo validation using hamster models further bolsters the promise of this nucleoside tailoring approach, positioning it as a valuable asset in the development of innovative antiviral drugs.

## Teaser

Nucleoside tailoring strategies with ribonuclease targeting show promise in inhibiting SARS-CoV-2 replication.

## INTRODUCTION

There is an urgent need for effective drugs as short-term weapons to combat severe acute respiratory syndrome coronavirus 2 (SARS-CoV-2) (*1*–*3*). Scientists have embarked on an unprecedented race to explore various approaches to accelerating the development of anti–SARS-CoV-2 drugs (*4*–*6*). Targeting viral proteins has emerged as an important approach in developing therapeutics against this virus (*7*–*9*). As an RNA virus, SARS-CoV-2 presents a unique set of challenges in drug discovery (*10*). The high mutation rate in RNA viruses enables swift adaptation to environmental challenges (*11*). This mutagenicity not only complicates the drug development process but also underscores the necessity for a multipronged approach to ensuring long-term efficacy (*12*). Among diverse methods being investigated, nucleoside analogs have emerged as a promising class of antivirals (*13*–*15*). They function primarily by disrupting viral replication, a critical step in the virus’ life cycle, thus potentially halting its spread and mitigating disease severity (*16*, *17*).

**Fig. 1.**
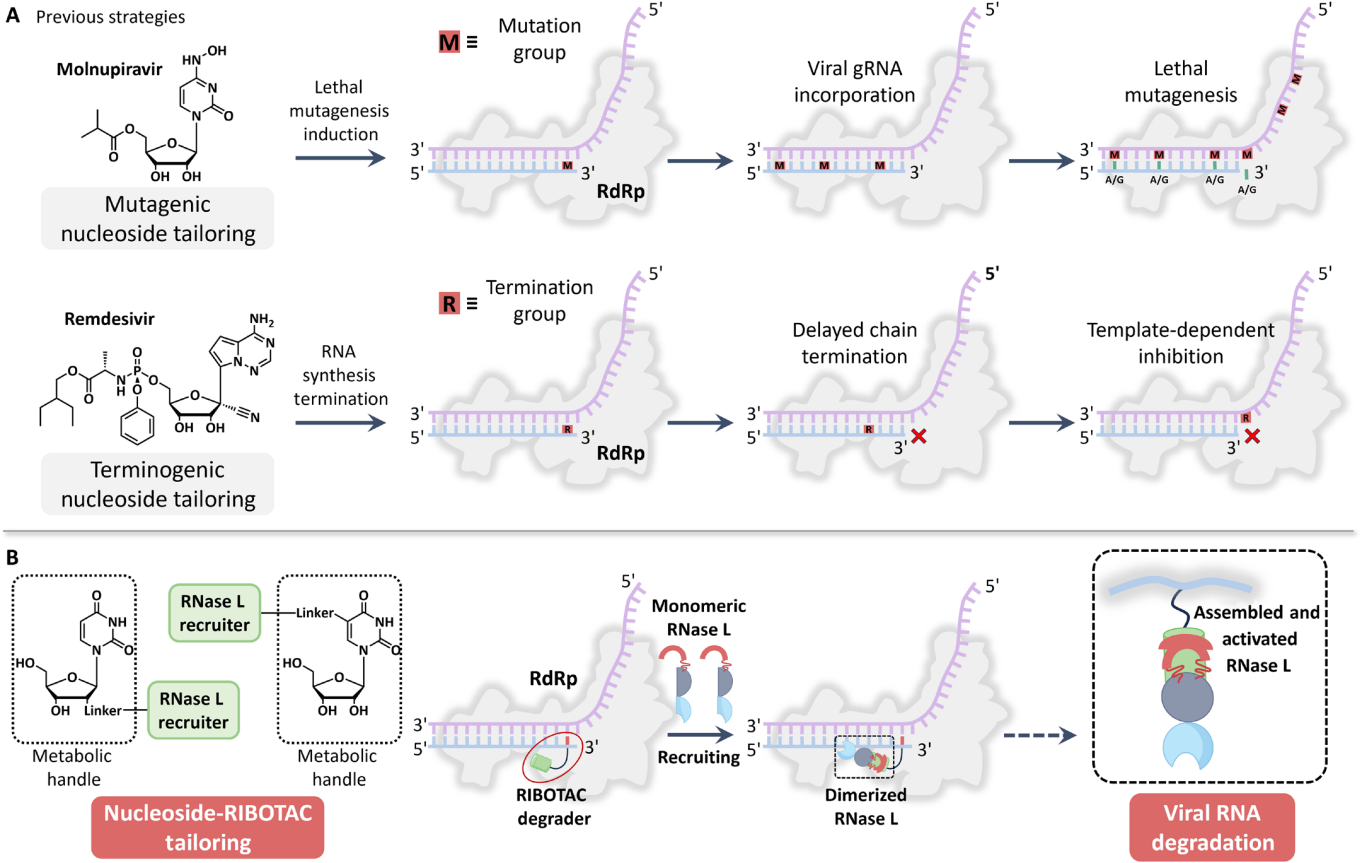
Advancing nucleoside tailoring strategies against SARS-CoV-2 with RIBOTAC. (**A**) Known nucleoside tailoring strategies against SARS-CoV-2. One strategy involves nucleoside tailoring with mutagenic properties, inducing lethal mutagenesis in the virus, while the other strategy uses nucleoside tailoring with RNA synthesis termination properties, inducing termination of viral RNA replication. gRNA, guide RNA. (**B**) RIBOTAC-enabled nucleoside tailoring against SARS-CoV-2.

Nucleoside analogs, mimicking natural nucleosides, can be incorporated into the RNA chain and exhibiting varied effects based on their design and mechanism (*18*–*20*). The antiviral capabilities of these analogs are intimately linked with nucleoside tailoring strategies. Presently, there are two principal strategies to disrupt the RNA replication process of SARS-CoV-2 using nucleoside analogs (Fig. 1A) (*21*). The first strategy involves nucleoside tailoring with mutagenic properties, closely associated with lethal mutagenesis (*22*, *23*), exemplified by Molnupiravir. Here, the goal is to increase the mutation rate of the virus to a threshold where it accumulates fatal errors and can no longer replicate effectively (*24*, *25*). The second involves nucleoside tailoring with RNA synthesis termination properties, correlated with RNA chain termination (*26*, *27*), and is represented by remdesivir. This method aims to halt the virus’ replication prematurely. Currently, both strategies have achieved a certain level of success, particularly molnupiravir, which has been approved as an oral drug for treating SARS-CoV-2 infections (*23*). However, because of the mechanism of action of molnupiravir (*28*), this form of treatment might potentially trigger the evolution of viral lineages carrying numerous mutations (*29*). As scientists tread this path, striking a balance between efficacy and safety remains paramount (*30*). Therefore, the exploration of innovative nucleoside tailoring strategies will be beneficial for antiviral drug discovery.

With unique physicochemical and pharmacological characteristics, RNA-targeted modalities have emerged as a distinct class of modalities and are under active development (*31*–*35*). On the basis of the strategy of nucleic acid hydrolysis targeting chimera, chimeric antisense oligonucleotides were developed for efficient inhibition of the envelope or spike RNA of SARS-CoV-2 in vitro (*36*). Moreover, the degradation of RNA using small-molecule RNA binders has been attempted in the form of ribonuclease (RNase) targeting chimeras (RIBOTACs) (*37*, *38*). These are designed entities that essentially serve as a bridge, recruiting cellular RNases to specific RNA targets, thereby leading to the degradation of these RNAs (*39*, *40*). However, the basis of RIBOTAC is to identify specific RNA secondary structures through small molecules (*41*, *42*). Achieving specificity and high binding affinity for these RNA secondary structures is challenging (*43*–*45*). Therefore, our objective is to incorporate the RNA degradation properties of RIBOTACs, bypassing the need for small-molecule recognition of RNA secondary structures, to develop or improve nucleoside tailoring strategies for inhibiting SARS-CoV-2.

Our findings have provided a proof of principle for the development of nucleoside tailoring strategies, aiming to inhibit SARS-CoV-2 by inducing viral degradation during replication. The nucleosides are modified in their sugar or nucleobase moieties to craft advanced nucleoside analogs with powerful biological activity (Fig. 1B). We report the short-step synthesis of tailored nucleosides with RNase recruiting groups. Through various experimental methodologies, we have demonstrated that our nucleoside analogs, developed via nucleoside-RIBOTAC tailoring strategies, effectively inhibit SARS-CoV-2 replication within human cells. When modifying the ribose 2′-position with RIBOTAC structural units, the antiviral activity of the corresponding nucleoside analog is superior to those modified at the nucleobase. Mutating the RIBOTAC structural units leads to a noticeable decline in the antiviral activity of the corresponding nucleoside analogs, highlighting the crucial role of RIBOTAC units. Furthermore, our in vivo experiments in hamsters verified that nucleoside-RIBOTAC tailoring strategies effectively inhibit SARS-CoV-2 replication and activity. This affirms the substantial potential of our nucleoside tailoring strategies in antiviral drug development, especially during emerging infections when there is an absence of virus-specific antivirals.

## RESULTS

### Chemical synthesis of different tailored nucleosides

**Fig. 2.**
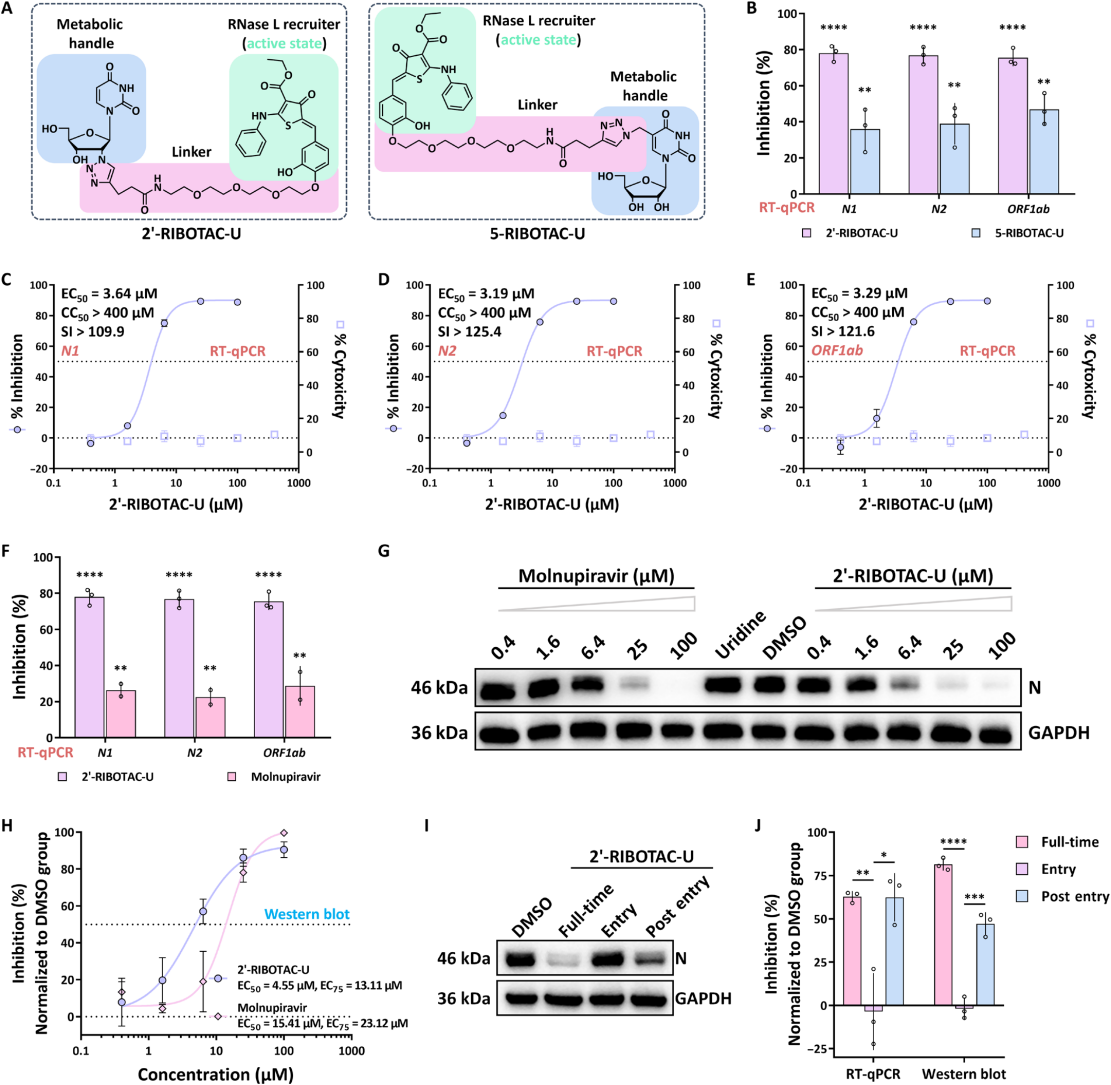
Effective inhibition of SARS-CoV-2 in human cells by nucleoside-RIBOTAC tailoring. (**A**) Structure of tailored nucleosides. (**B**) Viral inhibition rate (normalized to DMSO group) from RT-qPCR analysis of RNA samples from SARS-CoV-2–infected cells treated with 6.4 μM of each nucleoside. (**C** to **E**) Concentration-dependent inhibition of intracellular SARS-CoV-2 replication by 2′-RIBOTAC-U in RT-qPCR experiments on different gene segments, with (C), (D), and (E) corresponding to *N1*, *N2*, and *ORF1ab*, respectively. (**F** and **G**) Benchmarking our strategy against known approaches, with comparisons conducted using RT-qPCR (F) and Western blot (G); in (F), both compounds are at a concentration of 6.4 μM. (**H**) Quantitative analysis of Western blot results from (G). (**I**) Time-of-compound-addition assay assessing 2′-RIBOTAC-U’s inhibition of SARS-CoV-2 in cells, with a consistent concentration of 12.5 μM throughout the assay. The assay was conducted across distinct conditions: DMSO (DMSO for 1 hour, 1-hour virus with DMSO, and 24-hour DMSO culture), full-time (compound for 1 hour, 1-hour virus with compound, and 24-hour compound culture), entry (compound for 1 hour, 1-hour virus with compound, and 24-hour culture with DMSO), and postentry (1 hour with DMSO, 1-hour virus with DMSO, and 24-hour compound culture). (**J**) Quantitative analysis of Western blot results from (I) and the inhibition rate derived from RT-qPCR analysis (N2), with all tests using a 12.5 μM concentration of 2′-RIBOTAC-U. The *P* value of the results are indicated (*P < 0.05, **P < 0.01, ***P < 0.001, and ****P < 0.0001).

On the basis of the devised nucleoside tailoring strategies, the initial step involves the chemical synthesis of nucleoside analogs. Chemical modifications on ribonucleosides typically use two distinct strategies: One involves modifying the ribose moiety (*46*, *47*), and the other involves altering the nucleobase (*48*, *49*). Tailoring the RNase recruiting module on the nucleoside was performed in accordance with these two distinct positions. Consequently, this led to the synthesis of two distinct nucleoside analogs, each associated with either ribose or nucleobase modification (Fig. 2A). The synthetic routes for compounds 2′-RIBOTAC-U and 5-RIBOTAC-U are illustrated in the Supplementary Materials (*50*). Compound **1** (3,4-dihydroxybenzaldehyde) undergoes nucleophilic substitution with bromo-PEG-NHBoc in the presence of potassium carbonate as the base, resulting in the formation of compound **3**. Subsequently, compound **3** reacts via a Michael addition with a thiophene derivative (fig. S7) to yield compound **5**. Compound **5** is then subjected to *t*-butyloxycarbonyl deprotection using trifluoroacetic acid, leading to compound **6**. Through further amide condensation with pent-4-ynoic acid–*N*-hydroxysuccinimide activated ester (fig. S7), compound **6** transforms into alkyne-functionalized RIBOTAC compound **9**. Last, compound **9** was reacted separately with 2′-azido-uridine (fig. S7) or 5′-azidomethyl-uridine (fig. S9) via copper-catalyzed click chemistry, resulting in the formation of 2′-RIBOTAC-U and 5′-RIBOTAC-U, respectively. The structures of all intermediates and target compounds were thoroughly characterized.

### Effective inhibition of SARS-CoV-2 in human cells by nucleoside-RIBOTAC tailoring

After synthesizing uridine modified separately at the ribose and nucleobase of the RIBOTAC module, we aim to evaluate their antiviral effectiveness. In the assessment of compound efficacy against SARS-CoV-2, the human hepatocellular carcinoma cell line, Huh7-ACE2, offers a dependable model (*51*). This cell line serves as an effective simulation of the infection process of viruses such as SARS-CoV-2 under laboratory conditions. Hence, we use this cell line to evaluate the impact of candidate compounds on viral infection. For our study, Huh7-ACE2 cells were pretreated with the indicated compounds for 1 hour, followed by SARS-CoV-2 infection at a multiplicity of infection (MOI) of 0.05 for 1 hour in the presence of each compound. At 1 day after infection, viral RNA levels in the cytoplasm and supernatant were quantified using the reverse transcription quantitative polymerase chain reaction (RT-qPCR) technique. Three distinct viral gene segments, with two being derived from the same nucleocapsid (N) gene—namely, *N1* and *N2*—and the third originating from the *ORF1ab* gene, were selected for evaluation. The experimental results reveal that both tested nucleosides were capable of suppressing the replication of SARS-CoV-2 in cells (Fig. 2B). The quantitative results for all three segments were consistently aligned. When exposed to different tailored nucleosides at the same concentration, the molecule with 2′-modification in the RIBOTAC (2′-RIBOTAC-U) exhibited a substantially stronger inhibitory effect on SARS-CoV-2 replication compared to the molecule with nucleobase modification in the RIBOTAC (5-RIBOTAC-U).

On the basis of the initial results, we selected the more potent 2′-RIBOTAC-U for a comprehensive quantitative assessment of its inhibitory activity against SARS-CoV-2. Using cells infected with the SARS-CoV-2 virus and their culture supernatants as test samples, we determined the compound’s ability to inhibit the viral load within the cells and in the culture supernatant. Data from our RT-qPCR experiments revealed that 2′-RIBOTAC-U can concentration-dependently suppress the SARS-CoV-2 viral load both intracellularly and in the culture supernatant. The half-maximal effective concentration (EC_50_) values for 2′-RIBOTAC-U’s inhibition of intracellular viral activity generally fell within the low-micromolar magnitude range, specifically between 3.19 and 3.64 μM (Fig. 2, C to E). Notably, the EC_50_ for its suppression of the virus in the culture supernatant closely mirrored that of its intracellular inhibition (fig. S1, A to C). Furthermore, to assess potential cytotoxicity, we conducted a Cell Counting Kit-8 assay. The results highlighted that, even at a concentration of 400 μM, which is substantially higher than the compound’s EC_50_ against the virus, no cytotoxic effects on the cells were observed. This underscores 2′-RIBOTAC-U’s impressive selectivity index (SI) [50% cytotoxicity concentration (CC_50_)/EC_50_], indicating its potential as a promising therapeutic candidate.

We aim to compare the current nucleoside tailoring strategy with the established nucleoside tailoring approach. Therefore, we conducted a benchmarking study between 2′-RIBOTAC-U and the known lethal mutagen molnupiravir. The results of RT-qPCR on selected gene segments indicated that 2′-RIBOTAC-U exhibits substantially superior anti–SARS-CoV-2 activity at the cellular level (Fig. 2F and fig. S1, D to F). To further assess the antiviral activities of 2′-RIBOTAC-U and molnupiravir, we also performed Western blot analysis. In our experiments, Huh7-ACE2 cells were pretreated with varying doses of the specified compounds for 1 hour, followed by SARS-CoV-2 infection (0.05 MOI) in the presence of the compounds. At 24 hours after infection, cells were collected, and the viral N proteins in the obtained lysates were analyzed using antibodies. The results demonstrated that both compounds substantially reduced viral protein expression (Fig. 2, G and H). Notably, the negative control, uridine, had no impact on virus replication even at a high concentration of 100 μM. Furthermore, we observed that 2′-RIBOTAC-U exhibited superior anti–SARS-CoV-2 effects at lower concentrations such as 1.6, 6.4, and 25 μM. Further analysis involving fitting the inhibitory effects of the compounds at different concentrations revealed that 2′-RIBOTAC-U has a lower EC_50_ value, indicating its more effective inhibition of SARS-CoV-2 activity. It is worth mentioning that at a high concentration of 100 μM, molnupiravir demonstrated more profound inhibition of viral protein expression. This could be attributed to the larger molecular volume of 2′-RIBOTAC-U compared to molnupiravir, potentially affecting its cellular uptake ability.

To elucidate the timing of compound inhibition on the virus and determine which phase of the SARS-CoV-2 lifecycle is affected by 2′-RIBOTAC-U, we conducted time of compound addition assays. We treated cells with the compound during various stages of the virus’ life cycle. Under the full-time condition, cells were treated with 2′-RIBOTAC-U for 1 hour and then infected with SARS-CoV-2. An hour later, they were cultured in a compound-containing medium for 24 hours. Under the entry condition, after the same initial treatment and infection, cells were cultured in a compound-free medium for 24 hours. For the postentry condition, after 1 hour in fresh medium and SARS-CoV-2 infection, cells were cultured in a compound-containing medium for 24 hours before sampling. Following the collection of samples under these different conditions, we conducted Western blot analysis to examine the expression levels of SARS-CoV-2 proteins using the indicated antibodies. Alternatively, RT-qPCR was used to analyze viral RNA. Both Western blot and RT-qPCR results were consistent and in line with our previous findings (Fig. 2, I and J, and fig. S1G).

Under the full-time treatment, 2′-RIBOTAC-U effectively inhibited viral infection, leading to substantially lower viral protein levels compared to the dimethyl sulfoxide (DMSO) group (Fig. 2, I and J, right part) and considerable suppression of viral RNA (left part in Fig. 2J and fig. S1G). In contrast, when 2′-RIBOTAC-U was only added during the initial phase of viral infection and removed after 2 hours (entry condition), the expression of viral N protein was comparable to the DMSO-treated group. Correspondingly, there was no substantial suppression of intracellular viral RNA observed, suggesting that 2′-RIBOTAC-U did not effectively inhibit the virus during its entry into the cells. When evaluating the scenario where the compound was introduced postviral entry (postentry condition), as expected, introducing 2′-RIBOTAC-U after the virus had entered the cells still proved effective in inhibiting the viral infection. The suppression rate of intracellular viral RNA was comparable to the full-time treatment (left part in Fig. 2J and fig. S1G), and while viral protein levels were slightly higher than in the full-time treatment group, they were still notably reduced compared to the DMSO group (Fig. 2, I and J, right part). All these findings indicate that 2′-RIBOTAC-U exerts its inhibitory action after the invasion of SARS-CoV-2 into the cells. This aligns with our expectations of 2′-RIBOTAC-U acting as a nucleoside analog, inhibiting the viral replication process, and thereby demonstrating its antiviral mechanism of action.

### Functional validation of the RIBOTAC module in tailored nucleosides

**Fig. 3.**
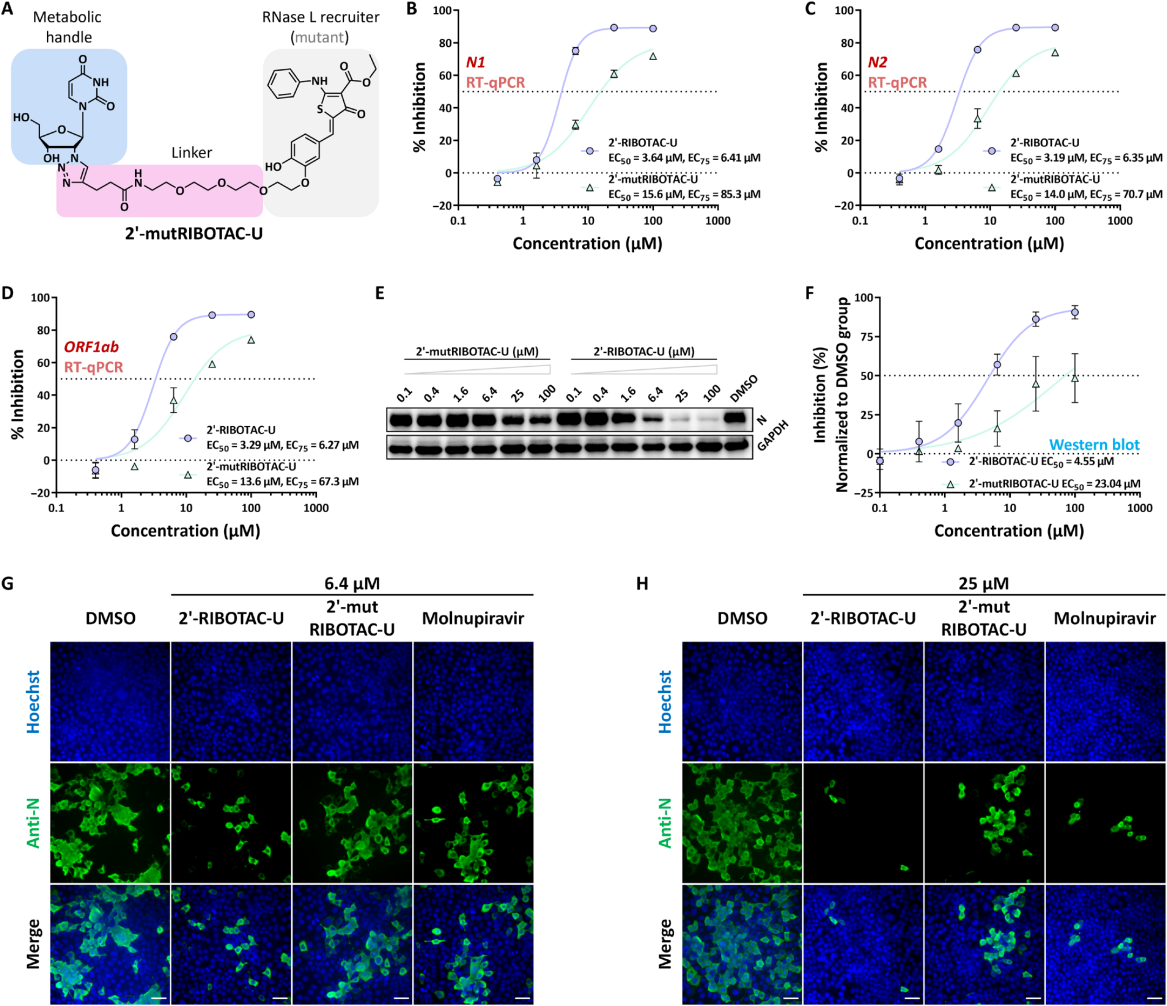
Functional validation of the RIBOTAC module in tailored nucleosides. (**A**) Chemical structure of a nucleoside with the mutated RNase L recruiter. (**B** to **D**) Comparisons of the inhibitory activity against intracellular SARS-CoV-2 by different nucleosides with wild-type or mutated RNase L recruiters, based on RT-qPCR results targeting various gene segments: (B) *N1*, (C) *N2*, and (D) *ORF1ab*. The solid purple circles represent the inhibition rates calculated after treatment with different concentrations of 2′-RIBOTAC-U (normalized to the DMSO group). The solid green triangles represent the inhibition rates calculated after treatment with different concentrations of 2′-mutRIBOTAC-U (normalized to the DMSO group). (**E**) Western blot analyses comparing the inhibitory effects on intracellular SARS-CoV-2 by various tailored nucleosides. (**F**) Quantitative analysis of Western blot results from (E). (**G** and **H**) Immunofluorescence findings comparing the inhibitory activity of diverse tailored nucleosides and molnupiravir against intracellular SARS-CoV-2. (G) corresponds to results at 6.4 μM concentrations of the compounds, while (H) presents data at 25 μM concentrations. Scale bars, 50 μm.

The tailored nucleosides could potentially exert an impact on viral replication through one or more nonmutually exclusive mechanisms. For instance, the incorporation of substituent groups in tailored nucleosides might introduce steric hindrance, thereby potentially obstructing viral RNA replication and even leading to RNA chain termination. In accordance with our design, the RNase L recruiter itself should have steric hindrance, while serving as the cleavage site (*34*). It ought to exhibit a greater hindrance effect on inhibiting SARS-CoV-2 activity than its inherent substituent groups alone. To validate this notion, we introduced structural mutations to the RIBOTAC module. This mutated RNase L recruiter represents an isomer of the wild-type RIBOTAC module, known for its diminished ability to recruit RNase L due to the altered heterocycle’s capacity (*50*). We successfully synthesized 2′-mutRIBOTAC-U (Fig. 3A) carrying the mutated RNase L recruiter and conducted a comparative study of its anti–SARS-CoV-2 activity alongside 2′-RIBOTAC-U.

We initiated the study by conducting RT-qPCR experiments, focusing on various gene segments. The findings that emerged from this study underscore the crucial role of the RNase L recruiter’s structural configuration. It became evident that alterations in this module led to a substantial attenuation in the potency of tailored nucleosides to suppress SARS-CoV-2 viral load both intracellularly (Fig. 3, B to D) and in the culture supernatant (figs. S1, A to C, and S2, A to C). The quantitative results from the analysis of three distinct gene segments consistently indicate substantial increases in the EC_50_ values for SARS-CoV-2 inhibition with 2′-mutRIBOTAC-U carrying the mutated RNase L recruiter. Notably, a more substantial difference becomes evident when comparing the 75% maximal effective concentration (EC_75_) values from RT-qPCR for 2′-mutRIBOTAC-U and molnupiravir, with 2′-mutRIBOTAC-U demonstrating substantially reduced efficacy (fig. S3, A to C). The reduced activity against SARS-CoV-2 can be attributed to the RIBOTAC module’s mode of action, highlighting the crucial role of its integrity in modulating the inhibitory potential of tailored nucleosides.

We also conducted Western blot assays to compare compound activity at the viral protein level and confirm the functionality of the RIBOTAC module. The results were largely consistent with the RT-qPCR findings, collectively indicating that structural alterations in the RIBOTAC module substantially diminish the inhibitory activity against SARS-CoV-2 (Fig. 3, E and F). In the case of 2′-mutRIBOTAC-U carrying the mutated RNase L recruiter, noticeable viral protein expression persisted even at a concentration of 25 μM. Furthermore, upon elevating the compound concentration to 100 μM, there was no substantial increase in its antiviral effectiveness compared to the 25 μM concentration. In conducting a quantitative analysis of the inhibitory effects of various compounds on viral protein expression across different concentrations, a marked disparity is observed in the EC_75_ values from Western blot for 2′-mutRIBOTAC-U compared to molnupiravir, with the former showing substantially lower effectiveness—over 10-fold in Western blot analyses (fig. S3D). Both Western blot and RT-qPCR results collectively underscore the pivotal role of the RIBOTAC module in mediating the inhibitory activity against SARS-CoV-2 exhibited by the compounds.

We further conducted immunofluorescence experiments, where SARS-CoV-2–infected cells treated with different compounds were subjected to fixation at room temperature using 4% paraformaldehyde. After washing, cell permeabilization, and blocking, detection was carried out using monoclonal antibodies against the SARS-CoV-2 N protein and fluorescently labeled secondary antibodies. The results indicate that 2′-RIBOTAC-U molecules equipped with functional RIBOTAC modules, at a concentration of 6.4 μM, substantially suppressed viral protein expression (Fig. 3G). This suppression was evidenced by a substantial decrease in detected fluorescence levels. Conversely, when the structure of the RIBOTAC module was altered, the corresponding molecule’s ability to inhibit viral protein expression was notably attenuated. As the compound concentration was raised to 25 μM, cells treated with 2′-RIBOTAC-U displayed almost negligible expression of viral proteins (Fig. 3H). In contrast, cells treated with 2′-mutRIBOTAC-U, carrying a mutated RIBOTAC module, still exhibited considerable viral protein expression. Our findings also indicate that the inhibitory activity of 2′-RIBOTAC-U molecules with functional RIBOTAC modules against SARS-CoV-2 surpasses that of molnupiravir. Collectively, these results underscore the pivotal role of the RIBOTAC module in our developed nucleoside tailoring strategies for combating the virus.

### Nucleoside-RIBOTAC tailoring against SARS-CoV-2 in hamsters

**Fig. 4.**
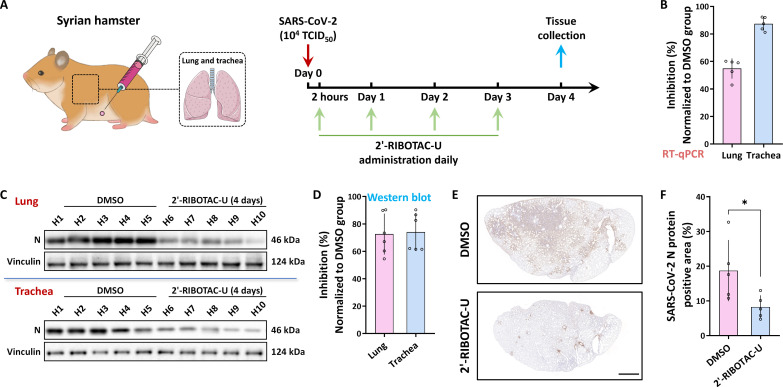
Nucleoside-RIBOTAC tailoring against SARS-CoV-2 in hamsters. (**A**) Schematic of hamster drug administration (intraperitoneal injection) and the postviral infection dosing regimen [infection dose of 10^4^ median tissue culture infectious dose (TCID_50_), medication starts 2 hours after infection, once daily at 200 mg/kg); tissues, proteins, and RNA samples are extracted from relevant sites for analysis 4 days later. (**B**) Analysis and calculated inhibition rate of the RNA extracted from the lung and trachea sections through RT-qPCR (normalized to the DMSO group). (**C**) Results of Western blot analysis on the protein extracted from the lung and trachea sections, focusing on vinculin and N protein expression levels. (**D**) Quantitative analysis of results from (C), after normalization to the DMSO group, showcasing the inhibition rate of compounds against N protein expression. (**E**) Representative results of immunohistochemical staining of lung tissue sections (DMSO group and 2′-RIBOTAC-U group). Scale bar, 2000 μm. (**F**) Outcome from analyzing the stained areas in (E) and (fig. S4) through the ImageJ software (statistical analysis was performed using unpaired Student’s *t* test; error bars denote ±SD across technical replicates, *n* = 5, *P < 0.05).

The results above confirm the notable anti–SARS-CoV-2 effects of nucleoside-RIBOTAC tailoring strategies at the cellular level. As animal experiments can provide information closer to real biological conditions, we aim to further evaluate the antiviral effects of nucleoside-RIBOTAC tailoring strategies using Syrian hamster infection model (*52*). This transition from cellular-level experiments to preclinical studies is crucial. For this experiment, 6-week-old female Syrian hamsters were selected and obtained from a specific pathogen–free environment. They were acclimated to the laboratory conditions for 2 days. The hamsters were randomly divided into two groups, with *n* = 5 in each group. SARS-CoV-2 infection was administered through intranasal inoculation. Following infection, the treatment group of hamsters received daily intraperitoneal injections of the antiviral compound 2′-RIBOTAC-U at a dose of 200 mg/kg, while the control group received an equivalent volume of physiological saline solution with DMSO. The administration was performed once daily (Fig. 4A). On the fourth day after infection, hamsters from both groups were euthanized, and tissues such as lungs and trachea were collected for subsequent experiments.

During the observation period, experimental hamsters administered 2′-RIBOTAC-U exhibited good tolerance, showing no discernible adverse effects. We first extracted total RNA from tissues such as the lungs and trachea. The RT-qPCR analysis targeting the N gene indicated that the replication of SARS-CoV-2 viral RNA was substantially inhibited following treatment with 2′-RIBOTAC-U (Fig. 4B). Furthermore, through Western blot analysis, we assessed the expression levels of the viral N protein in the lungs and trachea. Consistent with the RT-qPCR results, the treatment with 2′-RIBOTAC-U markedly suppressed the expression levels of the SARS-CoV-2 N protein in these tissues (Fig. 4, C and D).

Our macroscopic findings from RT-qPCR and Western blot shed light on viral activity and molecular-level insights. To delve deeper into the tailored nucleoside’s impact on viral activity at the tissue level, we undertook additional investigations. Immunohistochemical staining serves primarily to localize and quantify specific protein expression in tissue sections (*53*). Using a monoclonal antibody specifically targeting the SARS-CoV-2 N protein, we performed in situ examinations of viral infections and distribution within hamster lung tissue sections. The intensity of this staining—ranging from blue (negative) to pale yellow (weakly positive) to brown-yellow (moderately positive) and deep brown (strongly positive)—correlates with the antigen content and its distribution density. As depicted in Fig. 4E and fig. S4, lung sections from the DMSO group presented an abundance of N protein–positive staining regions. Compared to the DMSO-treated samples, the 2′-RIBOTAC-U treatment markedly reduced the SARS-CoV-2 N protein–positive staining areas in the lung tissue sections. Quantitative analysis using the ImageJ image processing software revealed that the average grayscale value (indicative of staining intensity) and the percentage of positive staining area were substantially reduced in the 2′-RIBOTAC-U treated group when compared to the DMSO control group, as seen in Fig. 4F. In conjunction with the preceding Western blot and quantitative PCR results, it is evident that 2′-RIBOTAC-U treatment reduces the SARS-CoV-2 infection in pulmonary tissues in vivo, thus offering potent anti–SARS-CoV-2 effects at the tissue level in animals.

To gain deeper insights into the lung tissue morphology after infection, we carried out hematoxylin and eosin (H&E) staining on lung tissue sections (*54*). This widely accepted histological technique is instrumental in discerning the pathological changes occurring within lung tissues of infected hamsters. Viral invasion in tissues invariably triggers a plethora of tissue reactions, such as inflammation, necrosis, or cell infiltration. These pathological alterations bear a direct relationship with the outcomes of H&E staining. Our aspiration was to decipher, via H&E staining, whether the tailored nucleosides exhibit therapeutic potential against tissue damage instigated by the virus.

**Fig. 5.**
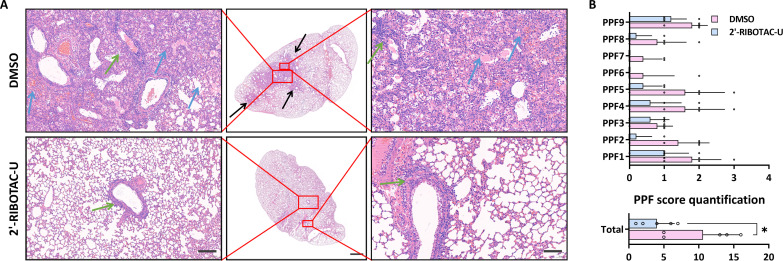
SARS-CoV-2 inhibition in hamsters via nucleoside-RIBOTAC tailoring. (**A**) Representative histological analysis of lung tissue sections. For the control group: notable large patchy consolidation areas in lung tissue (black arrows), substantial inflammatory cell exudation in alveolar cavities, hemorrhage and edema (blue arrows), presence of inflammatory cells and edematous fluid in the bronchial lumen, and sleeve-like inflammatory cell infiltration around interstitial blood vessels and bronchi (green arrows), thickened alveolar walls, and congestion. For the 2′-RIBOTAC-U group: sleeve-like inflammatory cell infiltration around interstitial vessels and bronchi (green arrows). Scale bars, 100 μm (first column), 1000 μm (second column), and 50 μm (third column). (**B**) Grading of pathological sections from (A), (figs. S5 and S6), based on all biological repeats. PPF represents “pulmonary pathological feature,” evaluating the pathological manifestation in the lungs from an imaging perspective: PPF1 refers to the degree of congestion, blood stasis, and thickening of the alveolar walls; PPF2 evaluates from the perspective of the extent of pulmonary edema; PPF3 is an assessment from the standpoint of hemorrhage in the alveolar space; PPF4 gauges the degree of inflammatory cell infiltration in the alveolar cavity; PPF5 assesses the extent of lung consolidation; PPF6 indicates the degree of hemorrhage in the bronchial lumen; PPF7 examines from the perspective of edema in the bronchial lumen; PPF8 is an assessment of the degree of inflammatory cell exudation within the bronchial lumen; PPF9 evaluates on the basis of the degree of inflammatory cell infiltration around the interstitium (surrounding blood vessels and bronchi). Lesions were ranked on a scale of 1 to 4, indicating mild, moderate, moderate to severe, and severe, respectively, while 0 indicates no detectable lesion. The resultant pathological scores are plotted (statistical analysis was performed using unpaired Student’s *t* test; error bars denote ±SD across technical replicates, *n* = 5, *P < 0.05).

After 4-day SARS-CoV-2 exposure, the lung tissue of the hamsters in the DMSO group (untreated control) manifested pronounced inflammatory responses and severe lung lesions. A representative sample is showcased in the first row of Fig. 5A, revealing vast areas of consolidated lung tissue (black arrow), extensive inflammatory cell infiltration in the alveolar spaces, hemorrhagic edema (blue arrow), and inflammatory cell with edema in bronchial lumens. Around the interstitial blood vessels and bronchi, there is a sleeve-like infiltration of inflammatory cells (green arrow), with evident thickening and congestion of the alveolar walls. A comprehensive analysis of the entire DMSO control group demonstrated that four of five hamsters exhibited varying degrees of lung consolidation and corresponding symptoms, such as alveolar inflammation and edema (Fig. 5, A and B, and fig. S5). Every hamster from this group manifested alveolar wall congestion and thickening, with interstitial inflammation. A subset (1 to 3) exhibited pathological findings such as hemorrhage, edema, and inflammation in the bronchial lumens.

In stark contrast, the pathology severity in the 2′-RIBOTAC-U treated group was markedly less intense compared to the DMSO control. As displayed in the second row of Fig. 5A, 2′-RIBOTAC-U treatment considerably ameliorated the inflammatory responses within lung tissues, with a conspicuous reduction in inflammation caused by the virus. An overarching analysis of the entire 2′-RIBOTAC-U treatment group revealed that only two of five mice showed slight lung consolidation, with the majority retaining relatively intact alveolar structures (Fig. 5A and fig. S6). The overall inflammation, pulmonary edema, and conditions of alveolar wall congestion/thickening were substantially improved, with no evidence of hemorrhage or edema in the bronchial lumens. The pathological scoring for the 2′-RIBOTAC-U treatment group can be found in Fig. 5B. Both the H&E-stained lung tissue sections and the detailed pathological analysis (fig. S6) corroborate the potent anti–SARS-CoV-2 effects of 2′-RIBOTAC-U at the tissue level in animals.

## DISCUSSION

The efficacy of the nucleoside-RIBOTAC tailoring strategy may stem from the RNA-dependent RNA polymerase (RdRp) of SARS-CoV-2 being more receptive to modified nucleosides than host cell RNA polymerases (*20*, *55*). Known nucleoside tailoring techniques with mutagenic attributes have achieved some success (*56*). However, they elevate the chances of engendering emergent SARS-CoV-2 mutations and carry potential reproductive toxicities. These concerns highlight the necessity for more refined nucleoside tailoring strategies, especially for populations sensitive to the side effects of these nucleoside analogs. Our nucleoside analogs are designed to mimic natural nucleosides closely. This close mimicry increases the likelihood that even as the virus mutates, these nucleoside analogs will continue to be incorporated into the viral RNA, maintaining their efficacy. Once incorporated into the viral RNA, these modified nucleosides can attract RNase L, triggering the degradation of the virus’ genomic RNA and disrupting subsequent replication or transcription. From a design perspective, this mechanism minimizes the risk of inducing viral mutations and presents a potentially lower risk profile to the host.

We show that in contrast to pyrimidine ribonucleosides modified at the nucleobase, the 2′-modified counterparts exhibit greater activity in inhibiting intracellular SARS-CoV-2 replication. In the field of nucleoside modifications and their metabolic incorporation, there have been studies involving the 2′-modification of ribonucleosides (*46*, *47*). These modified nucleosides have been taken up by cells and incorporated into cellular or viral RNA through metabolic pathways. Recent work demonstrated the capability of SARS-CoV-2 RdRp, to incorporate 2’-F,Me-UTP, the active form of sofosbuvir (*20*). We reasoned that the modifications at the 2′-position will encourage incorporation of the activated triphosphate analogue by SARS-CoV-2 polymerases but not host cell polymerases, thus reducing any side effects.

Traditional RIBOTACs rely heavily on recognizing specific secondary structures present on the target RNA, a feature that necessitates the use of small-molecule binders (*57*). Achieving high specificity and binding affinity with these binders has often been challenging, limiting the effectiveness of conventional RIBOTAC approaches (*58*). The nucleoside-RIBOTAC tailoring strategy elegantly circumvents this issue by harnessing the dual benefits of nucleoside analogs and RIBOTAC mechanisms. The tailored nucleosides used in this strategy are relatively small in size, suggesting a probable ease of cellular uptake. This characteristic augments their potential as efficient antiviral agents. Furthermore, a deeper delve into their chemical structure reveals their classification as small-molecule drugs, with modifications that are chemically stable. This stability plays a pivotal role in their longevity and efficiency when deployed intracellularly or even systemically. Beyond their present design, the nucleoside-RIBOTAC tailoring strategy appears ripe for further optimization. Enhancements could emerge from refining the RNase L recruiting unit, fine-tuning the linker regions, or exploring other structural modifications (*35*). The integration of these improvements, along with the inherent benefits of this strategy, makes the nucleoside-RIBOTAC tailoring a promising candidate for antiviral therapy against SARS-CoV-2 and possibly other RNA viruses in the future.

While the nucleoside-RIBOTAC tailoring strategy holds promise, it is crucial to acknowledge its current challenges and areas that need further refinement. One of the primary concerns is the anti–SARS-CoV-2 activity of the tailored nucleosides. As of now, their potency requires amplification, particularly in therapeutic scenarios targeting active SARS-CoV-2 infections as opposed to preventative measures. In addition to concerns about efficacy, there is the perennial challenge of safety. The intricacies of cellular mechanisms mean that introducing tailored agents, such as our nucleosides, can lead to unforeseen interactions and consequences within the cell. However, a key aspect of our approach is the modular design of the RIBOTAC components integrated into nucleoside analogs. In instances where off-target effects are identified, this design enables us to swiftly modify the molecular structure of the RIBOTACs to reduce these unintended interactions. Our understanding of the precise mechanism of action for our tailored nucleosides remains preliminary. A more granular approach is needed. For instance, synthesizing 5′-mono-, -di-, and -triphosphates of our tailored nucleosides and then studying their enzymatic properties with SARS-CoV-2 RdRp will provide deeper insights. The cellular transformation of our tailored nucleosides into their corresponding 5′-mono-, -di-, and -triphosphates is another dimension that demands exploration. The activity of both viral and cellular kinases in this transformation process will be pivotal to understand, ensuring the efficient conversion and subsequent antiviral activity. Ongoing research is focused on understanding these interactions and developing nucleoside analogs with minimal interference in host cell metabolism.

This study demonstrates that nucleoside tailoring strategies using RIBOTAC structural units effectively inhibit SARS-CoV-2 replication in human cells and hamsters, offering substantial potential for antiviral drug development.

## MATERIALS AND METHODS

### Ethics statements

Ten female hamsters (6 weeks old) were randomly assigned to two groups in this study. Viral infections were performed according to the standard operating procedures of the biosafety level 3 (BSL-3) facility. All processes of the animal experiment were in line with recommendations for the care and use of laboratory animals and the Institutional Review Board of the Wuhan Institute of Virology, Chinese Academy of Sciences (ethics number: WIVA17202005).

### Virology experiments setting

All virological experiments with SARS-CoV-2 were conducted in a BSL-3 at the National Biosafety Laboratory, Wuhan, within the Chinese Academy of Sciences.

### Compound antiviral assay

In acquisition of Huh7-ACE2 cells, the pLVX-IRES-PURO-hACE2 expression plasmid encoding human ACE2 was constructed. This plasmid, along with lentiviral packaging vectors psPAX2 and pMD2.G, was cotransfected into human embryonic kidney–293T cells. After 48 hours, the supernatant was harvested to obtain lentiviral particles. The packaged lentivirus was then used to transduce Huh7 cells. Selection was performed with puromycin (2 μg/ml) for 3 to 4 days. Surviving cells were then harvested and subjected to a limiting dilution technique to obtain single cells. Western blotting (WB) was later used to identify cell lines stably expressing ACE2 (*59*).

### Compound antiviral experiment

Huh7-ACE2 cells were initially seeded at ~1.5 × 10^5^ in a 24-well plate, and, on the subsequent day, ~2.5 × 10^5^ Huh7-ACE2 cells were used. This experimentation began with a 20 mM stock solution of the compound in DMSO. Ideally, following a fourfold dilution pattern, the third concentration should have been 6.25 μM. However, for ease of recognition and consistency in the dilution series, this was adjusted to 6.4 μM. Following the 6.4 μM concentration, the fourfold dilution pattern was continued, resulting in subsequent concentrations of 1.6 μM, 0.4 μM, and the smallest concentration being 0.1 μM. This compound solution, along with DMSO, was incorporated into Dulbecco’s modified Eagle’s medium (DMEM) complete medium at a 1:200 ratio and mixed thoroughly. The old medium from the Huh7-ACE2 cells was then discarded, and, beginning with the lowest concentration, 400 μl of medium containing varied concentrations of the compound was added, with three replicate wells set up for each concentration (with the exception of molnupiravir, for which two replicate wells were prepared). One hour after the compound was added, the cells were infected with a viral diluent at an MOI of roughly 0.05 (100 μl per well), with the plate gently shaken every 15 min for an hour, totaling four times. The supernatant was then discarded and substituted with 500 μl of medium carrying either DMSO or the specific compound concentrations, after which cells were incubated for an additional 24 hours. When collecting cell supernatant samples, they were inactivated by heating at 65°C for half an hour. Cells could either be lysed using TRIzol or with 1× SDS loading buffer and then heated at 100°C for 10 min. Once inactivated, all sample tubes were placed in a disinfectant-filled container. Following surface disinfection, the container could be safely transported out of the BSL-3 laboratory.

### Time-of-addition experiment with 2′-RIBOTAC-U

For the experiments, 2′-RIBOTAC-U was maintained at a final concentration of 12.5 μM. Under the DMSO control condition, cells underwent treatment with DMSO for 1 hour before SARS-CoV-2 infection. One hour later, the medium was swapped out and replaced with DMSO-containing medium, with cells cultured for an additional 24 hours before collecting samples. For the full-time condition, cells were treated with 2′-RIBOTAC-U for 1 hour before the SARS-CoV-2 infection. An hour after the infection, the medium was refreshed, and cells were then cultivated in a 2′-RIBOTAC-U–containing medium for another 24 hours before sample collection. In the entry procedure, after a 1-hour treatment with 2′-RIBOTAC-U, cells were infected with SARS-CoV-2. One hour after infection, the medium, devoid of the compound, was replaced, and cells were left to culture for another 24 hours leading up to sampling. Last, under the postentry condition, cells were prepped with fresh medium, excluding the compound, for 1 hour before SARS-CoV-2 infection. An hour after this infection, the medium was replenished with one containing 2′-RIBOTAC-U, and the cells underwent a 24-hour incubation phase ahead of sampling.

### Hamster-based antiviral activity testing of 2′-RIBOTAC-U

Ten 6-week-old female hamsters (~100 g per hamster) were purchased from Beijing Vetone Leihua and divided into two groups of five. The hamsters were acclimated in A3 Individual Ventilated Cages (IVC) cages for 1 to 2 days. After anesthetizing the hamsters, they were exposed to the virus through nasal drops, with each hamster receiving a dose of 1 × 10^4^ median tissue culture infectious dose (TCID_50_)/50 μl of SARS-CoV-2. Approximately 2 hours after exposure, the hamsters were again anesthetized and intraperitoneally injected with the 2′-RIBOTAC-U compound (0.05 mg/μl dissolved in 50% DMSO, 200 mg/kg, ~400 μl per hamster). This treatment was repeated daily. On the fourth day after exposure, the hamsters were euthanized to obtain lung and tracheal tissues, which were placed in preweighed tubes containing either DMEM for tissue grinding or 4% paraformaldehyde fixative. The tubes were reweighed. The fixed samples were kept in the dark at room temperature in an animal BSL-3 laboratory for more than 10 days. These samples were later used for immunohistochemistry and pathological analyses. The tissue grinding tubes were placed in prechilled biosafe tissue grinding rotors, surface sterilized, and loaded into a tissue grinder to obtain tissue homogenates. After inactivation, they were used for subsequent RNA extraction and WB detection (refer to subsequent sections for RNA extraction and WB sample preparation).

### RNA preparation and reverse transcription

Cell or tissue homogenate samples were mixed proportionally with either TRIzol or TRIzol LS (Life Technologies). After complete lysis, the mixture was transferred to centrifuge tubes and removed from the BSL-3 laboratory. RNA was extracted as per standard procedures, and its concentration was measured using a NanoDrop instrument. One micrograms of RNA was reverse-transcribed using the PrimeScript RT Reagent Kit with genomic DNA (gDNA) Eraser (TAKARA) to obtain cDNA.

For the cell supernatant samples, they were heat-inactivated at 65°C for 30 min. Upon removal from the BSL-3 laboratory, RNA was extracted using the Micro Viral RNA Extraction Kit (TAKARA, 9766). From this, 5 μl samples were reverse-transcribed using the PrimeScript RT Reagent Kit with gDNA Eraser (TAKARA) to produce cDNA.

### qPCR analysis

Dye-based quantification was performed using the SYBR Premix Ex Taq II (Tli RNaseH Plus) kit (TAKARA). The template cDNA used was 1 to 2 μl per 20 μl of reaction mix, with both forward and reverse primers having a final concentration of 0.4 μM (refer to table S2 for details). The amplification protocol was set as follows: 95°C for 30 s, followed by 40 cycles of 95°C for 5 s, 62°C for 15 s, and 72°C for 45 s, with signal collection during the 72°C elongation phase. Target mRNA levels were relatively quantified using the comparative Cycle Threshold (CT) method (2^−ΔΔCT^ method): ΔΔCT = treatment group (Ct target gene − Ct GAPDH) − control group (Ct target gene − Ct GAPDH). Here, GAPDH (glyceraldehyde-3-phosphate dehydrogenase) was used as the internal reference gene. For determining the viral RNA copy number in the cells, standard curves were established using the plasmids containing the indicated target gene cDNA (ranging from 10^2^ to 10^8^ copies/μl) for absolute quantification.

### Immunofluorescence

After treating with different compounds, the infected samples in the 24-well plates had their supernatants discarded. The entire plate was then fixed at room temperature for 30 min in containers filled with 4% paraformaldehyde. Cells were washed three times with phosphate-buffered saline (PBS) and permeabilized with 0.5% Triton X-100 for 10 min. Afterward, cells were blocked using 5% bovine serum albumin (BSA) at 37°C for an hour. Primary anti–SARS-CoV-2 N monoclonal antibody (ABclonal, #A20142), diluted 1:500 in PBS, was added and incubated at 4°C overnight. After washing three times with PBS (10 min each time), a secondary fluorescein isothiocyanate–conjugated sheep anti-mouse antibody, diluted 1:500 in PBS, was added and incubated at 37°C for 1 hour. Following three more PBS washes (10 min each), cell nuclei were stained with Hoechst for 5 to 10 min. After two PBS washes, images were captured under a fluorescence microscope (OLYMPUS IX53) and later analyzed using the ImageJ software.

### Western blot analysis

After rinsing cell samples with PBS, they were lysed with 1× SDS sample buffer and thoroughly mixed. This mixture was then transferred to centrifuge tubes. Tracheal homogenate samples, which had a ratio of 0.1 g homogenized in 1 ml of DMEM, had 200 μl combined with 5× SDS sample buffer. Lung tissue homogenate samples, due to their viscosity and having a similar initial ratio, were diluted 5 to 10 times with DMEM and mixed with 5× SDS sample buffer. These Western blot samples underwent heat treatment at 100°C for 10 min in a BSL-3 laboratory. Before SDS–polyacrylamide gel electrophoresis (PAGE), these samples were reheated to 100°C for 5 min and centrifuged, and the resultant supernatant underwent SDS-PAGE, membrane transfer, and a 5% BSA block. For the primary antibody incubation, target antibodies in tris-buffered saline with 0.1% Tween 20 (TBST) were introduced to the membranes, left overnight at 4°C, and subsequently washed three times with TBST. The membranes were then exposed to secondary antibodies at 37°C for an hour and washed three times with TBST. Last, enhanced chemiluminescence substrate was applied to the membranes, and protein expression was visualized using a chemiluminescence detection system.

### Lung tissue section preparation

Hamster lung tissues were fixed in 4% paraformaldehyde for 10 days and, due to BSL-3 laboratory protocols, remained in the laboratory for that duration. After fixation, tissues were put into a dehydration container inside a fume hood. The tissues were then dehydrated using a gradient alcohol regimen in a DIPATH Donatello dehydration machine, progressing from 75% alcohol to paraffin III. These tissues, now soaked in paraffin, were embedded using a Wuhan Junjie Electronics JB-P5 machine: Melted paraffin was poured into molds, tissues from the dehydration container were added, labels were attached, and samples were cooled at −20°C. After solidification, paraffin blocks were trimmed and then sectioned to 4 μm in thickness using a Leica RM2016 microtome. These sections were floated on a 40°C water bath in a KD-P slide warmer to smooth out, then collected on glass slides, and oven-dried at 60°C. For deparaffinization, sections underwent treatment with eco-friendly solutions from Seville Biotechnology, followed by absolute ethanol washes and a final rinse in distilled water. After this process, sections were primed for H&E staining or immunohistochemistry.

### Lung tissue section immunohistochemistry

Sections underwent antigen retrieval using citric acid antigen retrieval solution from Seville Biotechnology with a pH of 6.0, microwaved at varying heats, ensuring minimal buffer evaporation. After cooling naturally, the slides were rinsed three times in PBS. Notably, the retrieval solution and conditions depend on the tissue type. To block endogenous peroxidase, sections were exposed to a 3% hydrogen peroxide solution in darkness and room temperature for 25 min, followed by PBS washes. A 3% BSA solution was subsequently applied for serum blocking. Excess solution was removed before incubating the sections with a primary antibody from ABclonal, diluted in PBS, and left in a humidified chamber at 4°C overnight. After PBS washes, the sections were treated with a horseradish peroxidase–labeled secondary antibody from Servicebio. The 3,3′-diaminobenzidine substrate was used for staining, which was closely monitored for a brown-yellow hue indicating positive staining and stopped with tap water. The nuclei were counterstained with hematoxylin, differentiated, and blued using specific solutions, followed by rinses. Sections underwent a specific dehydration series, culminating in an air-drying phase and neutral resin mounting. Slides were then scanned with the PANNORAMIC system and observed with the CaseViewer2.4 software. Last, the blue hematoxylin–stained nuclei contrasted with the brown-yellow positive 3,3′-diaminobenzidine staining for result interpretation.

### Lung tissue section H&E staining

Slides underwent staining with Harris hematoxylin for a duration between 3 to 8 min and were rinsed with tap water. A brief differentiation was achieved using 1% hydrochloric acid in alcohol, and after another rinse, they were blued using 0.6% ammonia water, concluding with a comprehensive rinse under running water. For cytoplasmic staining, the sections were immersed in eosin stain for a span of 1 to 3 min. Following this, they were sequentially dehydrated via a regimen involving 75 and 85% alcohol, two rounds of absolute ethanol, butanol, and xylene. Once dehydrated, they were allowed a brief air-drying period before being mounted with neutral resin. To analyze the results, the entirety of the slides was scanned using the PANNORAMIC DESK/MIDI/250/1000 system and examined via the CaseViewer2.4 software. During the assessment, key pathological changes such as inflammation, congestion, stasis, hemorrhage, edema, and degeneration were identified. For clearer understanding, representative pathological regions were captured in images, with arrows marking specific lesions.

## Supplementary Materials

### This PDF file includes:

Figs. S1 to S9 General Chemical Procedures Tables S1 and S2 General Biological Procedures Data S1 and S2 Legend for data S3 References
