# Supplementary material for: Developing nucleoside tailoring strategies against SARS-CoV-2 via ribonuclease targeting chimera
Source: Sci Adv. 2024 Apr 10;10(15):eadl4393. doi: 10.1126/sciadv.adl4393 (PMC11006213; doi:10.1126/sciadv.adl4393)
Supplement: Supplementary file 1 — Figs. S1 to S9 General Chemical Procedures Tables S1 and S2 General Biological Procedures Data S1 and S2 Legend for data S3 References [file sciadv.adl4393_sm.pdf]

Science Advances
AAAS

Supplementary Materials for

Developing nucleoside tailoring strategies against SARS-CoV-2 via ribonuclease targeting chimera

Yuanqin Min *et al.*

Corresponding author: Tian Tian, ttian@whu.edu.cn

Sci. Adv. **10**, eadl4393 (2024)
DOI: 10.1126/sciadv.adl4393

The PDF file includes:

Figs. S1 to S9
General Chemical Procedures
Tables S1 and S2
General Biological Procedures
Data S1 and S2
Legend for data S3
References

Other Supplementary Material for this manuscript includes the following:

Data S3

Supplementary Figures

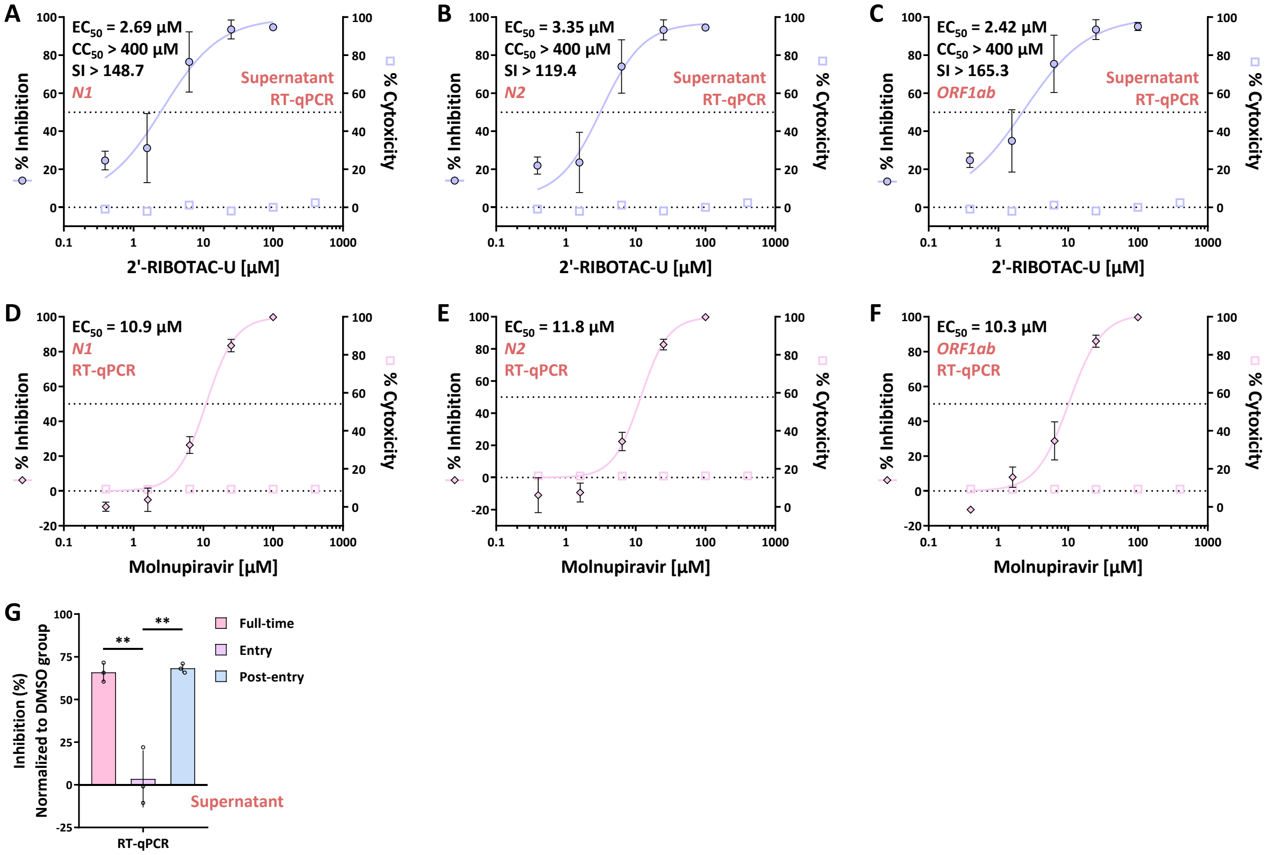

Figure S1 Nucleoside-RIBOTAC tailoring demonstrates potent efficacy against SARS-CoV-2 in Huh7-ACE2 cells

(**A-C**) demonstrate the effects of different concentrations of **2'-RIBOTAC-U** on SARS-CoV-2 infected cell samples. Post-treatment, RNA is extracted from the collected cell supernatant samples and RT-qPCR quantification analysis is performed using primers targeting different gene fragments of the virus, namely *N1*, *N2*, and *ORF1ab*. Panels (**A**), (**B**), and (**C**) correspond to *N1*, *N2*, and *ORF1ab*, respectively. The solid spherical legend represents the viral inhibition rate calculated after treatment with different concentrations of **2'-RIBOTAC-U** (normalized to the DMSO group). The hollow square legend represents the cytotoxicity test results of different concentrations of **2'-RIBOTAC-U** (showing no cytotoxicity within 400 μM, indicating good drug selectivity). In this study, the toxicity assays refer to the compound's toxicity to the cells corresponding to the culture supernatant.

(**D-F**) demonstrate the effects of different concentrations of **Molnupiravir** on SARS-CoV-2 infected cell samples. Intracellular RNA is extracted post-treatment, and RT-qPCR quantification analysis is performed using primers targeting different gene fragments of the virus, namely *N1*, *N2*, and *ORF1ab*. Panels (**D**), (**E**), and (**F**) correspond to *N1*, *N2*, and *ORF1ab*, respectively.

(**G**) represents the results of RT-qPCR analysis performed on RNA extracted from supernatant samples of cells in the time-of-compound-addition assay, evaluating the inhibitory effect of **2'-RIBOTAC-U** on SARS-CoV-2 replication. The analysis targets the N2 gene fragment of the virus.

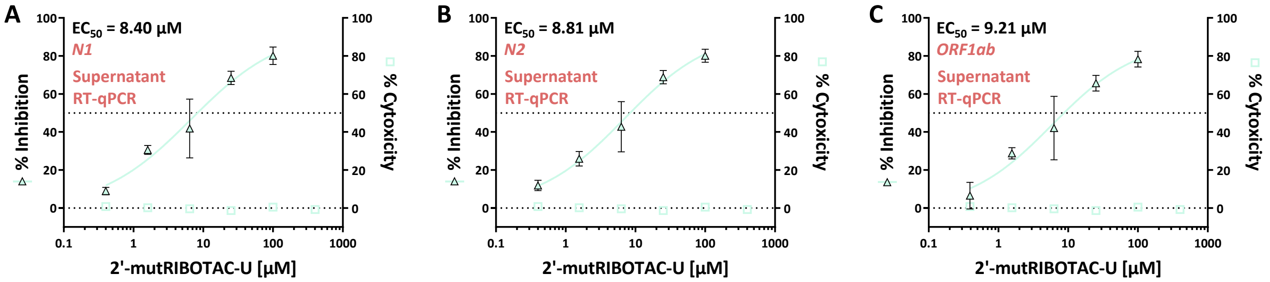

Figure S2 Structural alterations in RNase L recruiters result in a reduced activity of tailored nucleosides against SARS-CoV-2 (**A**-**C**) illustrate the effects of various concentrations of **2'-mutRIBOTAC-U** on SARS-CoV-2 infected cell samples. After treatment, RNA is extracted from the collected cell supernatant samples and RT-qPCR quantification analysis is conducted using primers targeting the virus's different gene fragments: *N1*, *N2*, and *ORF1ab*. Panels (A), (B), and (C) correspond to *N1*, *N2*, and *ORF1ab*, respectively.

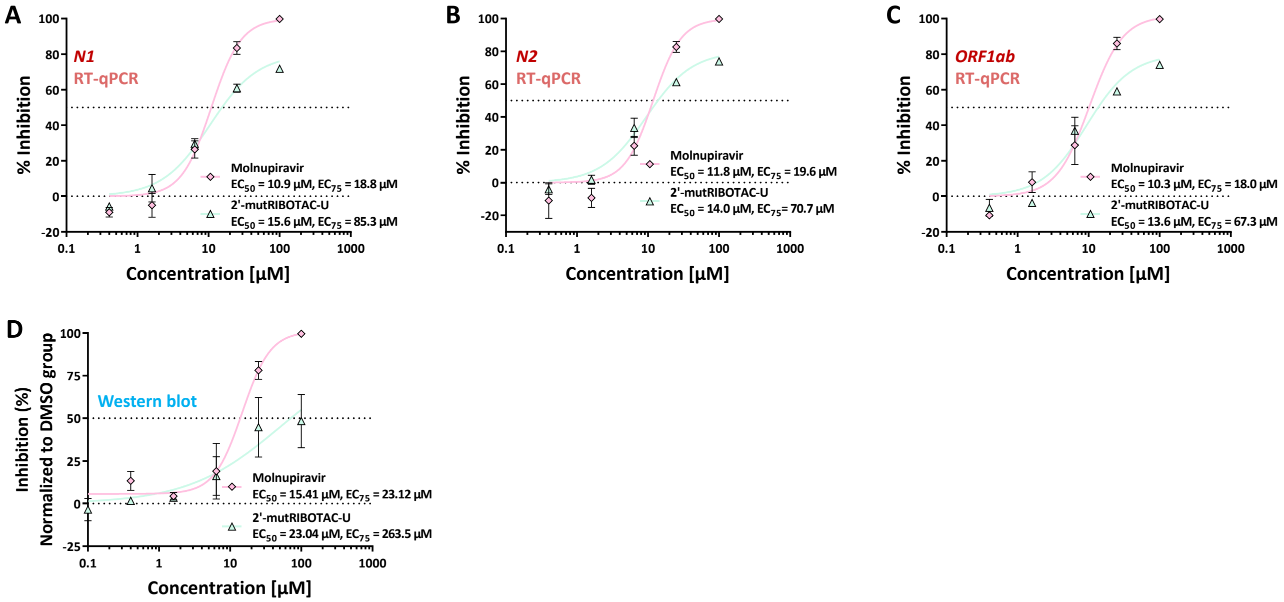

Figure S3 Comparison of the mutant (**2'-mutRIBOTAC-U**) to **Molnupiravir**.

(**A-C**): Quantitative analysis of *N1*, *N2*, and *ORF1ab* gene expression levels in infected cell samples treated with varying concentrations of **Molnupiravir** and **2'-mutRIBOTAC-U**, following RNA extraction and RT-qPCR. Solid pink diamond symbols represent the inhibition rate calculated after treatment with different concentrations of **Molnupiravir** (normalized to DMSO group), and solid green triangle symbols represent the inhibition rate after treatment with different concentrations of **2'-mutRIBOTAC-U** (normalized to DMSO). EC_{50} and EC_{75} values for the compounds were calculated based on the fitted sigmoidal curves. (D): Quantitative analysis of the Western Blot results from Figures 2G and 3E, normalized to the DMSO group, showing the inhibition rates of two compounds on N protein (**Molnupiravir** or **2'-mutRIBOTAC-U**). EC_{50} and EC_{75} values for the compounds were calculated based on the fitted curves.

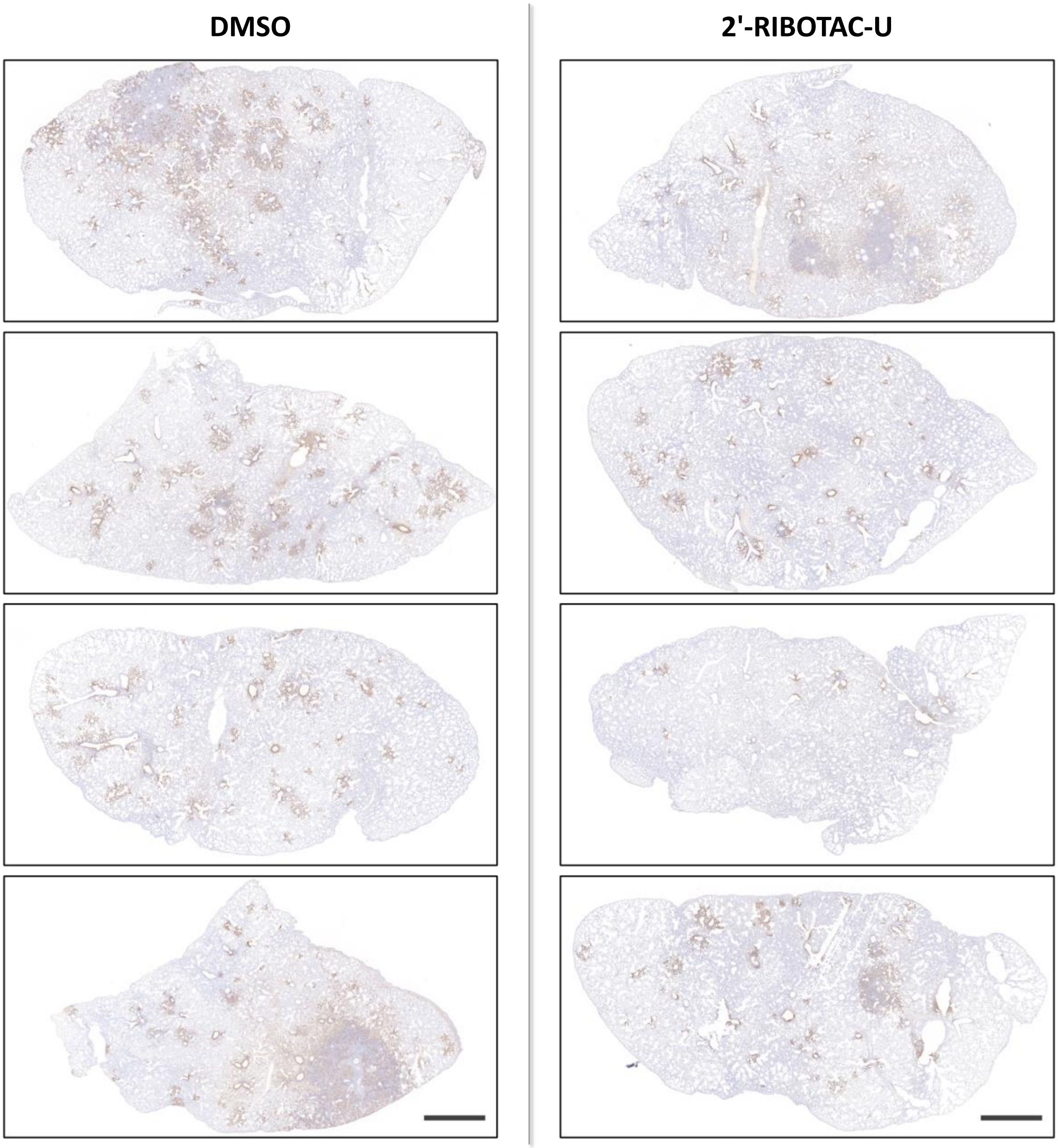

Figure S4 Representative IHC staining results of lung tissue sections from four SARS-CoV-2 infected hamsters treated with the control DMSO and four SARS-CoV-2 infected hamsters treated with **2'-RIBOTAC-U**

Yellow or brownish-yellow areas indicate SARS-CoV-2 N protein positive staining regions, with the DMSO group showing significantly more SARS-CoV-2 N positive areas than the **2'-RIBOTAC-U** treated group. Quantitative results of IHC staining from lung tissue sections of all five SARS-CoV-2 infected hamsters from both the DMSO control group and the **2'-RIBOTAC-U** treated group are presented in Figure 4F. Scale bar = 2000 μm.

A

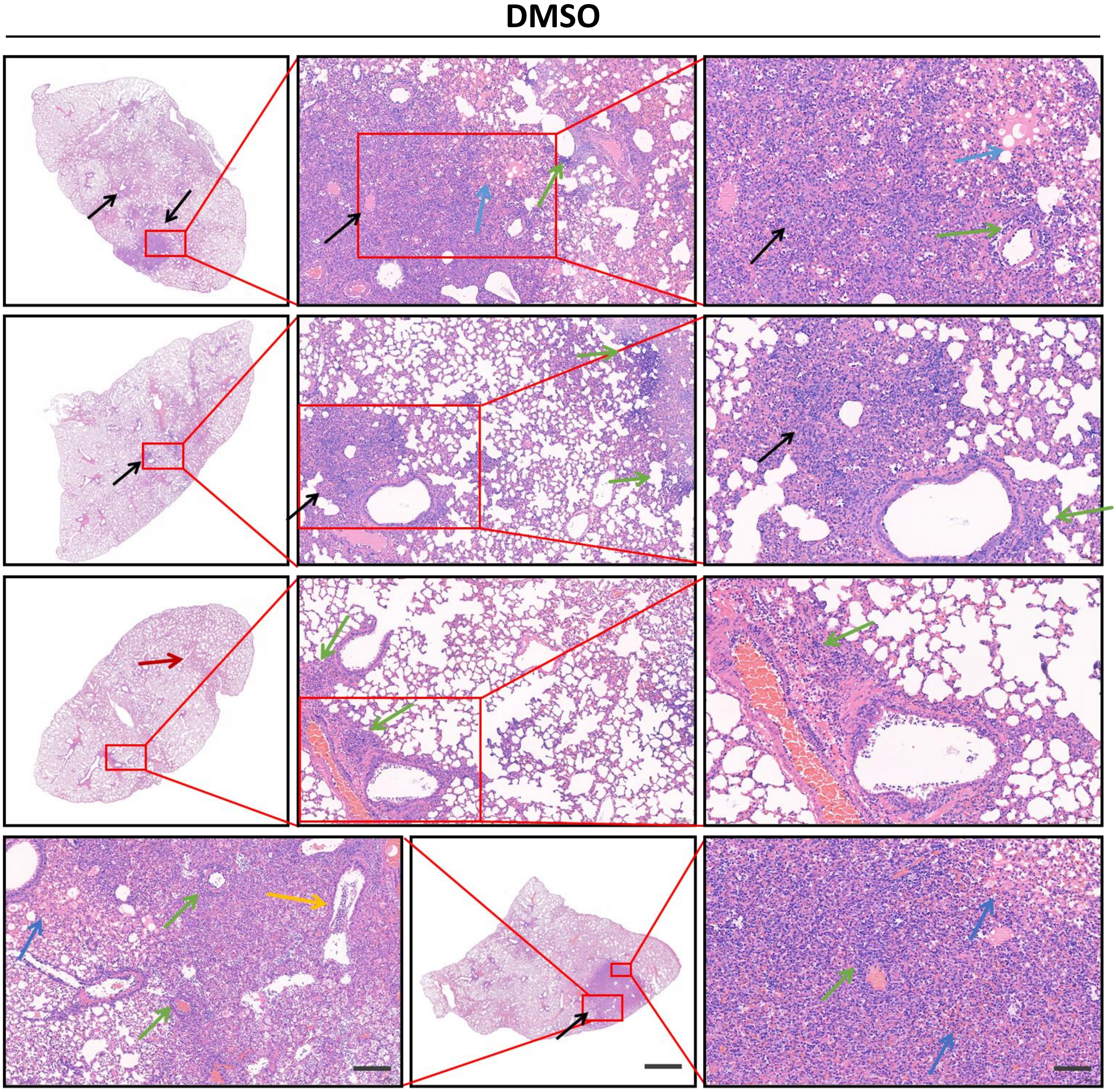

B

Group of hamsters receiving DMSO treatment

No.	PPF1	PPF2	PPF3	PPF4	PPF5	PPF6	PPF7	PPF8	PPF9	Total
H1	2	2	1	3	3	2	1	0	2	16
H2	3	2	1	2	2	0	1	1	2	14
H3	1	1	0	1	1	0	0	0	1	5
H4	1	0	1	0	0	0	0	1	2	5
H5	2	2	1	2	2	0	0	2	2	13

Figure S5 Pathological analysis results of lung tissue sections from SARS-CoV-2 infected hamsters treated with DMSO

(**A**) represents the representative pathological analysis results of lung tissue sections from four SARS-CoV-2 infected hamsters treated with DMSO:

From top to bottom, the first specimen: Visible patchy consolidation in lung tissue (black arrow), extensive inflammatory cell infiltration in the alveolar space, hemorrhage and edema (blue arrow), bronchial lumen filled with inflammatory cells and edema fluid, perivascular and peribronchial inflammatory cell cuffing (green arrow), thickened alveolar wall, and congestion.

Second specimen: Visible focal consolidation in lung tissue (black arrow), minor inflammatory cell infiltration in the alveolar space, perivascular and peribronchial inflammatory cell infiltration (green arrow), thickened alveolar wall, and congestion.

Third specimen: Small areas of thickened alveolar wall (red arrow), interstitial peribronchial inflammatory cell infiltration (green arrow).

Fourth specimen: Visible patchy consolidation in lung tissue (black arrow), increased inflammatory cell infiltration in the alveolar space, hemorrhage and edema (blue arrow), inflammatory cells seen in the bronchial lumen (yellow arrow), and cuffing-like infiltration of inflammatory cells around interstitial vessels and bronchi (green arrow).

Scale bars: 1st column - 100 μm, 2nd - 1000 μm, 3rd - 50 μm.

(**B**) represents the quantitative pathological analysis results of lung tissue sections from all five SARS-CoV-2 infected hamsters treated with the control DMSO. Different disease symptoms are graded by severity levels, with scores from 1 to 4 indicating mild, moderate, moderate-severe, and severe, respectively, and 0 indicating the absence of the pathology. PPF represents "Pulmonary Pathological Feature," evaluating the pathological manifestation in the lungs from an imaging perspective: PPF1 refers to the degree of congestion, blood stasis, and thickening of the alveolar walls; PPF2 evaluates from the perspective of the extent of pulmonary edema; PPF3 is an assessment from the standpoint of hemorrhage in the alveolar space; PPF4 gauges the degree of inflammatory cell infiltration in the alveolar cavity; PPF5 assesses the extent of lung consolidation; PPF6 indicates the degree of hemorrhage in the bronchial lumen; PPF7 examines from the perspective of edema in the bronchial lumen; PPF8 is an assessment of the degree of inflammatory cell exudation within the bronchial lumen; PPF9 evaluates based on the degree of inflammatory cell infiltration around the interstitium (surrounding blood vessels and bronchi).

A

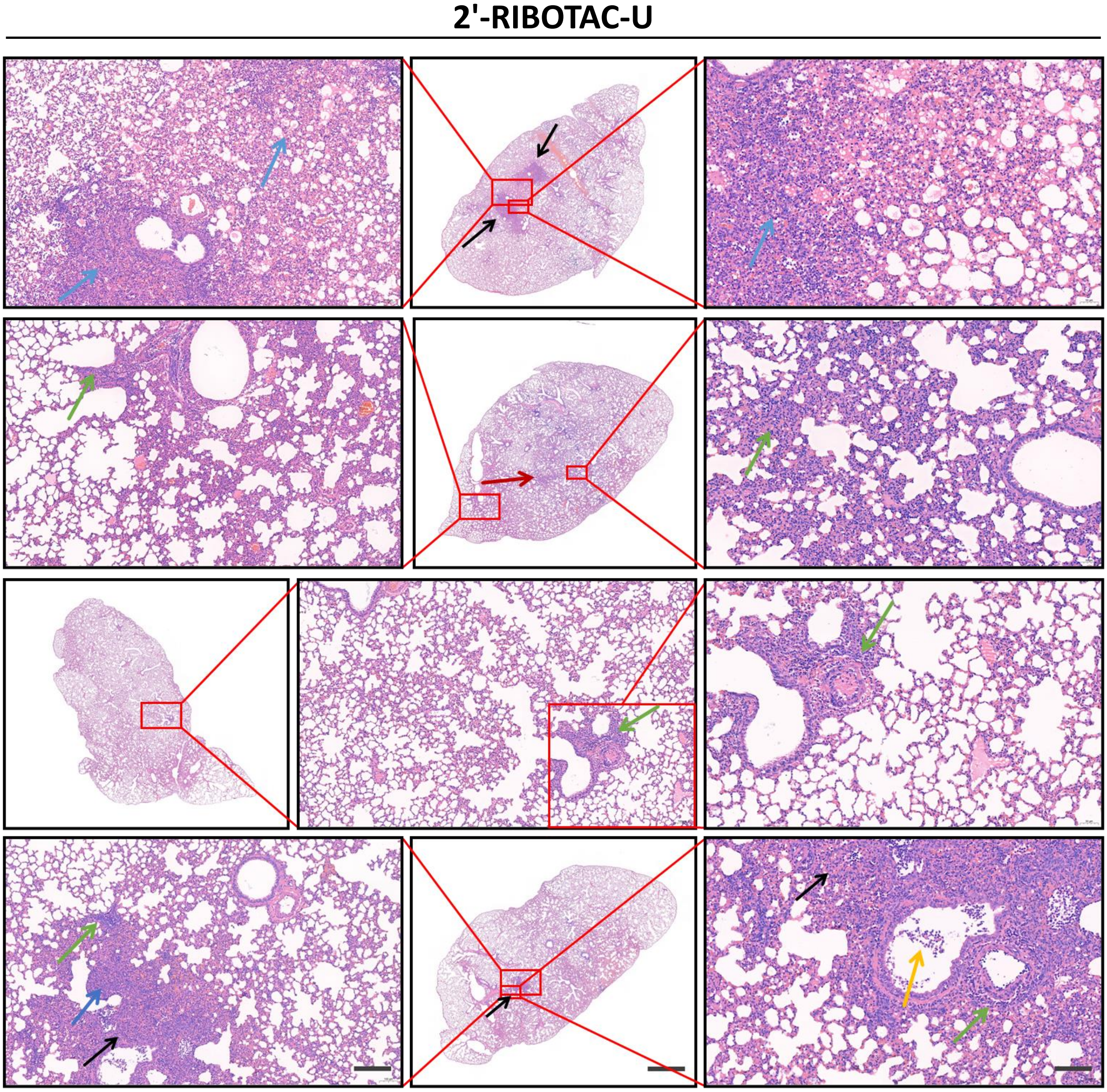

B

Group of hamsters receiving 2'-RIBOTAC-U treatment

No.	PPF1	PPF2	PPF3	PPF4	PPF5	PPF6	PPF7	PPF8	PPF9	Total
H6	1	1	1	1	1	0	0	0	1	6
H7	2	0	0	0	0	0	0	0	2	4
H8	0	0	0	0	0	0	0	0	1	1
H9	1	0	1	0	0	0	0	0	1	2
H10	1	0	1	2	1	0	0	1	1	7

Figure S6 Pathological analysis of lung tissue from SARS-CoV-2 infected hamsters treated with **2'-RIBOTAC-U**

(**A**) represents the representative pathological analysis results of lung tissue sections from four SARS-CoV-2 infected hamsters treated with **2'-RIBOTAC-U**:

From top to bottom, the first specimen: Visible small focal consolidation areas in lung tissue (black arrow), significant edema and inflammatory cell infiltration in the alveolar space (blue arrow).

Second specimen: Scattered thickening of the alveolar wall (red arrow), cuffing-like infiltration of inflammatory cells around interstitial vessels and bronchi (green arrow), thickened alveolar wall, and congestion.

Third specimen: Interstitial peribronchial inflammatory cell infiltration (green arrow).

Fourth specimen: Visible focal consolidation in lung tissue (black arrow), minor inflammatory cell infiltration in the alveolar space (blue arrow), inflammatory cells seen in the bronchial lumen (yellow arrow), and cuffing-like infiltration of inflammatory cells around interstitial vessels and bronchi (green arrow).

Scale bars: 1st column - 100 μm, 2nd - 1000 μm, 3rd - 50 μm.

(**B**) represents the quantitative pathological analysis results of lung tissue sections from all five SARS-CoV-2 infected hamsters treated with **2'-RIBOTAC-U**. The severity of different disease symptoms is graded, with scores from 1 to 4 indicating mild, moderate, moderate-severe, and severe, respectively, and 0 indicating the absence of the pathology.

General Chemical Procedures

Synthesis of 2'-RIBOTAC-U

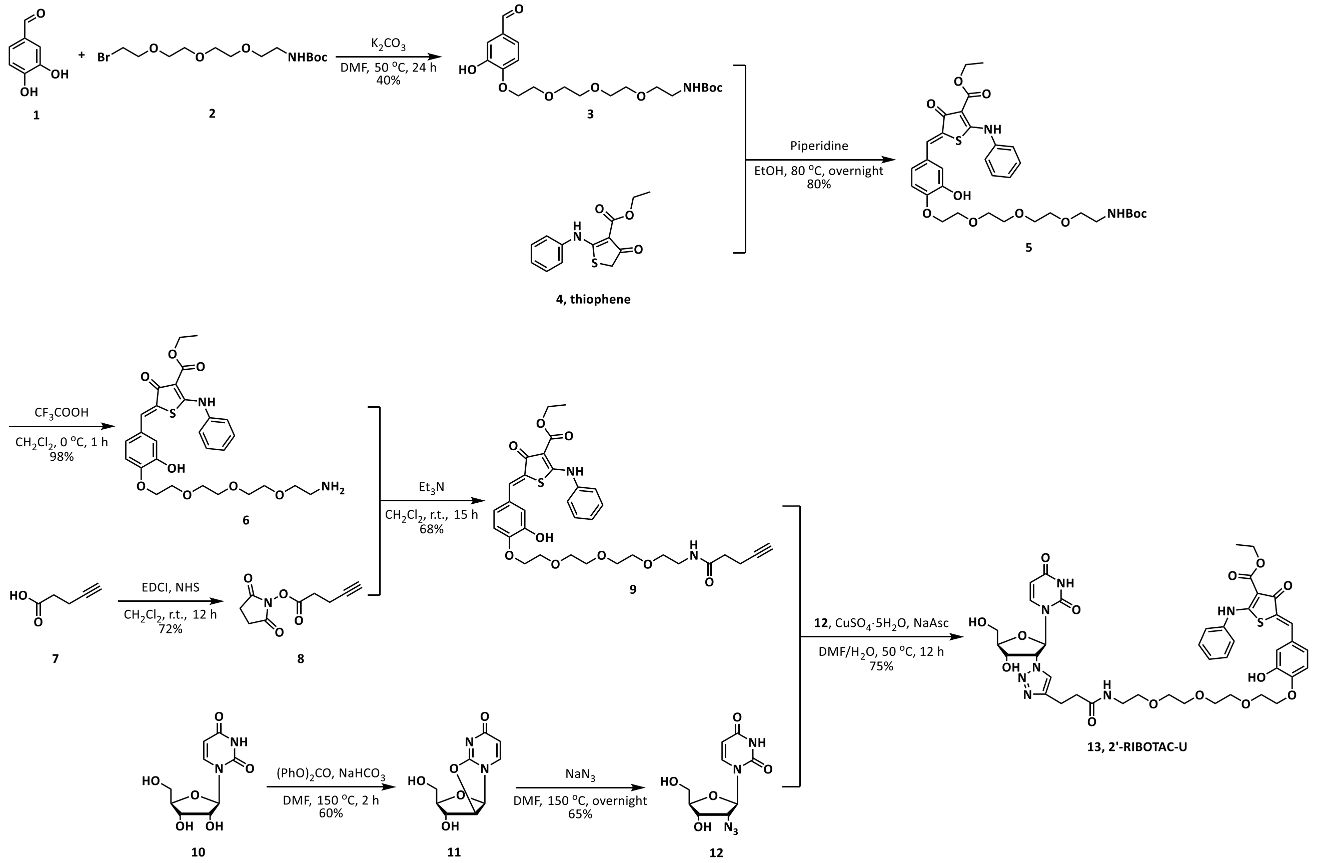

Scheme S1 General synthetic scheme for **2'-RIBOTAC-U** (Compound **5** was synthesized using a previously reported procedure(*50*))

tert-Butyl (2-(2-(2-(2-(4-formyl-2-hydroxyphenoxy)ethoxy)ethoxy)ethoxy)ethyl)carbamate (3)

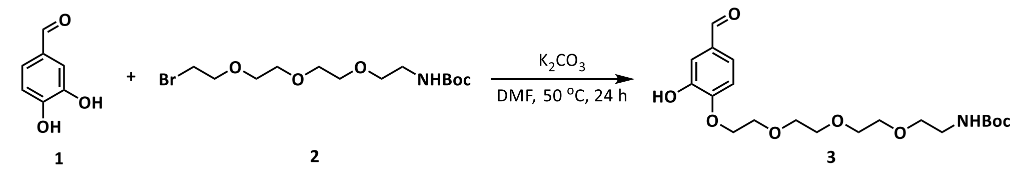

3,4-Dihydroxybenzaldehyde **1** (1.38 g, 10 mmol) and bromo-PEG-NHBoc **2** (3.56 g, 10 mmol) was dissolved in DMF (50 mL), and then K_2CO_3 (1.38 g,10 mmol) was added. The resulting mixture was stirred at 50 ˚C for 24 h. After completion, the solvent was removed under reduced pressure and the residue was purified by silica gel column chromatography (petroleum ether/AcOEt/AcOH=1/1/0.05) to give compound **3** as a light yellow oil (1.65 g, 40%). **^{1}H NMR** (400 MHz, $CDCl_3$) δ 9.84 (s, 1H), 7.45 (d, *J* = 2.0 Hz, 1H), 7.39 (dd, *J* = 8.2, 2.0 Hz, 1H), 6.99 (d, *J* = 8.2 Hz, 1H), 5.16 (s, 1H), 4.30-4.25 (m, 2H), 3.93-3.87 (m, 2H), 3.76-3.68 (m, 4H), 3.68-3.61 (m, 4H), 3.55 (t, *J* = 5.1 Hz, 2H), 3.31 (q, *J* = 5.3 Hz, 2H). **^{13}C NMR** (101 MHz, $CDCl_3$) δ 191.10, 156.08, 151.36, 147.27, 131.31, 123.73, 115.43,

112.89, 79.24, 70.68, 70.51, 70.48, 70.35, 70.21, 69.16, 69.06, 40.36, 28.42. **HRMS** (ESI$^+$) m/z calcd for $C_{20}H_{31}NNaO_8^+$ [M + Na] $^+$: 436.1942, found: 436.1937.

Ethyl (*Z*)-5-(4-((2,2-dimethyl-4-oxo-3,8,11,14-tetraoxa-5-azahexadecan-16-yl)oxy)-3-hydroxybenzylidene)-4-oxo-2-(phenylamino)-4,5-dihydrothiophene-3-carboxylate (5)

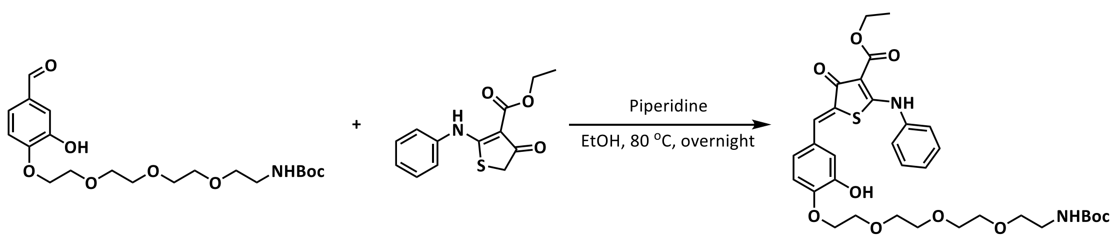

3 4 5

A solution of compound **3** (1.5 g, 3.63 mmol), ethyl 4-oxo-2-(phenylamino)-4,5-dihydrothiophene-3-carboxylate **4** (955 mg, 3.63 mmol), and piperidine (308 mg, 3.63 mmol) in EtOH (50 mL) was heated at 80 ˚C for 5 h. The reaction solution was diluted into AcOEt (200 mL) and washed with NH_4Cl (1 M, 1×200 mL) and brine (1×200 mL). The organic layer was dried over anhydrous Na_2SO_4 and concentrated under reduced pressure. The crude product was purified by silica gel column chromatography (CH_2Cl_2/MeOH=30/1) to give compound **5** as a yellow solid (1.7 g, 71%). **^{1}H NMR** (400 MHz, $CDCl_3$) δ 11.49 (s, 1H), 7.73 (s, 1H), 7.49 (dd, *J* = 8.6, 7.0 Hz, 2H), 7.42-7.35 (m, 3H), 7.24 (s, 1H), 7.12 (d, *J* = 2.2 Hz, 1H), 7.07-6.99 (m, 1H), 6.90 (d, *J* = 8.4 Hz, 1H), 5.13 (br, 1H), 4.41 (q, *J* = 7.1 Hz, 2H), 4.23-4.18 (m, 2H), 3.88-3.82 (m, 2H), 3.75-3.71 (m, 2H), 3.69 (dt, *J* = 6.0, 1.7 Hz, 2H), 3.64 (qq, *J* = 3.3, 2.1 Hz, 4H), 3.54 (t, *J* = 5.1 Hz, 2H), 3.30 (q, *J* = 5.5 Hz, 2H), 2.25 (s, 2H), 1.46-1.41 (m, 12H). **^{13}C NMR** (101 MHz, $CDCl_3$) δ 182.23, 176.23, 167.03, 156.05, 147.50, 147.30, 137.18, 131.51, 129.90, 128.55, 127.77, 125.76, 124.12, 123.52, 116.56, 114.11, 97.94, 79.19, 70.63, 70.44, 70.37, 70.22, 69.29, 69.20, 60.59, 40.35, 28.42, 14.45. **HRMS** (ESI$^+$) m/z calcd for $C_{33}H_{43}N_2O_{10}S^+$ [M + H] $^+$: 659.2633, found: 659.2628.

Ethyl-5-(4-(2-(2-(2-(2-aminoethoxy)ethoxy)ethoxy)ethoxy)-3-hydroxybenzylidene)-4-oxo-2-(phenylamino)-4,5-dihydrothiophene-3-carboxylate (6)

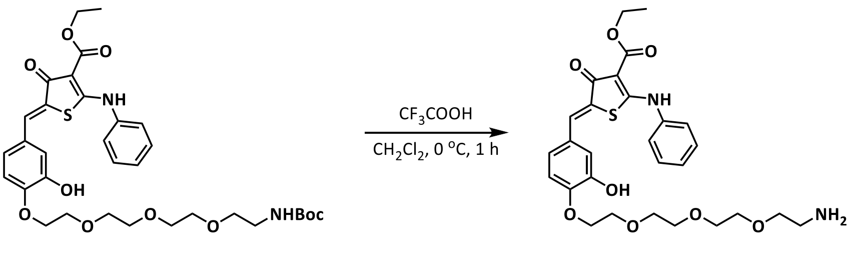

5 6

To a solution of compound **5** (1.32 g, 2.0 mmol) in anhydrous CH_2Cl_2 (50 mL) was added trifluoroacetic acid (30 mL) at 0 ˚C. The mixture was stirred at 0 ˚C for 1 h, followed by evaporation of the solvent to give the TFA salt of compound **6** (1.28 g, 98%) as a yellow solid which was used without further purification.

4-Pentynoic acid succinimidyl ester (8)(*60*)

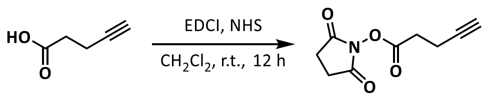

7 **8**

To a solution of 4-pentynoic acid **7** (980 mg, 10 mmol) and EDCI (3.83 g, 20 mmol) in CH_2Cl_2 (100 mL) was added N-hydroxysuccinimide (1.2 g, 10.5 mmol) at 0 °C. Then the resulting mixture was stirred at room temperature for 5 h. After completion of the reaction, the reaction solution was diluted with CH_2Cl_2 (100 mL) and the organic phase was washed with a 0.2 M $NaHSO_4$ solution and brine. The organic layer was dried over Na_2SO_4 and concentrated under reduced pressure to afford 4-pentynoic acid succinimidyl ester **8** (1.59 g, 82% yield) as a white crystalline solid. **^{1}H NMR** (400 MHz, $CDCl_3$) δ 2.88 (dd, *J* = 15.5, 7.7 Hz, 6H), 2.62 (ddd, *J* = 8.7, 6.6, 2.7 Hz, 2H), 2.05 (t, *J* = 2.6 Hz, 1H). **^{13}C NMR** (101 MHz, $CDCl_3$) δ 168.93, 167.03, 80.85, 70.04, 30.31, 25.57, 14.09. **HRMS** (ESI^+) m/z calcd for $C_9H_{10}NO_4^+$ [M + H] $^+$: 196.0604, found: 196.0599.

Ethyl (Z)-5-(3-hydroxy-4-((13-oxo-3,6,9-trioxa-12-azaheptadec-16-yn-1-yl)oxy)benzylidene)-4-oxo-2-(phenylamino)-4,5-dihydrothiophene-3-carboxylate (9)

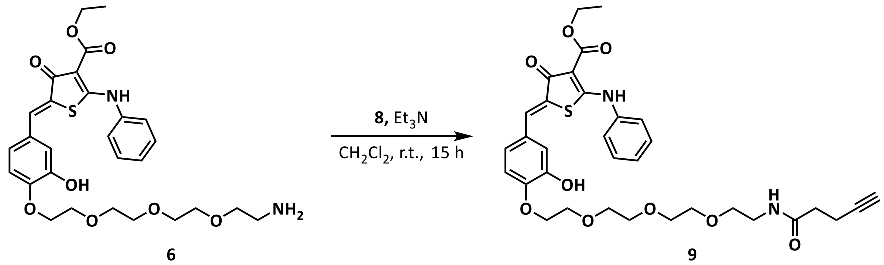

6 **9**

All compound **6** (1.28 g, 1.96 mmol) and compound **8** (390 mg, 2.0 mmol) were dissolved in anhydrous CH_2Cl_2 (50 mL) at 0 °C and Et_3N (404 mg, 4.0 mmol) was added slowly via a syringe. The resulting mixture was stirred at room temperature for 15 h. The reaction mixture was diluted into CH_2Cl_2 (150 mL) and washed with NH_4Cl (1 M, 1×200 mL), brine (1×200 mL). The organic phase was separated and dried over anhydrous Na_2SO_4. After filtration and evaporation of the solvent at reduced pressure, the crude product was purified by silica gel column chromatography (CH_2Cl_2/MeOH =30/1) to give compound **9** (850 mg, 68%) as a yellow oil. **^{1}H NMR** (400 MHz, $CDCl_3$) δ 11.49 (s, 1H), 7.84 (br, 1H), 7.73 (s, 1H), 7.53-7.47 (m, 2H), 7.42-7.37 (m, 3H), 7.12 (d, *J* = 2.2 Hz, 1H), 7.02 (dd, *J* = 8.5, 2.2 Hz, 1H), 6.88 (d, *J* = 8.4 Hz, 1H), 6.45 (t, *J* = 5.5 Hz, 1H), 4.41 (q, *J* = 7.1 Hz, 2H), 4.22-4.18 (m, 2H), 3.88-3.83 (m, 2H), 3.75-3.67 (m, 4H), 3.63 (s, 4H), 3.57 (dd, *J* = 5.6, 4.3 Hz, 2H), 3.49-3.44 (m, 2H), 2.51-2.45 (m, 2H), 2.41-2.36 (m, 2H), 1.99 (t, *J* = 2.6 Hz, 1H), 1.44 (t, *J* = 7.1 Hz, 3H). **^{13}C NMR** (101 MHz, $CDCl_3$) δ 182.23, 176.22, 171.21, 167.00, 147.56, 147.15, 137.17, 131.48, 129.90, 128.46, 127.81, 125.73, 124.15, 124.12, 123.48, 116.79, 113.57, 97.94, 83.10, 70.58, 70.31, 70.24, 70.19, 69.25, 68.58, 60.59, 39.32, 35.17, 14.45. **HRMS** (ESI^+) m/z calcd for $C_{33}H_{39}N_2O_9S^+$ [M + H] $^+$: 639.2371, found: 639.2381.

2,2'-Cyclouridine (11)(*61*)

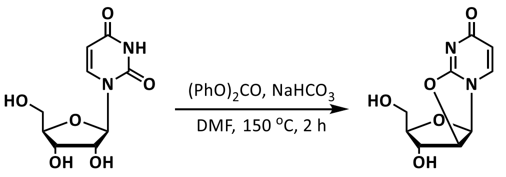

10 **11**

Uridine **10** (2.44 g, 10 mmol) and diphenyl carbonate (2.35 g, 11 mmol) were dissolved in anhydrous DMF (100 mL). The solution was heated to 100 °C and sodium bicarbonate (100 mg) was then added. The resulting mixture was heated up to 150 °C for 2 h. After completion, the reaction was cooled to room temperature and then concentrated under reduced pressure. The dried residue was purified by silica gel column chromatography (CH_2Cl_2 /MeOH =10/1) to afford 2,2'-cyclouridine **11** (1.36 g, 60% yield) as a white solid. **^{1}H NMR** (400 MHz, DMSO-*d*$_6$) δ 7.84 (d, *J* = 7.4 Hz, 1H), 6.31 (d, *J* = 5.7 Hz, 1H), 5.88 (d, *J* = 4.3 Hz, 1H), 5.84 (d, *J* = 7.4 Hz, 1H), 5.20 (d, *J* = 5.7 Hz, 1H), 4.98 (t, *J* = 5.2 Hz, 1H), 4.41-4.37 (m, 1H), 4.07 (ddd, *J* = 6.5, 5.2, 1.7 Hz, 1H), 3.33-3.15 (m, 2H). **^{13}C NMR** (101 MHz, DMSO-*d*$_6$) δ 171.65, 160.27, 137.31, 109.08, 90.48, 89.67, 89.21, 75.20, 61.30. **HRMS** (ESI$^+$) m/z calcd for $C_9H_{11}N_2O_6^+$ [M + H]$^+$: 243.0612, found: 243.0610.

2'-Azido-2'-deoxyuridine (12)(*62*)

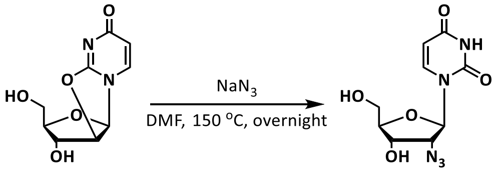

11 12

To a solution of 2,2'-cyclouridine **11** (1.13 g, 5 mmol) in anhydrous DMF (30 mL) was added sodium azide (650 mg, 10 mmol). The reaction mixture was refluxed at 150 °C overnight. After completion, the solvent was removed under reduced pressure. The crude product was purified by silica gel column chromatography (CH_2Cl_2 /MeOH =10/1) to afford 2'-azido-2'-deoxyuridine **12** (914 mg, 68% yield) as an offwhite solid. **^{1}H NMR** (400 MHz, DMSO-*d*$_6$) δ 11.42 (s, 1H), 7.87 (d, *J* = 8.1 Hz, 1H), 5.96 (d, *J* = 5.5 Hz, 1H), 5.88 (d, *J* = 5.5 Hz, 1H), 5.68 (dd, *J* = 8.1, 2.0 Hz, 1H), 5.18 (t, *J* = 5.1 Hz, 1H), 4.30 (q, *J* = 5.2 Hz, 1H), 4.05 (t, *J* = 5.5 Hz, 1H), 3.89 (dt, *J* = 4.6, 3.1 Hz, 1H), 3.62 (dddd, *J* = 35.3, 12.2, 5.2, 3.2 Hz, 2H). **^{13}C NMR** (101 MHz, DMSO-*d*$_6$) δ 150.92, 140.50, 102.54, 86.05, 85.69, 70.92, 65.05, 60.68. **HRMS** (ESI$^+$) m/z calcd for $C_9H_{12}N_5O_5^+$ [M + H]$^+$: 270.0833, found: 270.0836.

2'-RIBOTAC-U (13)

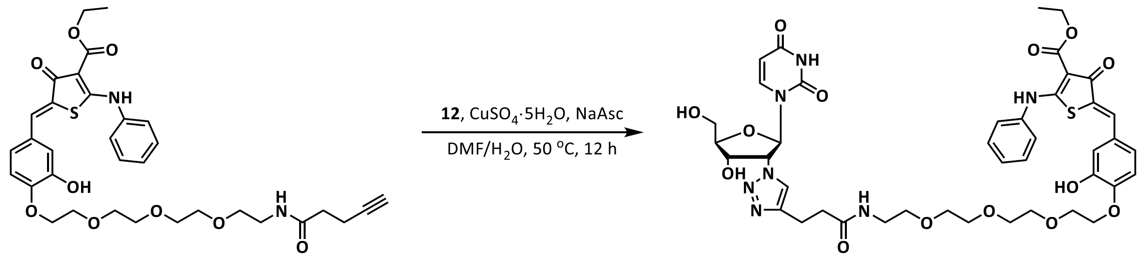

9 13, 2'-RIBOTAC-U

To a solution of 2'-azido-2'-deoxyuridine **12** (269 mg, 1.0 mmol) and compound **9** (638 mg, 1.0 mmol) in DMF (20 mL), sodium ascorbate (990 mg, 5.0 mmol) in water (5 mL) and copper (II) sulfate pentahydrate (249 mg, 1.0 mmol) in water (5 mL) were added respectively. Thereafter, the reaction mixture was stirred at 50 °C for 12 h. After completion, the solvent was removed under reduced pressure and the residue was purified by silica gel column chromatography (MeOH/CH_2Cl_2=1/15) to afford the desired product **2'-RIBOTAC-U (13)** (680 mg, 75% yield) as a light yellow solid. **^{1}H NMR** (400 MHz, Methanol-*d*$_4$) δ 8.03 (d, *J* = 8.1 Hz, 1H), 7.80 (s, 1H), 7.54 (s, 1H), 7.49-7.32 (m, 5H), 6.93-6.89 (m, 2H), 6.47 (d, *J* = 6.5 Hz, 1H), 5.63 (d, *J* = 8.1 Hz, 1H), 5.30 (t, *J* = 6.2 Hz, 1H), 4.47 (dd, *J* = 5.9, 3.9 Hz, 1H), 4.29 (q, *J* = 7.1 Hz, 2H), 4.16 (dd, *J* = 4.5, 2.1 Hz, 1H), 4.13-4.05 (m, 2H), 3.85-3.70 (m, 4H), 3.64-3.48 (m, 8H), 3.40 (t, *J* = 5.5 Hz, 2H), 3.27 (s, 1H), 3.26-3.24 (m, 2H), 2.90 (t, *J* = 7.4 Hz, 2H), 2.46 (t, *J* = 7.4 Hz, 2H), 1.30 (t, *J* = 7.1 Hz, 3H). **^{13}C NMR** (101 MHz, Methanol-*d*$_4$) δ 183.06, 176.68, 173.21, 165.70, 164.40, 150.65, 148.79, 146.97, 146.00, 140.45, 137.34, 131.70, 129.56, 128.03,

127.05, 124.89, 124.74, 123.47, 123.36, 116.13, 112.93, 101.92, 97.55, 86.54, 86.45, 70.18, 70.15, 70.13, 70.05, 69.76, 69.15, 69.11, 67.90, 65.90, 60.80, 59.99, 38.94, 34.86, 21.17, 13.44. **HRMS** (ESI$^+$) m/z calcd for $C_{42}H_{50}N_7O_{14}S^+$ [M + H] $^+$: 908.3131, found: 908.3150.

Synthesis of 2'-mutRIBOTAC-U

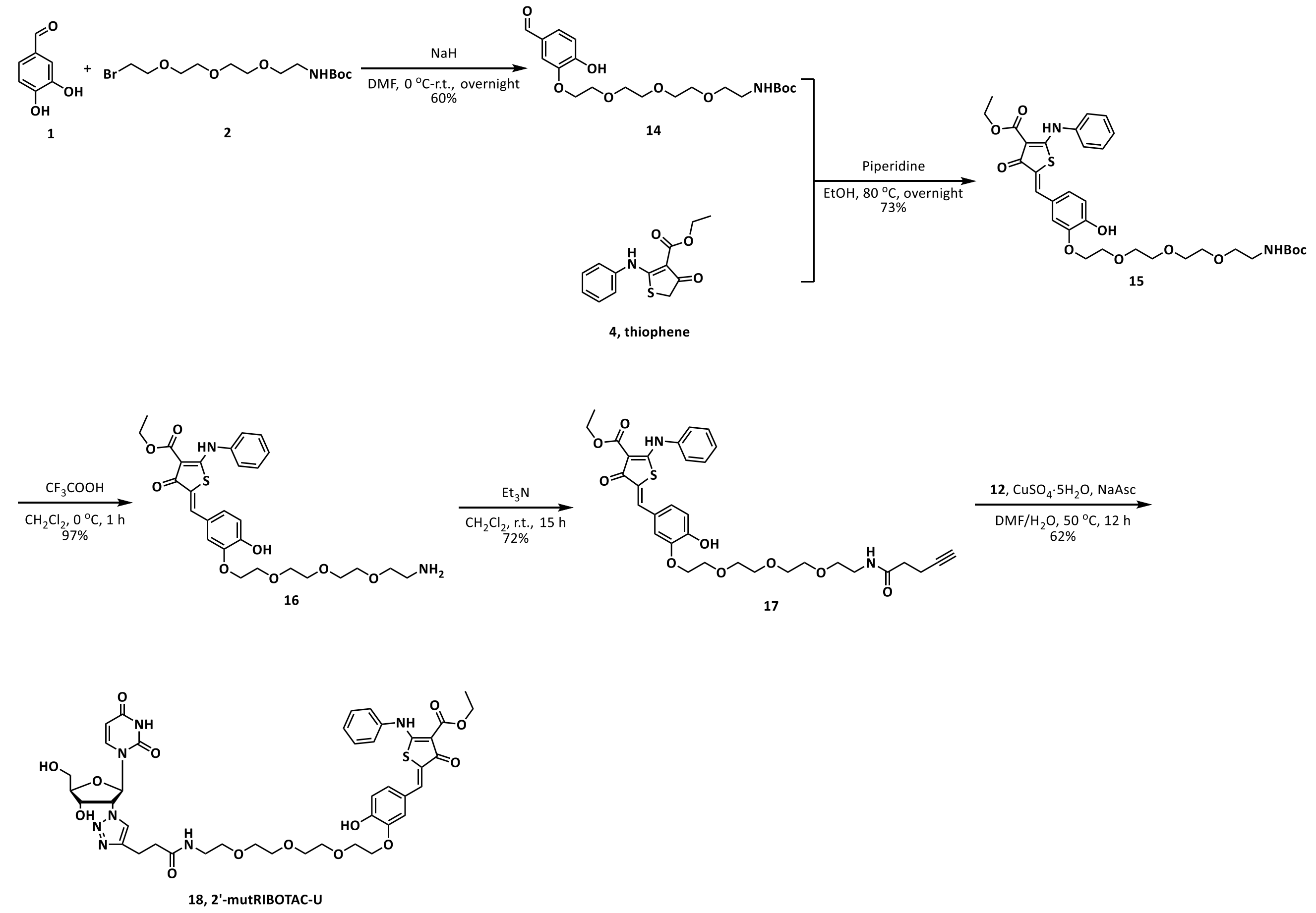

Scheme S2 General synthetic scheme for **2'-mutRIBOTAC-U** (Compound **5** was synthesized using a previously reported procedure(*50*))

***tert*-Butyl (2-(2-(2-(2-(5-formyl-2-hydroxyphenoxy)ethoxy)ethoxy)ethoxy)ethyl)carbamate (14)**

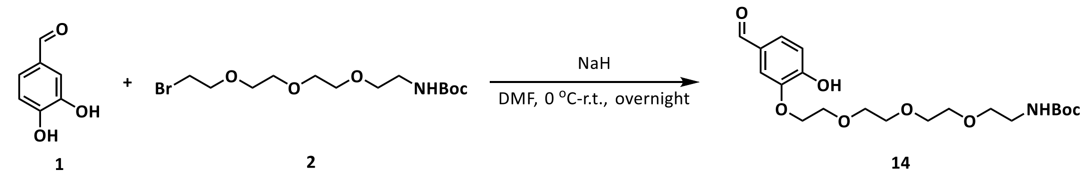

To a solution of NaH (880 mg, 60% oil dispersed, 22 mmol) in anhydrous DMF (20 mL) was added a solution of 3,4-dihydroxybenzaldehyde **1** (1.38 g, 10 mmol) in anhydrous DMF (20 mL) at 0 ˚C. After stirring for 30 min, bromo-PEG-NHBoc **2** (3.56 g, 10 mmol) in anhydrous DMF (20 mL) was added. The mixture was and stirred at room temperature overnight. The reaction was quenched by the addition of 2 eq. of acetic acid. The solvent was removed under reduced pressure and the residue was purified by silica gel column chromatography (petroleum ether/AcOEt/AcOH=1/1/0.05) to give compound **14** as a light yellow oil (2.68 g, 60%). **^{1}H NMR** (400 MHz, $CDCl_3$) δ 9.80 (s, 1H), 7.45 (dq, *J* = 3.9, 1.9 Hz, 2H), 7.04 (d, *J* = 8.5 Hz, 1H), 5.17 (br, 1H), 4.27-4.21 (m, 2H), 3.90-3.84

(m, 2H), 3.77-3.72 (m, 2H), 3.70 (dt, J = 6.1, 1.7 Hz, 2H), 3.65 (tt, J = 5.0, 2.8 Hz, 4H), 3.55 (t, J = 5.1 Hz, 2H), 3.32 (q, J = 5.4 Hz, 2H), 1.43 (s, 9H). **^{13}C NMR** (101 MHz, $CDCl_3$) δ 190.76, 156.07, 153.54, 146.69, 129.47, 128.10, 115.75, 112.78, 79.20, 70.56, 70.43, 70.40, 70.37, 70.22, 69.28, 69.22, 40.35, 28.42. **HRMS** (ESI$^+$) m/z calcd for $C_{20}H_{31}NNaO_8^+$ [M + Na] $^+$: 436.1942, found: 436.1939.

Ethyl-5-(3-((2,2-dimethyl-4-oxo-3,8,11,14-tetraoxa-5-azahexadecan-16-yl)oxy)-4-hydroxybenzylidene)-4-oxo-2-(phenylamino)-4,5-dihydrothiophene-3-carboxylate (15)

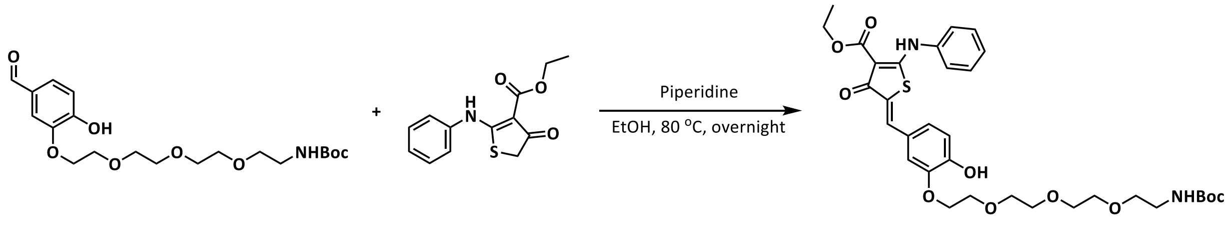

14 **4** **15**

A solution of compound **14** (2.1 g, 4.0 mmol), ethyl 4-oxo-2-(phenylamino)-4,5-dihydrothiophene-3-carboxylate **4** (1.05 g, 4.0 mmol), and piperidine (340 mg, 4.0 mmol) in EtOH (50 mL) was heated at 80 ˚C for 5 h. The reaction solution was diluted into AcOEt (200 mL) and washed with NH_4Cl (1 M, 1×200 mL) and brine (1×200 mL). The organic layer was dried over anhydrous Na_2SO_4 and concentrated under reduced pressure. The crude product was purified by silica gel column chromatography (CH_2Cl_2/MeOH=30/1) to give compound 1**5** as a yellow solid (1.92 g, 73%). **^{1}H NMR** (400 MHz, $CDCl_3$) δ 11.48 (s, 1H), 7.72 (s, 1H), 7.52-7.46 (m, 2H), 7.43-7.34 (m, 3H), 7.16-7.12 (m, 1H), 7.06-7.02 (m, 1H), 6.98-6.92 (m, 1H), 5.16 (br, 1H), 4.41 (qd, J = 7.2, 1.2 Hz, 2H), 4.19-4.13 (m, 2H), 3.87-3.81 (m, 2H), 3.76-3.68 (m, 4H), 3.66-3.62 (m, 4H), 3.56-3.62 (m, 2H), 3.31 (q, J = 5.4 Hz, 2H), 1.46-1.41 (m, 12H). **^{13}C NMR** (101 MHz, $CDCl_3$) δ 182.25, 176.02, 167.03, 156.06, 149.55, 146.28, 137.24, 131.62, 129.84, 127.74, 126.14, 125.29, 124.85, 124.12, 117.85, 116.53, 98.03, 79.18, 70.59, 70.45, 70.39, 70.36, 70.22, 69.97, 69.36, 60.62, 40.35, 28.42, 14.46. **HRMS** (ESI$^+$) m/z calcd for $C_{33}H_{43}N_2O_{10}S^+$ [M + H] $^+$: 659.2633, found: 659.2630.

Ethyl-5-(3-(2-(2-(2-(2-aminoethoxy)ethoxy)ethoxy)ethoxy)-4-hydroxybenzylidene)-4-oxo-2-(phenylamino)-4,5-dihydrothiophene-3-carboxylate (16)

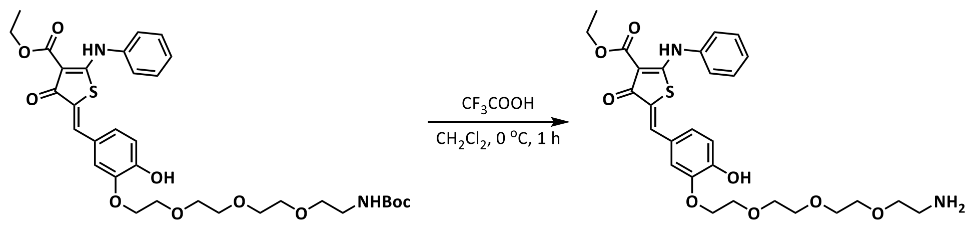

15 **16**

To a solution of compound **15** (1.64 g, 2.5 mmol) in anhydrous CH_2Cl_2 (50 mL) was added trifluoroacetic acid (30 mL) at 0 ˚C. The mixture was stirred at 0 ˚C for 1 h, followed by evaporation of the solvent to give the TFA salt of compound **16** (1.59 g, 97%) as a yellow solid which was used without further purification.

Ethyl-5-(4-hydroxy-3-((13-oxo-3,6,9-trioxa-12-azaheptadec-16-yn-1-yl)oxy)benzylidene)-4-oxo-2-(phenylamino)-4,5-dihydrothiophene-3-carboxylate (17)

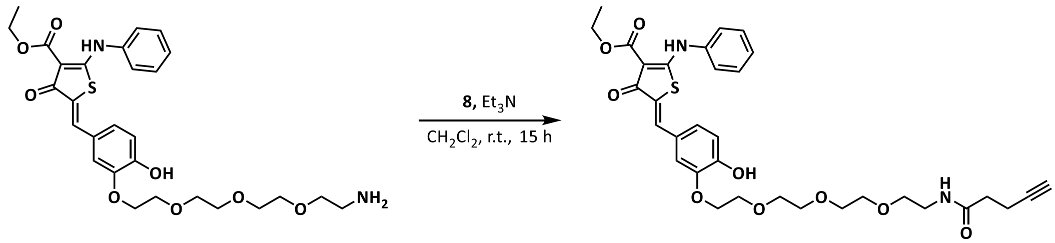

Compound 16 (1.31 g, 2.0 mmol) and compound **8** (410 mg, 2.1 mmol) were dissolved in anhydrous CH_2Cl_2 (50 mL) at 0 °C and Et_3N (404 mg, 4.0 mmol) was added slowly via a syringe. The resulting mixture was stirred at room temperature for 15 h. The reaction mixture was diluted into CH_2Cl_2 (150 mL) and washed with NH_4Cl (1 M, 1×200 mL), brine (1×200 mL). The organic phase was separated and dried over anhydrous Na_2SO_4. After filtration and evaporation of the solvent at reduced pressure, the crude product was purified by silica gel column chromatography (CH_2Cl_2/MeOH =30/1) to give compound **17** (918 mg, 72%) as a yellow oil. **^{1}H NMR** (400 MHz, $CDCl_3$) δ 11.49 (s, 1H), 8.34 (s, 1H), 7.72 (br, 1H), 7.52-7.46 (m, 2H), 7.43-7.37 (m, 3H), 7.14 (dd, *J* = 8.3, 2.1 Hz, 1H), 7.03 (d, *J* = 2.1 Hz, 1H), 6.94 (d, *J* = 8.3 Hz, 1H), 6.49 (br, 1H), 4.41 (q, *J* = 7.1 Hz, 2H), 4.20-4.15 (m, 2H), 3.87-3.83 (m, 2H), 3.76-3.72 (m, 2H), 3.69 (dt, *J* = 6.6, 2.0 Hz, 2H), 3.64 (s, 4H), 3.60-3.56 (m, 2H), 3.50-3.44 (m, 2H), 2.52-2.46 (m, 2H), 2.42-2.36(m, 2H), 1.97 (t, *J* = 2.6 Hz, 1H), 1.44 (t, *J* = 7.1 Hz, 3H). **^{13}C NMR** (101 MHz, $CDCl_3$) δ 182.27, 176.03, 171.27, 166.99, 149.26, 146.31, 137.22, 131.64, 129.84, 127.77, 126.13, 124.89, 124.87, 124.12, 117.09, 116.62, 98.04, 83.12, 70.55, 70.30, 70.19, 69.33, 69.15, 69.07, 60.63, 39.34, 35.14, 14.46. **HRMS** (ESI^+) m/z calcd for $C_{33}H_{39}N_2O_9S^+$ [M + H] $^+$: 639.2371, found: 639.2382.

2'-mutRIBOTAC-U (18)

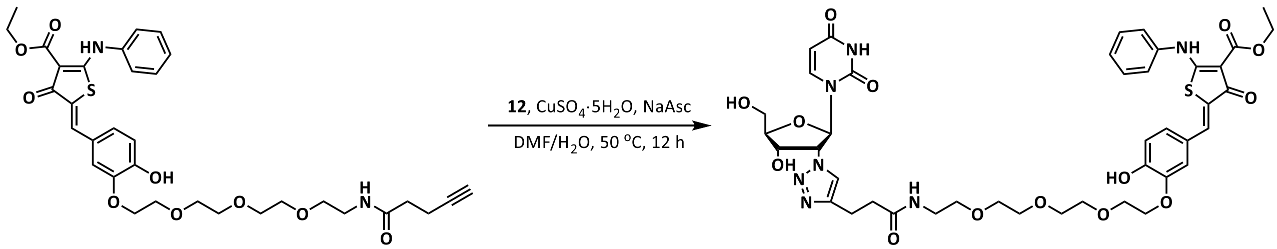

To a solution of 2'-azido-2'-deoxyuridine **12** (269 mg, 1.0 mmol) and compound **17** (638 mg, 1.0 mmol) in DMF (20 mL), sodium ascorbate (990 mg, 5.0 mmol) in water (5 mL) and copper (II) sulfate pentahydrate (249 mg, 1.0 mmol) in water (5 mL) were added respectively. Thereafter, the reaction mixture was stirred at 50 °C for 12 h. After completion, the solvent was removed under reduced pressure and the residue was purified by silica gel column chromatography (MeOH/CH_2Cl_2=1/15) to afford the desired product **2'-mutRIBOTAC-U (18)** (562 mg, 62%) as a yellow solid. **^{1}H NMR** (400 MHz, Methanol-*d*$_4$) δ 8.00 (d, *J* = 8.1 Hz, 1H), 7.76 (s, 1H), 7.56 (s, 1H), 7.45-7.31 (m, 5H), 7.01 (d, *J* = 2.1 Hz, 1H), 6.95 (dd, *J* = 8.3, 2.1 Hz, 1H), 6.75 (d, *J* = 8.3 Hz, 1H), 6.44 (d, *J* = 6.5 Hz, 1H), 5.61 (d, *J* = 8.1 Hz, 1H), 5.27 (t, *J* = 6.2 Hz, 1H), 4.45 (dd, *J* = 5.9, 3.9 Hz, 1H), 4.26 (q, *J* = 7.1 Hz, 2H), 4.13 (dt, *J* = 4.0, 2.7 Hz, 1H), 4.07-4.01 (m, 2H), 3.82-3.68 (m, 4H), 3.58-3.45 (m, 8H), 3.38 (t, *J* = 5.5 Hz, 2H), 3.25 (s, 2H), 2.87 (t, *J* = 7.4 Hz, 2H), 2.43 (t, *J* = 7.5 Hz, 2H), 1.28 (t, *J* = 7.1 Hz, 3H). **^{13}C NMR** (101 MHz, Methanol-*d*$_4$) δ 183.07, 176.51, 173.20, 165.72, 164.40, 150.64, 149.45, 146.95, 140.44, 137.37, 132.02, 129.52, 127.97, 125.50, 124.64, 124.37, 124.27, 123.36, 115.97, 115.78, 101.92, 97.63, 86.55, 86.44, 70.18, 70.17, 70.13, 70.07, 69.76, 69.26, 69.10, 68.20, 65.90, 60.80, 59.99, 38.94, 34.86, 21.17, 13.44. **HRMS** (ESI^+) m/z calcd for $C_{42}H_{50}N_7O_{14}S^+$ [M + H] $^+$: 908.3131, found: 908.3153.

Synthesis of 5-RIBOTAC-U

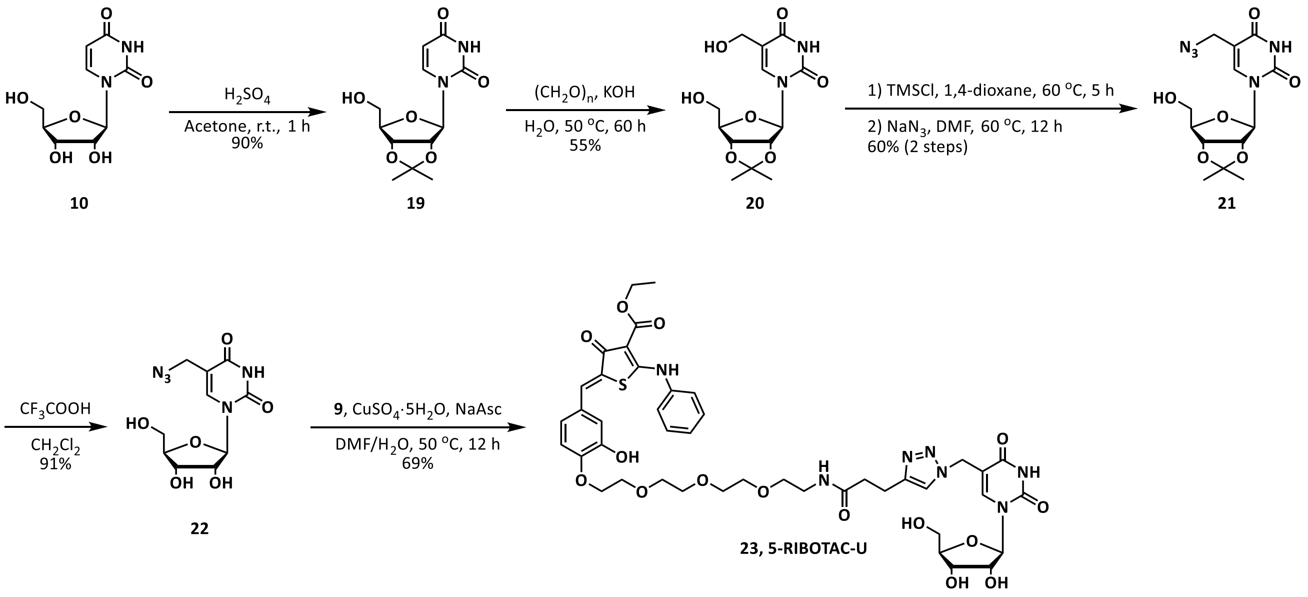

Scheme S3 General synthetic scheme for **5-RIBOTAC-U**

2',3'-O-Isopropylideneuridine (19)(*63*)

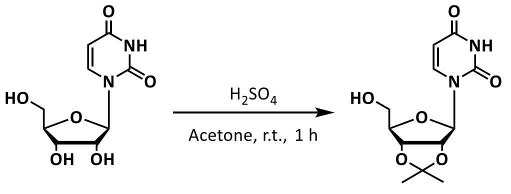

10 19

To a stirred suspension of uridine **10** (2.44 g, 10 mmol) in dry acetone (100 mL) was added H_2SO_4 (2 mL) dropwise at room temperature. The resulting mixture was stirred for 1 h and neutralized with Et_3N. Then the solvent was removed under reduced pressure and the residue was purified by column chromatography (MeOH/CH_2Cl_2=1/15) to give 2',3'-O-isopropylidenuridine **19** (2.55 g, 90% yield) as a white solid. **^{1}H NMR** (400 MHz, DMSO-d_6) δ 11.38 (d, *J* = 2.3 Hz, 1H), 7.79 (d, *J* = 8.1 Hz, 1H), 5.83 (d, *J* = 2.6 Hz, 1H), 5.64 (dd, *J* = 8.0, 2.2 Hz, 1H), 5.08 (br, 1H), 4.89 (dd, *J* = 6.4, 2.7 Hz, 1H), 4.74 (dd, *J* = 6.3, 3.6 Hz, 1H), 4.06 (q, *J* = 4.3 Hz, 1H), 3.63-3.52 (m, 2H), 1.48 (s, 3H), 1.29 (s, 3H). **^{13}C NMR** (101 MHz, DMSO-d_6) δ 163.65, 150.81, 142.40, 113.43, 102.20, 91.58, 86.99, 84.15, 80.94, 61.73, 27.52, 25.65. **HRMS** (ESI$^+$) m/z calcd for $C_{12}H_{17}N_2O_6^+$ [M + H]$^+$: 285.1081, found: 285.1084.

5-Hydroxymethyl-2',3'-O-isopropylideneuridine (20)(*64*)

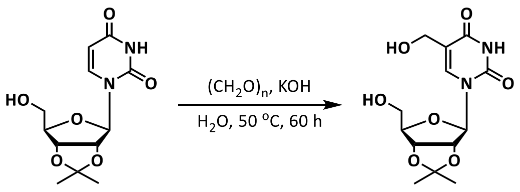

19 20

To a solution of 2',3'-O-isopropylidenuridine **19** (2.5 g, 8.8 mmol) in 0.5 N KOH (100 mL), paraformaldehyde (5.0 g) was added. The mixture was stirred at 50 °C for 60 h. The solvent was removed under reduced pressure and the residue was purified by column chromatography (MeOH/CH_2Cl_2=1/10) to give 5-hydroxymethyl-2',3'-O-isopropylideneuridine **20** (1.52 g, 55% yield) as a white solid. **^{1}H NMR** (400 MHz, DMSO-*d*$_6$) δ 11.41 (s, 1H), 7.69 (s, 1H), 5.86 (d, *J* = 2.9 Hz, 1H), 5.05 (t, *J* = 5.3 Hz, 1H), 4.93-4.83 (m, 2H), 4.75 (dd, *J* = 6.4, 3.6 Hz, 1H), 4.17-4.09 (m, 2H), 4.06 (d, *J* = 4.1 Hz, 1H), 3.58 (q, *J* = 4.9 Hz, 2H), 1.49 (s, 3H), 1.29 (s, 3H). **^{13}C NMR** (101 MHz, DMSO-*d*$_6$) δ 163.17, 150.72, 138.80, 114.73, 113.57, 91.47, 86.70, 83.92, 81.04, 61.77, 56.38, 27.50, 25.63. **HRMS** (ESI$^+$) m/z calcd for $C_{13}H_{19}N_2O_7^+$ [M + H] $^+$: 315.1187, found: 315.1184.

5-Azidomethyl-2',3'-O-isopropylideneuridine (21)(*65*)

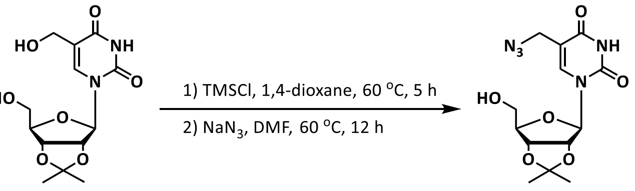

20 21

5-Hydroxymethyl-2',3'-O-isopropylideneuridine **20** (1.26 g, 4 mmol) was dissolved in anhydrous1,4-dioxane (60 mL), and then trimethylsilyl chloride (2.16 g, 20 mmol) was added. After stirring at 60 °C for 5 h, the mixture was cooled to room temperature, and anhydrous acetone (50 mL) was added. The mixture was stirred for further1.5 h and then concentrated under reduced pressure. The resulting residue was redissolved in anhydrous DMF (50 mL), and NaN_3 (650 mg, 10 mmol) was added slowly. The resulting mixture was stirred for 12 h at 60 °C. After completion, the solvent was removed under reduced pressure and the residue was purified by column chromatography (MeOH/CH_2Cl_2=1/20) to give 5-azidomethyl-2',3'-O-isopropylideneuridine **21** (840 mg, 62%) as a white solid. **^{1}H NMR** (400 MHz, $CDCl_3$) δ 9.21 (br, 1H), 7.53 (s, 1H), 5.66 (d, *J* = 2.9 Hz, 1H), 4.99 (ddd, *J* = 23.1, 6.4, 3.1 Hz, 2H), 4.32 (q, *J* = 3.1 Hz, 1H), 4.15 (s, 2H), 3.94 (dd, *J* = 12.0, 2.6 Hz, 1H), 3.82 (d, *J* = 12.4 Hz, 1H), 2.87 (br, 1H), 1.59 (s, 3H), 1.37 (s, 3H). **^{13}C NMR** (101 MHz, $CDCl_3$) δ 162.38, 150.01, 140.90, 114.50, 109.88, 95.69, 86.94, 83.82, 80.33, 62.63, 47.03, 27.24, 25.25. **HRMS** (ESI$^+$) m/z calcd for $C_{13}H_{18}N_5O_6^+$ [M + H] $^+$: 340.1252, found: 340.1255.

5-Azidomethyluridine (22)(*65*)

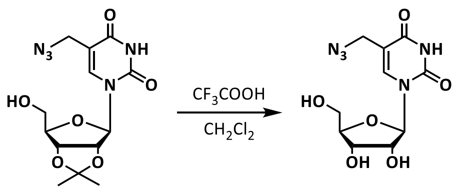

21 22

5-Azidomethyl-2',3'-O-isopropylideneuridine **21** (848 mg, 2.5 mmol) was dissolved in anhydrous CH_2Cl_2 (10 mL), and trifluoroacetic acid (20 mL) was added at 0 °C. After stirring for 5 h at room temperature, the solvent was removed under reduced pressure. The residue was purified by column chromatography (MeOH/CH_2Cl_2=1/10) to give 5-azidomethyluridine **22** (680 mg, 91% yield) as a white solid. **^{1}H NMR** (400 MHz, DMSO-*d*$_6$) δ 11.57 (s, 1H), 8.08 (s, 1H), 5.77 (d, *J* = 5.1 Hz, 1H), 5.40 (br, 1H), 5.12 (br, 2H), 4.04 (d, *J* = 7.1 Hz, 3H), 3.97 (dd, *J* = 6.2, 3.1 Hz, 1H), 3.85 (q, *J* = 3.6 Hz, 1H), 3.68-3.54 (m, 2H). **^{13}C NMR** (101 MHz, DMSO-*d*$_6$) δ 163.32, 150.93, 140.54, 108.75, 88.34, 85.28, 73.99, 70.12, 61.20, 47.39. **HRMS** (ESI$^+$) m/z calcd for $C_{10}H_{14}N_5O_6^+$ [M + H] $^+$: 300.0939, found: 300.0935.

5-RIBOTAC-U (23)

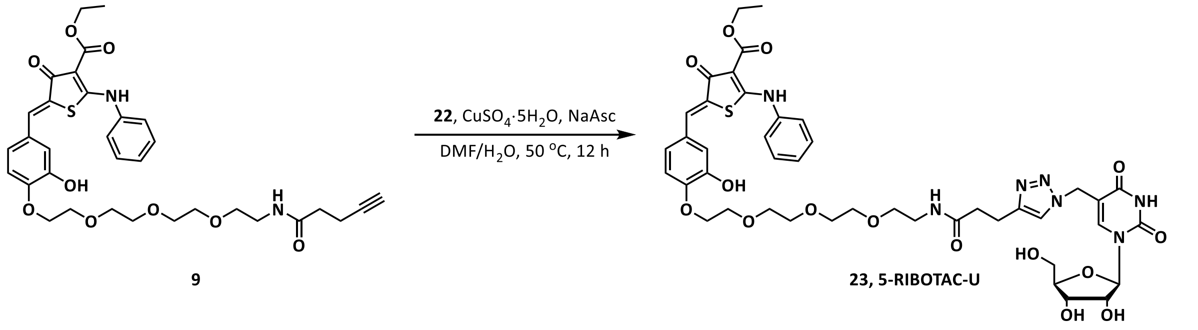

To a solution of 5-azidomethyluridine **22** (299 mg, 1.0 mmol) and compound **22** (638 mg, 1.0 mmol) in DMF (20 mL), sodium ascorbate (990 mg, 5.0 mmol) in water (5 mL) and copper (II) sulfate pentahydrate (249 mg, 1.0 mmol) in water (5 mL) were added respectively. Thereafter, the reaction mixture was stirred at 50 °C for 12 h. After completion, the solvent was removed under reduced pressure and the residue was purified by silica gel column chromatography (MeOH/CH_2Cl_2=1/10) to afford the desired product **5-RIBOTAC-U 23** (646 mg, 69% yield) as a yellow solid. **^{1}H NMR** (400 MHz, Methanol-*d*$_4$) δ 8.19 (s, 1H), 7.61 (s, 1H), 7.53 (s, 1H), 7.46-7.33 (m, 5H), 6.89 (d, *J* = 5.8 Hz, 2H), 5.77 (d, *J* = 3.7 Hz, 1H), 5.07 (s, 2H), 4.27 (q, *J* = 7.1 Hz, 2H), 4.11-4.04 (m, 4H), 3.91 (dt, *J* = 5.0, 2.7 Hz, 1H), 3.78-3.59 (m, 8H), 3.55 (dt, *J* = 6.2, 1.8 Hz, 2H), 3.53-3.49 (m, 2H), 3.46 (dt, *J* = 6.2, 1.9 Hz, 2H), 3.36 (t, *J* = 5.5 Hz, 2H), 3.25 (s, 1H), 2.83 (t, *J* = 7.3 Hz, 2H), 2.39 (t, *J* = 7.4 Hz, 2H), 1.28 (t, *J* = 7.1 Hz, 3H). **^{13}C NMR** (101 MHz, Methanol-*d*$_4$) δ 183.15, 176.75, 173.23, 165.66, 163.17, 150.73, 148.78, 146.94, 146.23, 141.41, 137.34, 131.69, 129.56, 128.09, 127.05, 124.93, 124.82, 123.50, 122.48, 116.07, 112.93, 108.12, 97.55, 89.57, 84.92, 74.49, 70.14, 70.11, 70.01, 69.75, 69.58, 69.11, 67.87, 60.66, 60.01, 46.23, 38.89, 34.83, 21.12, 13.44. **HRMS** (ESI$^+$) m/z calcd for $C_{43}H_{52}N_7O_{15}S^+$ [M + H] $^+$: 938.3237, found: 938.3197.

Key Resources Table

REAGENT or RESOURCE	SOURCE	IDENTIFIER
**Cell Lines**		
Huh7 cells	ATCC	#Bio-73061
Vero E6 cells	ATCC	#Bio-68338
Huh7-ACE2 cells	This study	N/A
**Antibodies**		
SARS-CoV-2 N Mouse mAb	ABclonal Technology Co., Ltd	#A20142
GAPDH Mouse mAb	Proteintech Group, Inc	#60004-1-Ig
Vinculin Rabbit mAb	ABclonal Technology Co., Ltd	#A2752
SARS-CoV-2 N Rabbit mAb	ABclonal Technology Co., Ltd	#A20021
SARS-CoV-2 N Rabbit mAb	Sino Biological, Inc	#40143-R001
Alexa Fluor-488 Goat Anti-Mouse IgG	Abcam	#ab150113
HRP Goat Anti-rabbit IgG	Sigma Aldrich	#A6154
HRP Goat Anti-mouse IgG	Sigma Aldrich	#A4416
**Virus Strains**		
Wild Type SARS-CoV-2	Dr. Zheng-Li Shi, National Virus Resource Center (NVRC), Wuhan Institute of Virology, Chinese Academy of Sciences, Wuhan, China (*66*)	IVCAS 6.7512
**Critical Commercial Assays**		
The MiniBEST Viral RNA Extraction Kit	TaKaRa	#9766
PrimeScript™ RT reagent Kit with gDNA Eraser	TaKaRa	#RR047A
TB Green® Premix Ex Taq™ II (Tli RNaseH Plus)	TaKaRa	#RR820B
Hoechst 33258	Beyotime Biotechnology	#C1011
Trizol	Invitrogen	#15596026
Lipofectamine RNAiMAX	Thermo Fisher Scientific	#13778075
Hifair® V one-step RT-gDNA digestion SuperMix for qPCR	YEASEN	#11142ES60
Hieff® qPCR SYBR Green Master Mix	YEASEN	#11201ES08
BeyoECL Star	Beyotime Biotechnology	#P0018AM

Fetal Bovine Serum	Gibco	#10099141C
Dulbecco’s Modification of Eagle’s Medium media	Gibco	#11995065
Eagle’s Minimum Essential Medium	Gibco	#11095080
Penicillin/Streptomycin	Cytiva	#SV30010
Trypsin-EDTA, phenol red	Gibco	#25200056
Cell Counting Kit-8	MedChemExpress	#HY-K0301

General Biological Procedures

Virology Experiments Setting

All virological experiments with SARS-CoV-2 were conducted in a Biosafety Level 3 Laboratory (BSL3) at the National Biosafety Laboratory, Wuhan, within the Chinese Academy of Sciences.

Cell Culture and Virus Propagation

Huh7 cells were maintained in Dulbecco's modified Eagle's medium (DMEM, GIBCO) supplemented with 10% fetal bovine serum (FBS, GIBICO). Vero E6 cells were cultivated in Eagle's Minimum Essential Medium (EMEM) also supplemented with 10% FBS. All cell lines were purchased from ATCC. To produce Huh7-ACE2 cells, Huh7 cells were transduced using pLVX-hACE2-IRES-Puro lentivirus. Post-transduction, cells were selected with a concentration of 2.5 μg/mL puromycin. This was then followed by monoclonal screening, as previously elaborated in references (*51, 59*). SARS-CoV-2 (WIV04, GenBank: MN996528.1, CSTR: 16533.06. IVCAS 6.7512) was propagated in Vero E6 cells. The viral titers were ascertained using the 50% tissue culture infectious dose ($TCID_{50}$) method. Cell viability was evaluated using the Cell Counting Kit-8 (CCK-8, MCE).

Antibody and Reagent

Primary antibodies were purchased from the specified manufacturers: Mouse monoclonal antibody (mAb) against SARS-CoV-2 N (ABclonal, # A20142); Mouse monoclonal antibody (mAb) against GAPDH (Proteintech, 60004-1-Ig); Rabbit monoclonal antibodies (mAbs) against SARS-CoV-2 N (Sino Biological Inc., #40143-R001; ABclonal, #A20021). The fluorescence-labeled secondary antibodies utilized were Alexa Fluor-488 goat anti-mouse IgG (Abcam, #ab150113). Horseradish peroxidase (HRP)-labeled goat anti-rabbit (#A6154) and anti-mouse IgG antibodies (#A4416) were purchased from Sigma Aldrich. The MiniBEST Viral RNA Extraction Kit, PrimeScript RT reagent Kit with gDNA Eraser, and SYBR Green Realtime PCR Master Mix were all purchased from TAKARA. Hoechst 33258 (Beyotime), Trizol (Invitrogen), and Lipofectamine RNAiMAX (Thermo Fisher) were obtained from their respective manufacturers.

CCK-8 Assay for Compound Toxicity

2×10^4 Huh7-ACE2 cells were seeded in a 96-well plate. After 24 hours, the cells were treated with various concentrations of the compound for another 24 hours. Subsequently, 10 μL of CCK-8 solution was added to each well, ensuring no bubbles were formed. The plate was then incubated in a culture chamber for 1-4 hours. Absorbance at 450 nm was measured using an enzyme-linked immunosorbent assay (ELISA) reader.

Cell viability was calculated using the formula: Viability (%) = [(As-Ab) / (Ac-Ab)] × 100%

Inhibition rate was determined by: Inhibition (%) = [(Ac-As) / (Ac-Ab)] × 100%

Where: As represents the absorbance of the experimental well (containing cells, culture medium, CCK-8 solution, and compound solution). Ac denotes the absorbance of the control well (containing cells, culture medium, and CCK-8 solution, but without the compound). Ab signifies the absorbance of the blank well (containing culture medium and CCK-8 solution, but without cells or compound).

Compound Antiviral Assay

Acquisition of Huh7-ACE2 Cells: The pLVX-IRES-PURO-hACE2 expression plasmid encoding human ACE2 was constructed. This plasmid, along with lentiviral packaging vectors psPAX2 and pMD2.G, were co-transfected into HEK293T cells. After 48 hours, the supernatant was harvested to obtain lentiviral particles. The packaged lentivirus was then used to transduce Huh7 cells. Selection was performed with 2 μg/mL puromycin for 3-4 days. Surviving cells were then harvested and subjected to a limiting dilution technique to obtain single cells. Western blotting (WB) was later employed to identify cell lines stably expressing ACE2 (*59*).

Compound Antiviral Experiment: Huh7-ACE2 cells were initially seeded at ~1.5×10^5 in a 24-well plate and, on the subsequent day, ~2.5×10^5 Huh7-ACE2 cells were used. This experimentation began with a 20 mM stock solution of the compound in DMSO. Ideally, following a four-fold dilution pattern, the third concentration should have been 6.25 μM. However, for ease of recognition and consistency in the dilution series, this was adjusted to 6.4 μM. Following the 6.4 μM concentration, the four-fold dilution pattern was continued, resulting in subsequent concentrations of 1.6 μM, 0.4 μM, and the smallest concentration being 0.1 μM. This compound solution, along with DMSO, was incorporated into DMEM complete medium at a 1:200 ratio and mixed thoroughly. The old medium from the Huh7-ACE2 cells was then discarded and, beginning with the lowest concentration, 400 μL of medium containing varied concentrations of the compound was added, with three replicate wells set up for each concentration. One hour after the compound was added, the cells were infected with a viral diluent at an MOI of roughly 0.05 (100 μL/well), with the plate gently shaken every 15 minutes for an hour, totaling four times. The supernatant was then discarded and substituted with 500 μL of medium carrying either DMSO or the specific compound concentrations, after which cells were incubated for an additional 24 hours. When collecting cell supernatant samples, they were inactivated by heating at 65 °C for half an hour. Cells could either be lysed using TRIZOL or with 1 × SDS loading buffer and then heated at 100 °C for 10 minutes. Once inactivated, all sample tubes were placed in a disinfectant-filled container. Following surface disinfection, the container could be safely transported out of the Biosafety Level 3 Laboratory.

Time-of-Addition Experiment with 2'-RIBOTAC-U

For the experiments, **2'-RIBOTAC-U** was maintained at a final concentration of 12.5 μM. In the DMSO Control condition, cells underwent treatment with DMSO for 1 hour before SARS-CoV-2 infection. One hour later, the medium was swapped out and replaced with DMSO-containing medium, with cells cultured for an additional 24 hours prior to collecting samples. For the Full-time condition, cells were treated with **2'-RIBOTAC-U** for 1 hour before the SARS-CoV-2 infection. An hour after the infection, the medium was refreshed and cells were then cultivated in a **2'-RIBOTAC-U**-containing medium for another 24 hours before sample collection. In the Entry procedure, after a 1-hour treatment with **2'-RIBOTAC-U**, cells were infected with SARS-CoV-2. One hour post-infection, the medium, devoid of the compound, was replaced and cells were left to culture for another 24 hours leading up to sampling. Lastly, in the Post-entry condition, cells were prepped with fresh medium, excluding the compound, for 1 hour prior to SARS-CoV-2 infection. An hour after this infection, the medium was replenished with one containing **2'-RIBOTAC-U**, and the cells underwent a 24-hour incubation phase ahead of sampling.

Hamster-Based Antiviral Activity Testing of 2'-RIBOTAC-U

Ten 6-week-old female hamsters (~100 g/hamster) were purchased from Beijing Vetone Leihua and divided into two groups of five. The hamsters were acclimated in A3 IVC cages for 1-2 days. After anesthetizing the hamsters, they were exposed to the virus through nasal drops, with each hamster receiving a dose of 1×10^4 TCID50/50-μL of SARS-CoV-2. Approximately 1 hour post-exposure, the hamsters were again anesthetized and intraperitoneally injected with the **2'-RIBOTAC-U** compound (0.05 mg/μL dissolved in 50% DMSO, 200 mg/kg, ~400 μL per hamster). This treatment was repeated daily. On the fourth day post-exposure, the hamsters were sacrificed to obtain lung and tracheal tissues, which were placed in pre-weighed tubes containing either DMEM for tissue grinding or 4% PFA fixative. The tubes were re-weighed. The fixed samples were kept in the dark at room temperature in an Animal Biosafety Level

3 Laboratory (ABSL-3) for more than 10 days. These samples were later used for immunohistochemistry and pathological analyses. The tissue grinding tubes were placed in pre-chilled bio-safe tissue grinding rotors, surface sterilized, and loaded into a tissue grinder to obtain tissue homogenates. After inactivation, they were used for subsequent RNA extraction and WB detection (refer to subsequent sections for RNA extraction and WB sample preparation).

RNA Preparation and Reverse Transcription

Cell or tissue homogenate samples were mixed proportionally with either Trizol or Trizol LS (Life Technologies). After complete lysis, the mixture was transferred to centrifuge tubes and removed from the Biosafety Level 3 Laboratory. RNA was extracted as per standard procedures, and its concentration was measured using a Nanodrop instrument. 1μg of RNA was reverse-transcribed using the PrimeScript™ RT reagent Kit with gDNA Eraser (TAKARA) to obtain cDNA.

For the cell supernatant samples, they were heat-inactivated at 65 °C for 30 minutes. Upon removal from the Biosafety Level 3 Laboratory, RNA was extracted using the Micro Viral RNA Extraction Kit (TAKARA 9766). From this, 5μL samples were reverse-transcribed using the PrimeScript™ RT reagent Kit with gDNA Eraser (TAKARA) to produce cDNA.

qPCR Analysis

Dye-based quantification was performed using the SYBR® Premix Ex Taq II (Tli RNaseH Plus) kit (TAKARA). The template cDNA used was 1-2 μL per 20μL reaction mix, with both forward and reverse primers having a final concentration of 0.4 μM (refer to Table 1 for details). The amplification protocol was set as follows: 95 °C for 30 s, followed by 40 cycles of 95 °C for 5 s, 62 °C for 15 s, and 72 °C for 45 s, with signal collection during the 72 °C elongation phase. Target mRNA levels were relatively quantified using the comparative CT method (2^-ΔΔCT method): ΔΔCT = Treatment Group (Ct Target gene – Ct GAPDH) – Control Group (Ct Target gene – Ct GAPDH). Here, GAPDH was used as the internal reference gene. For determining the viral RNA copy number in the cells, standard curves were established using the plasmids containing the indicated target gene cDNA (ranging from 10^2 to 10^8 copies/μL) for absolute quantification.

Immunofluorescence

After treating with different compounds, the infected samples in the 24-well plates had their supernatants discarded. The entire plate was then fixed at room temperature for 30 minutes in containers filled with 4% paraformaldehyde. Cells were washed three times with PBS and permeabilized with 0.5% Triton X-100 for 10 minutes. Afterward, cells were blocked using 5% BSA at 37 °C for an hour. Primary anti-SARS-CoV-2 N monoclonal antibody (ABclonal, #A20142), diluted 1:500 in PBS, was added and incubated at 4 °C overnight. After washing three times with PBS (10 minutes each time), a secondary FITC-conjugated sheep anti-mouse antibody, diluted 1:500 in PBS, was added and incubated at 37 °C for 1 hour. Following three more PBS washes (10 minutes each), cell nuclei were stained with Hoechst for 5-10 minutes. After two PBS washes, images were captured under a fluorescence microscope (OLYMPUS IX53) and later analyzed using the Image J software.

Western Blot Analysis

After rinsing cell samples with PBS, they were lysed with 1 × SDS sample buffer and thoroughly mixed. This mixture was then transferred to centrifuge tubes. Tracheal homogenate samples, which had a ratio of 0.1 g homogenized in 1 mL DMEM, had 200 μL combined with 5 × SDS sample buffer. Lung tissue homogenate samples, due to their viscosity and having a similar initial ratio, were diluted 5-10 times with DMEM and mixed with 5 × SDS sample buffer. These Western blot samples underwent heat treatment at 100 °C for 10 minutes in a Biosafety Level 3 Laboratory. Prior to SDS-PAGE, these samples were reheated to 100 °C for 5 minutes, centrifuged,

and the resultant supernatant underwent SDS-PAGE, membrane transfer, and a 5% BSA block. For the primary antibody incubation, target antibodies in TBST were introduced to the membranes, left overnight at 4 °C, and subsequently washed three times with TBST. The membranes were then exposed to secondary antibodies at 37 °C for an hour and washed three times with TBST. Finally, ECL substrate was applied to the membranes, and protein expression was visualized using a chemiluminescence detection system.

Lung Tissue Section Preparation

Hamster lung tissues were fixed in 4% PFA for 10 days, and due to Biosafety Level 3 Laboratory protocols, remained in the lab for that duration. Post-fixation, tissues were put into a dehydration container inside a fume hood. The tissues were then dehydrated using a gradient alcohol regimen in a DIPATH Donatello dehydration machine, progressing from 75% alcohol to Paraffin III. These tissues, now soaked in paraffin, were embedded using a Wuhan Junjie Electronics JB-P5 machine: melted paraffin was poured into molds, tissues from the dehydration container were added, labels attached, and samples cooled at -20 °C. After solidification, paraffin blocks were trimmed and then sectioned to 4 μm thickness using a Leica RM2016 microtome. These sections were floated on a 40 °C water bath in a KD-P slide warmer to smooth out, then collected on glass slides and oven-dried at 60 °C. For deparaffinization, sections underwent treatment with eco-friendly solutions from Seville Biotechnology, followed by absolute ethanol washes and a final rinse in distilled water. After this process, sections were primed for H&E staining or immunohistochemistry.

Lung Tissue Section Immunohistochemistry

Sections underwent antigen retrieval using citric acid antigen retrieval solution from Seville Biotechnology with a pH of 6.0, microwaved at varying heats, ensuring minimal buffer evaporation. After cooling naturally, the slides were rinsed three times in PBS. Notably, the retrieval solution and conditions depend on the tissue type. To block endogenous peroxidase, sections were exposed to a 3% hydrogen peroxide solution in darkness and room temperature for 25 minutes, followed by PBS washes. A 3% BSA solution was subsequently applied for serum blocking. Excess solution was removed before incubating the sections with a primary antibody from ABclonal, diluted in PBS, and left in a humidified chamber at 4°C overnight. After PBS washes, the sections were treated with a HRP-labeled secondary antibody from Servicebio. DAB substrate was used for staining, which was closely monitored for a brown-yellow hue indicating positive staining and stopped with tap water. The nuclei were counterstained with hematoxylin, differentiated, and blued using specific solutions, followed by rinses. Sections underwent a specific dehydration series, culminating in an air-drying phase and neutral resin mounting. Slides were then scanned with the PANNORAMIC system and observed with the CaseViewer2.4 software. Lastly, the blue hematoxylin-stained nuclei contrasted with the brown-yellow positive DAB staining for result interpretation.

Lung Tissue Section H&E Staining

Slides underwent staining with Harris hematoxylin for a duration between 3 to 8 minutes and were rinsed with tap water. A brief differentiation was achieved using 1% hydrochloric acid in alcohol, and after another rinse, they were blued using 0.6% ammonia water, concluding with a comprehensive rinse under running water. For cytoplasmic staining, the sections were immersed in eosin stain for a span of 1 to 3 minutes. Following this, they were sequentially dehydrated via a regimen involving 75% and 85% alcohol, two rounds of absolute ethanol, butanol, and xylene. Once dehydrated, they were allowed a brief air-drying period before being mounted with neutral resin. To analyze the results, the entirety of the slides was scanned using the PANNORAMIC DESK/MIDI/250/1000 system and examined via the CaseViewer2.4 software. During the assessment, key pathological changes like inflammation, congestion, stasis, hemorrhage, edema, and degeneration were identified. For clearer understanding, representative pathological regions were captured in images, with arrows marking specific lesions.

Table S1. List of DNA primers.

Name	Forward primer sequence (5'-3')	Reverse primer sequence (5'-3')
**Primers for mRNA analysis**		
GAPDH	ACCACAGTCCATGCCATCAC	TCCACCACCCTGTTGCTGTA
N1	CGAATTCGTGGTGGTGACGG	TGCGGGTGCCAATGTGATCT
N2	GGGGAACTTCTCCTGCTAGAAT	CAGACATTTTGCTCTCAAGCTG
ORF1ab	CCCTGTGGGTTTTACACTTAA	ACGATTGTGCATCAGCTGA

Data S1. NMR Spectra

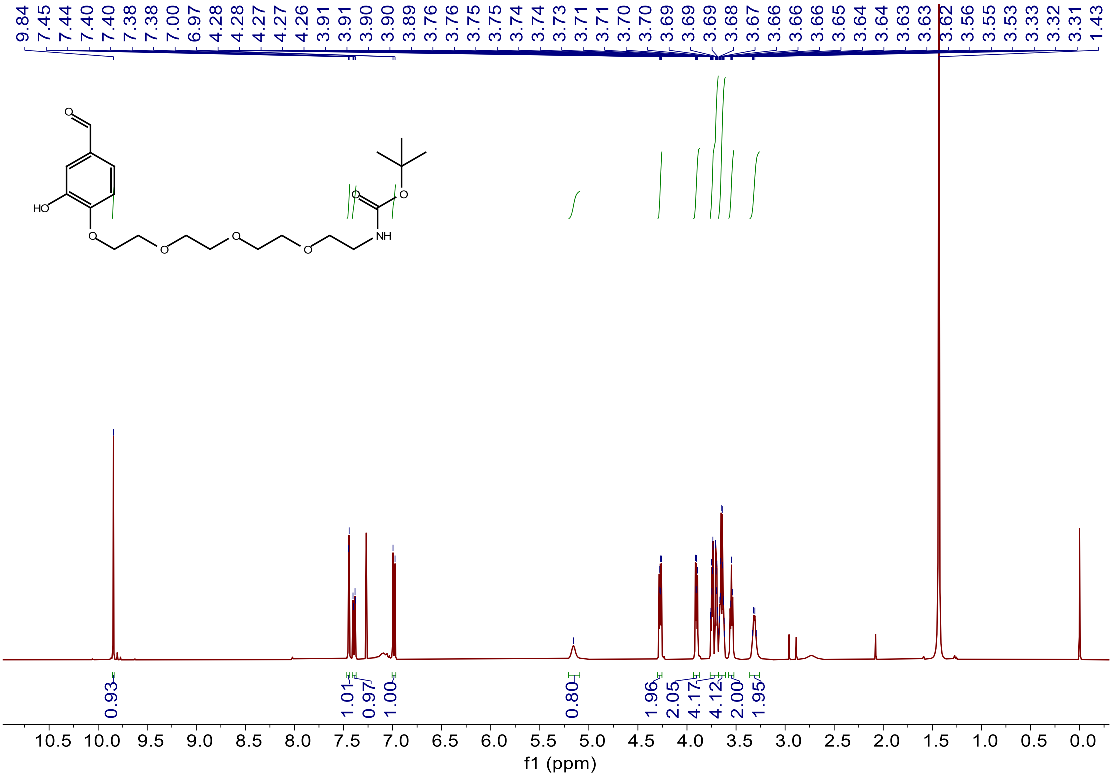

^{1}H NMR spectrum of compound **3** in $CDCl_3$

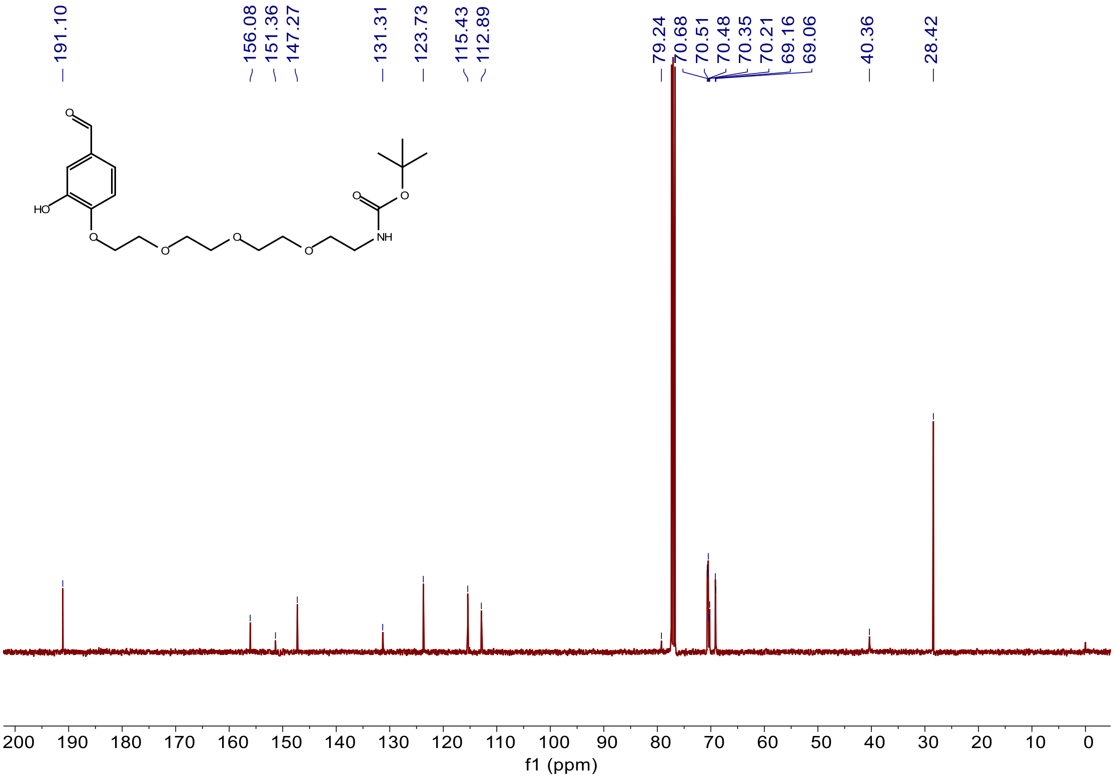

^{13}C NMR spectrum of compound **3** in $CDCl_3$

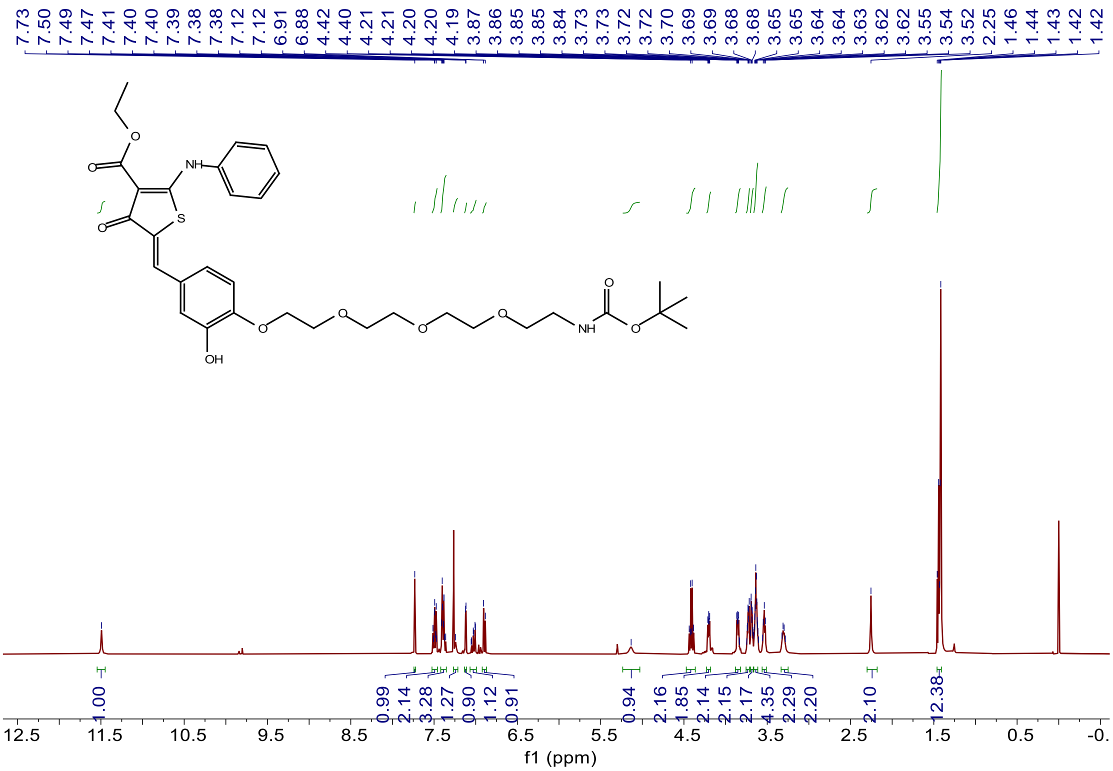

^{1}**H NMR** spectrum of compound **5** in $CDCl_3$

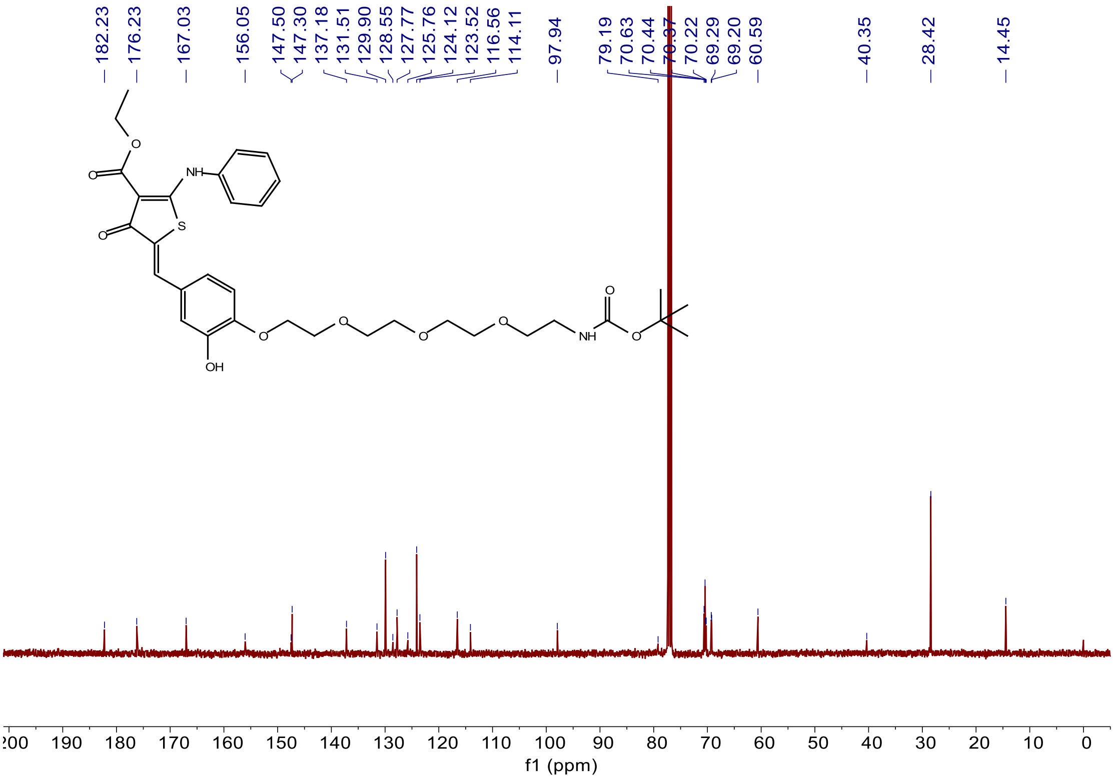

^{13}**C NMR** spectrum of compound **5** in $CDCl_3$

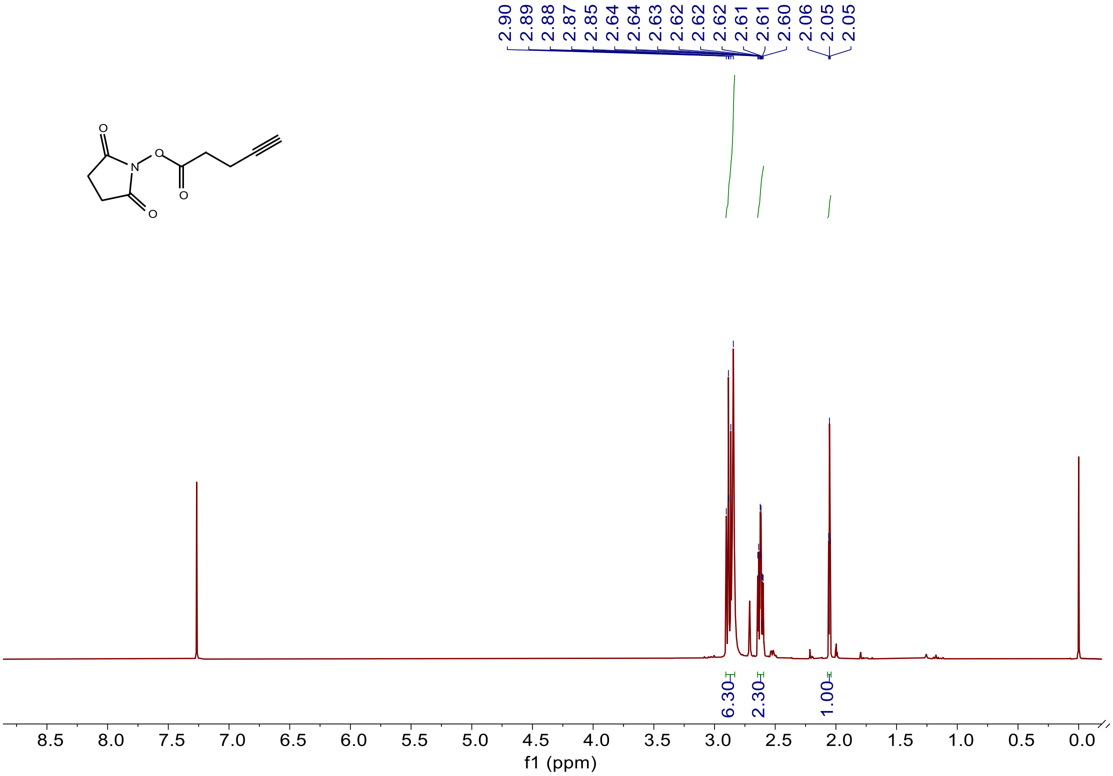

^{1}H NMR spectrum of compound **8** in $CDCl_3$

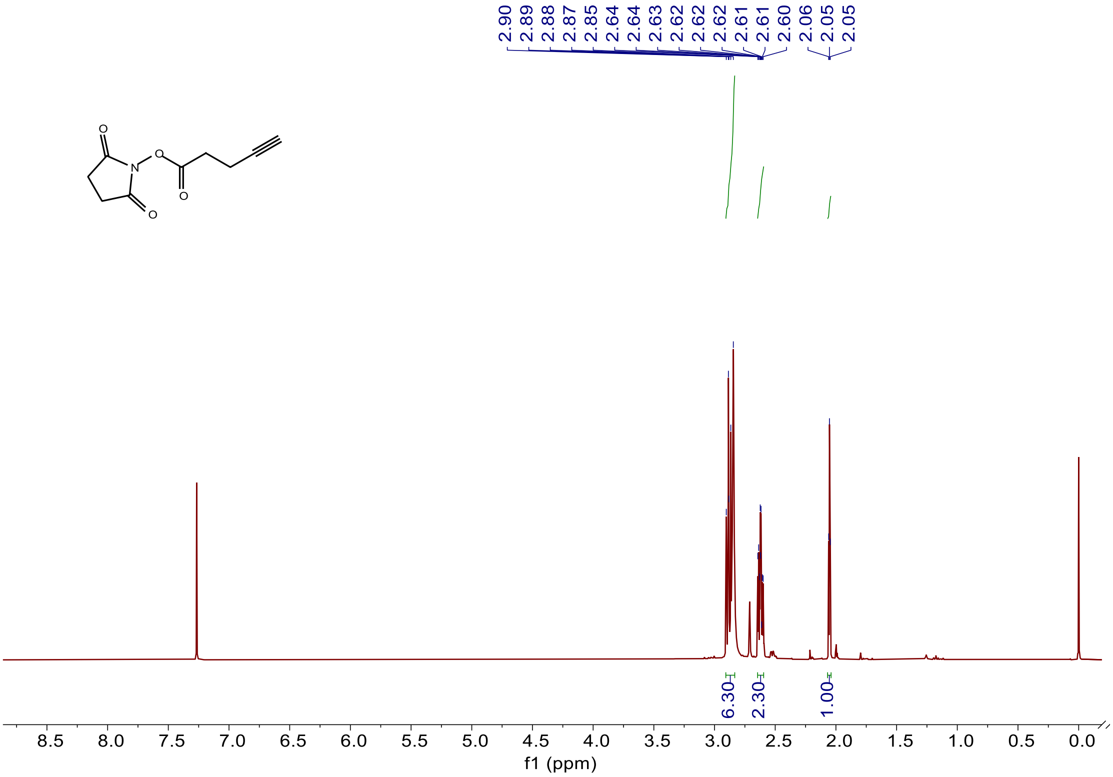

^{13}C NMR spectrum of compound **8** in $CDCl_3$

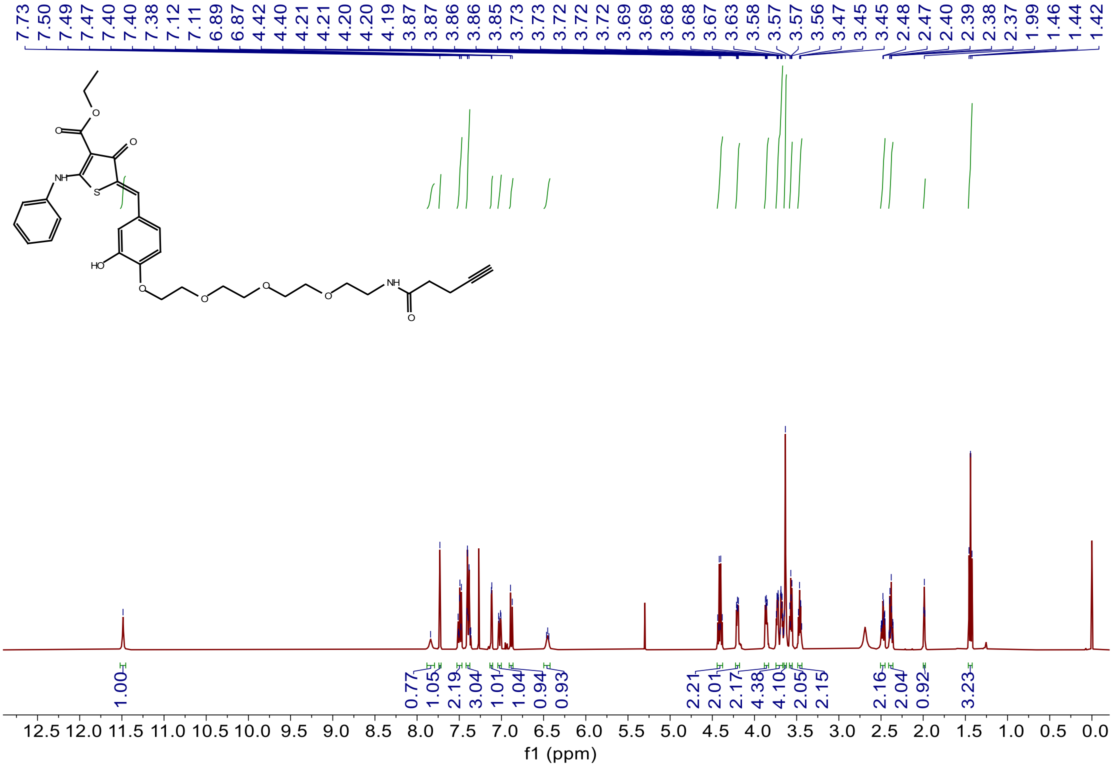

^{1}H NMR spectrum of compound **9** in $CDCl_3$

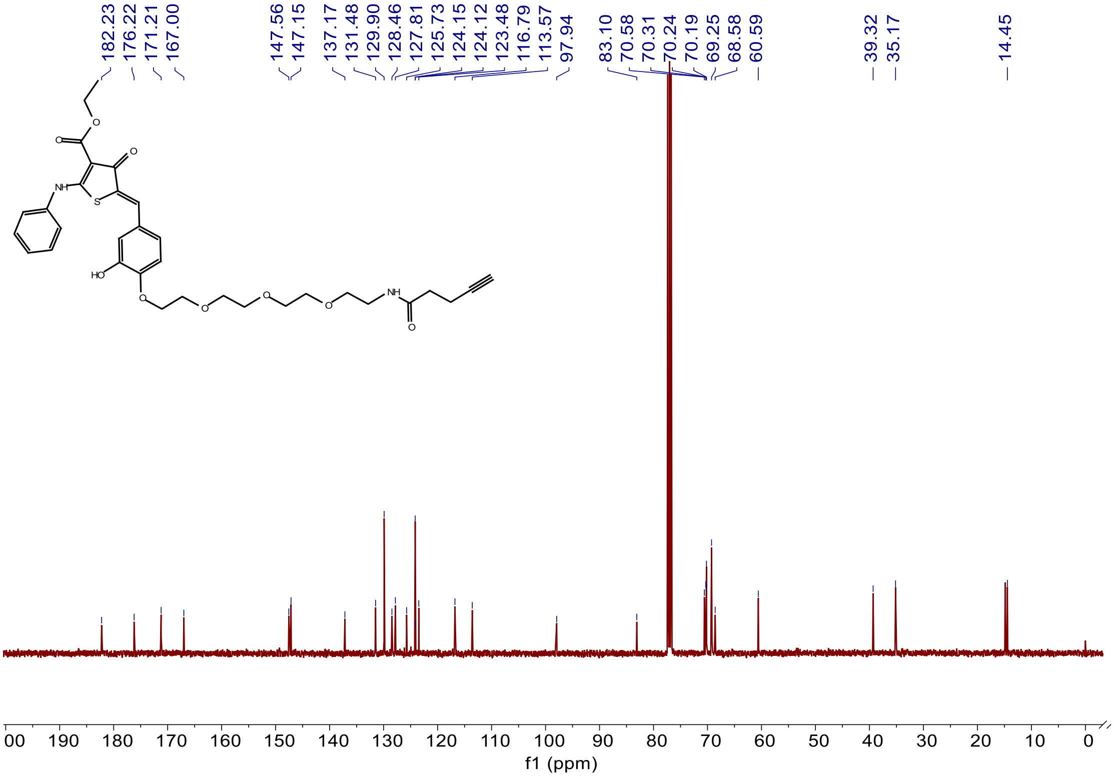

^{13}C NMR spectrum of compound **9** in $CDCl_3$

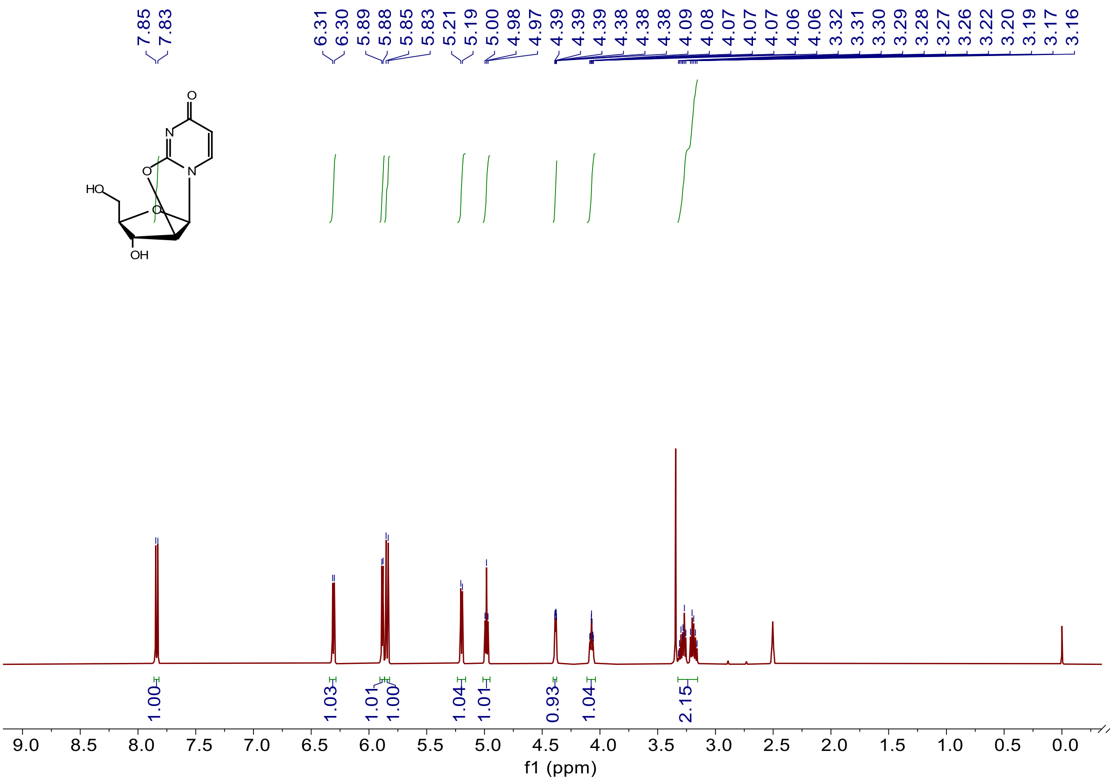

^{1}H NMR spectrum of compound **11** in DMSO-d_6

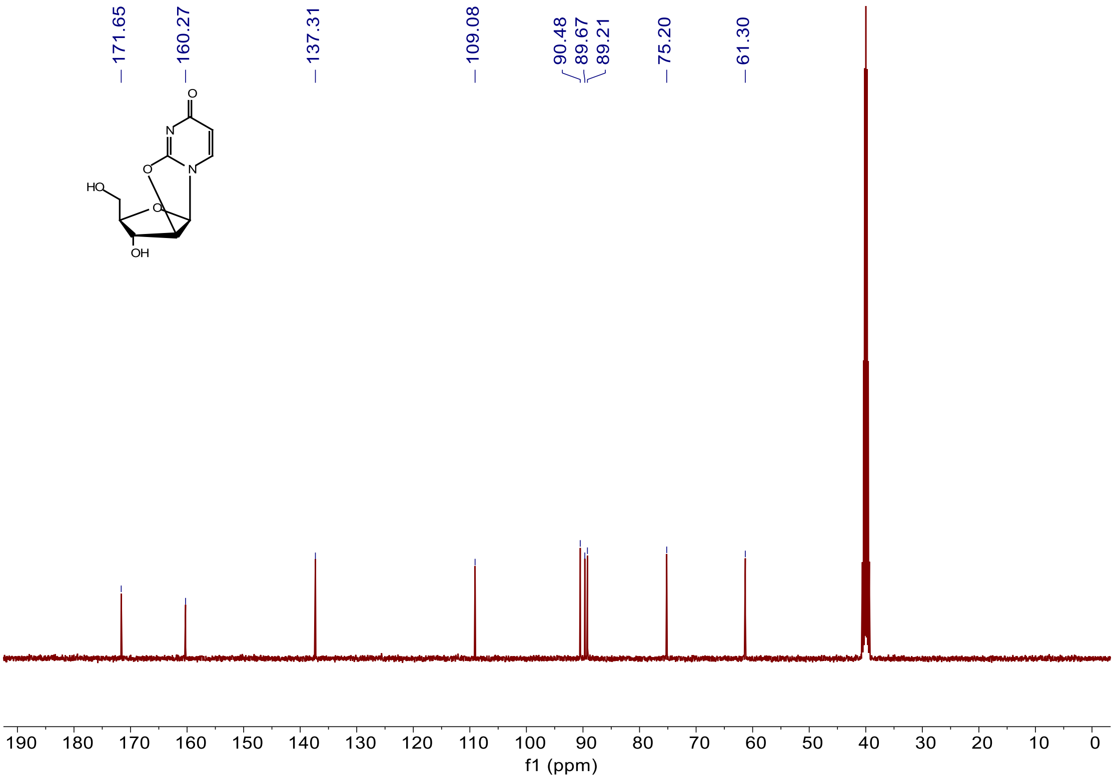

^{13}C NMR spectrum of compound **11** in DMSO-d_6

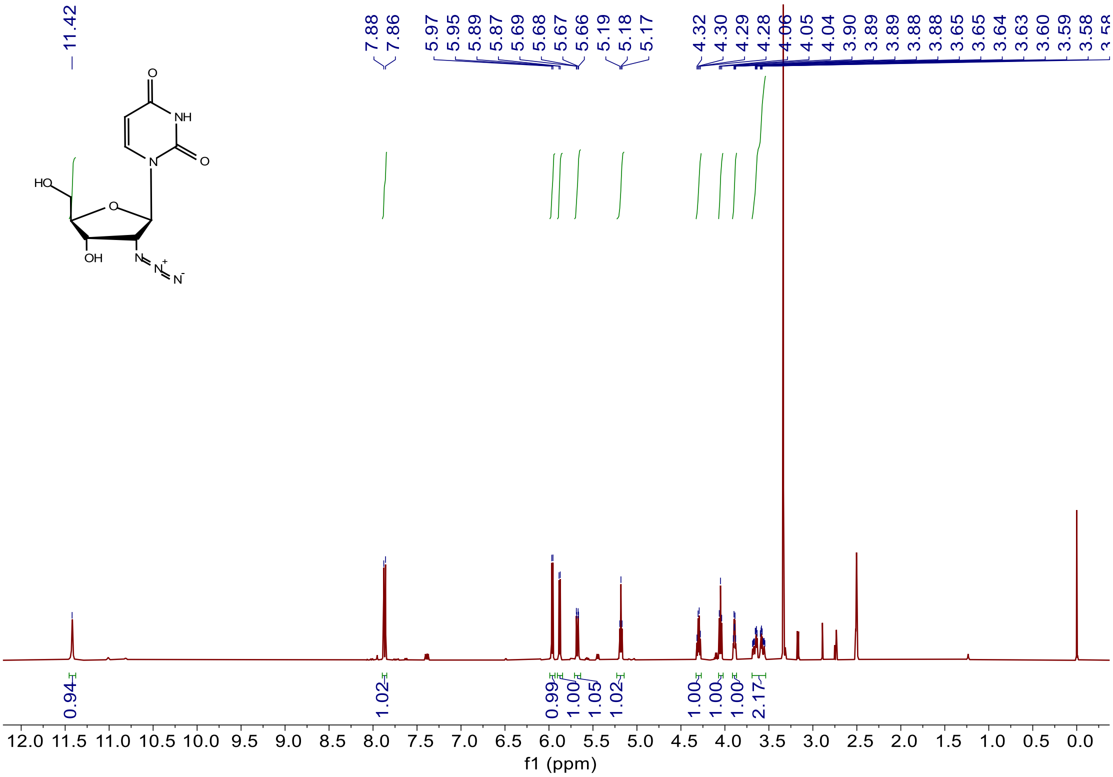

^{1}H NMR spectrum of compound **12** in DMSO-d_6

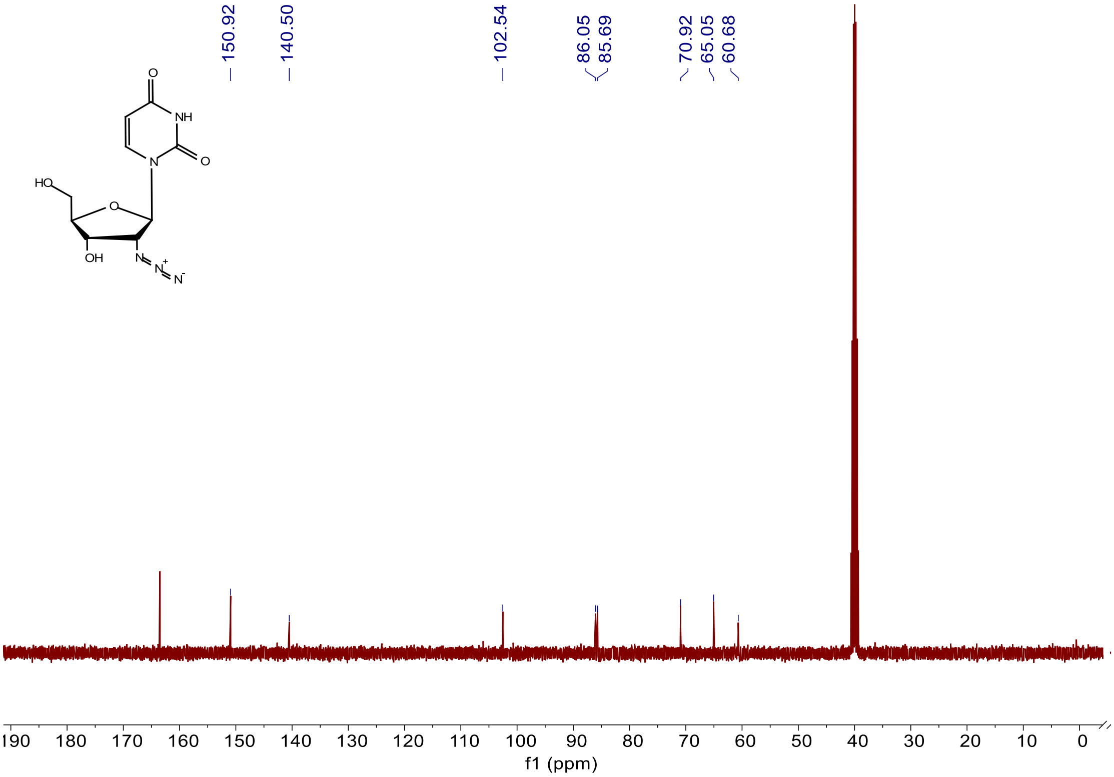

^{13}C NMR spectrum of compound **12** in DMSO-d_6

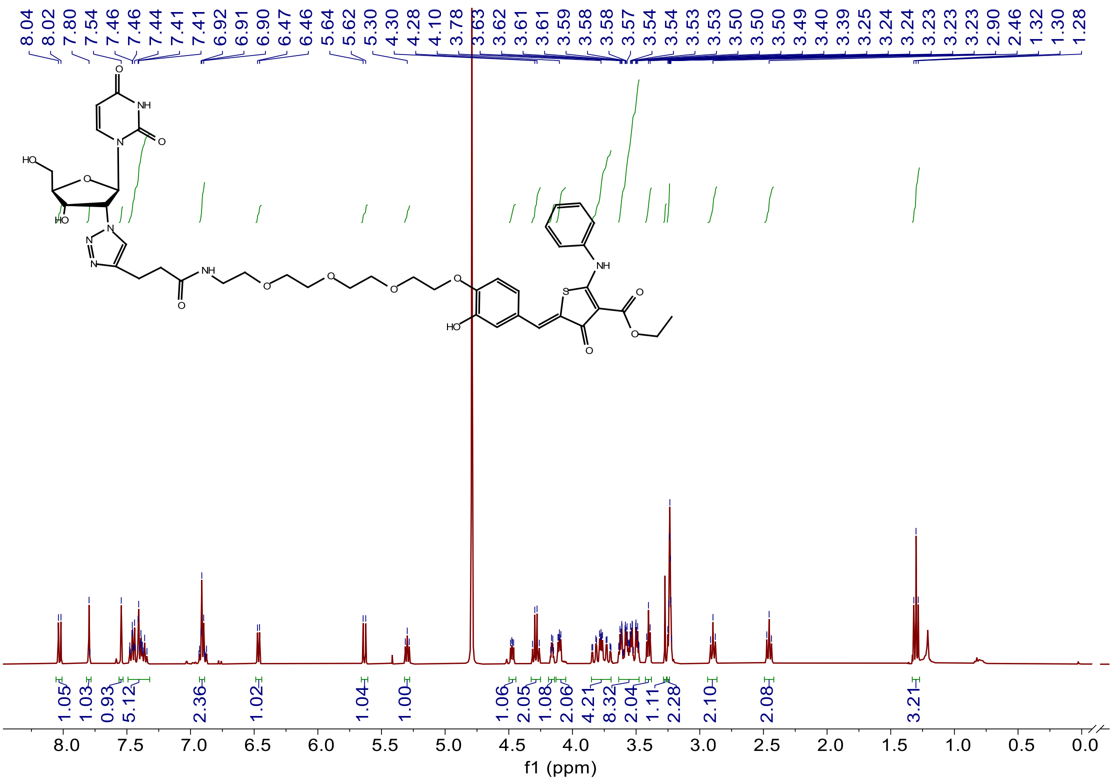

^{1}H NMR spectrum of **2'-RIBOTAC-U (13)** in methanol-d_4

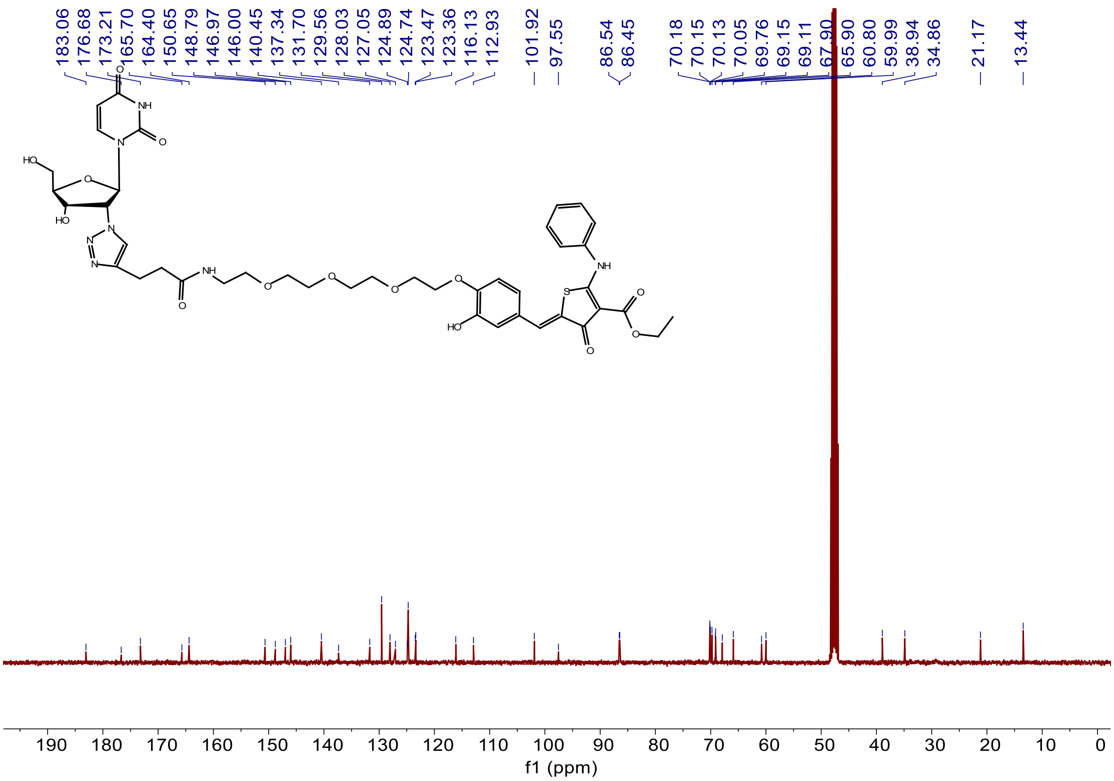

^{13}C NMR spectrum of **2'-RIBOTAC-U (13)** in methanol-d_4

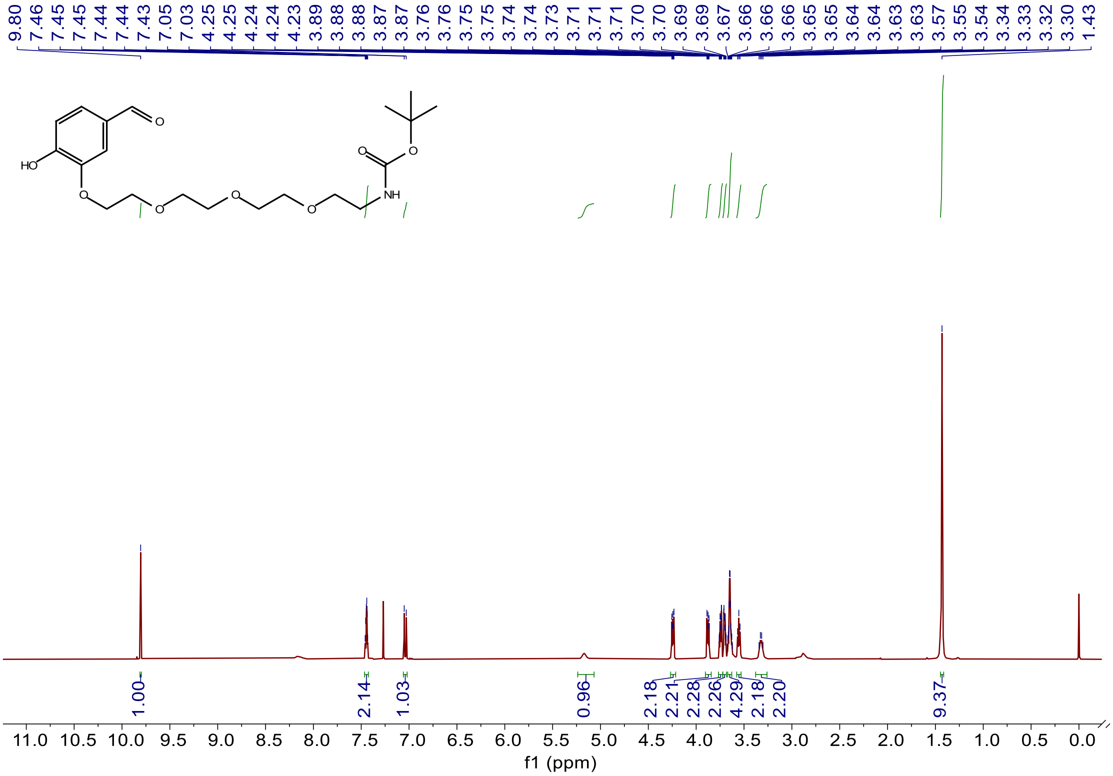

^{1}H NMR spectrum of compound **14** in $CDCl_3$

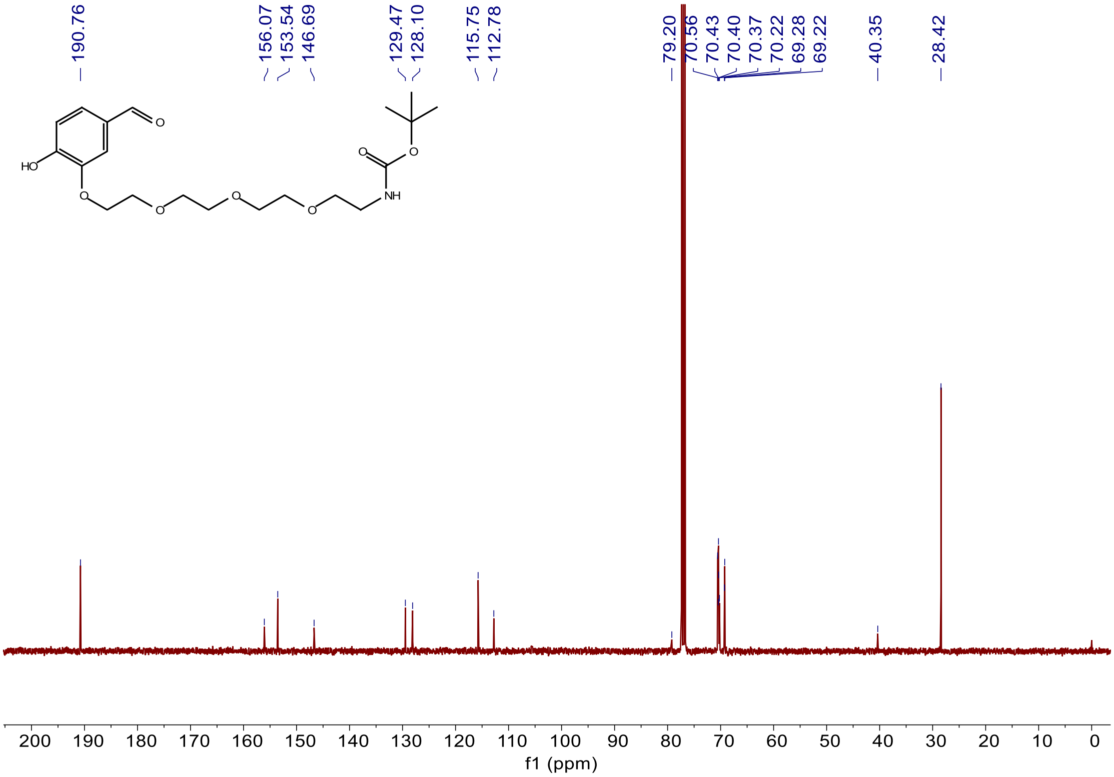

^{13}C NMR spectrum of compound **14** in $CDCl_3$

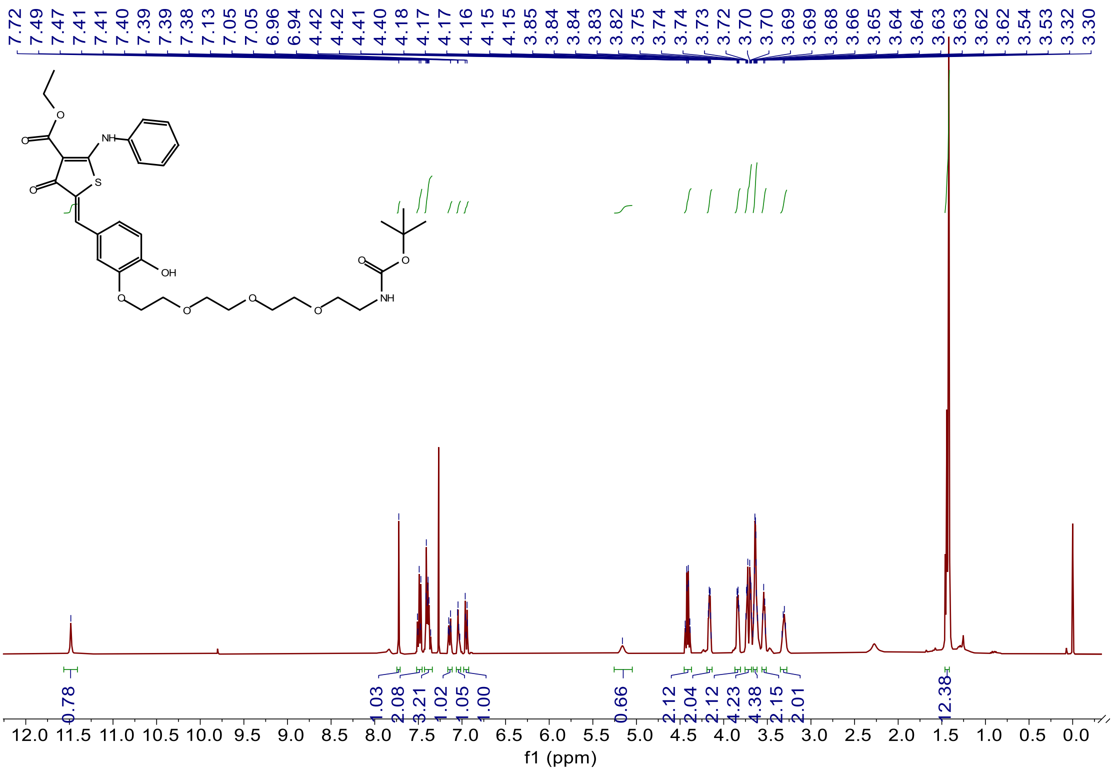

¹H NMR spectrum of compound **15** in $CDCl_3$

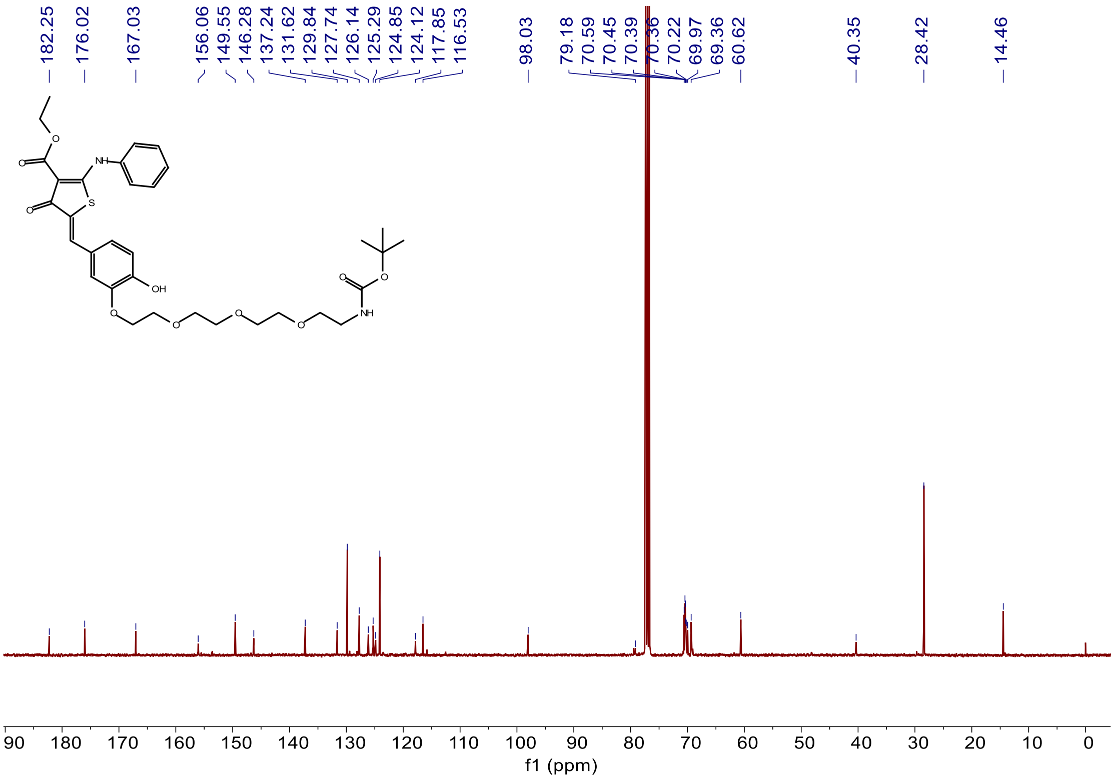

¹³C NMR spectrum of compound **15** in $CDCl_3$

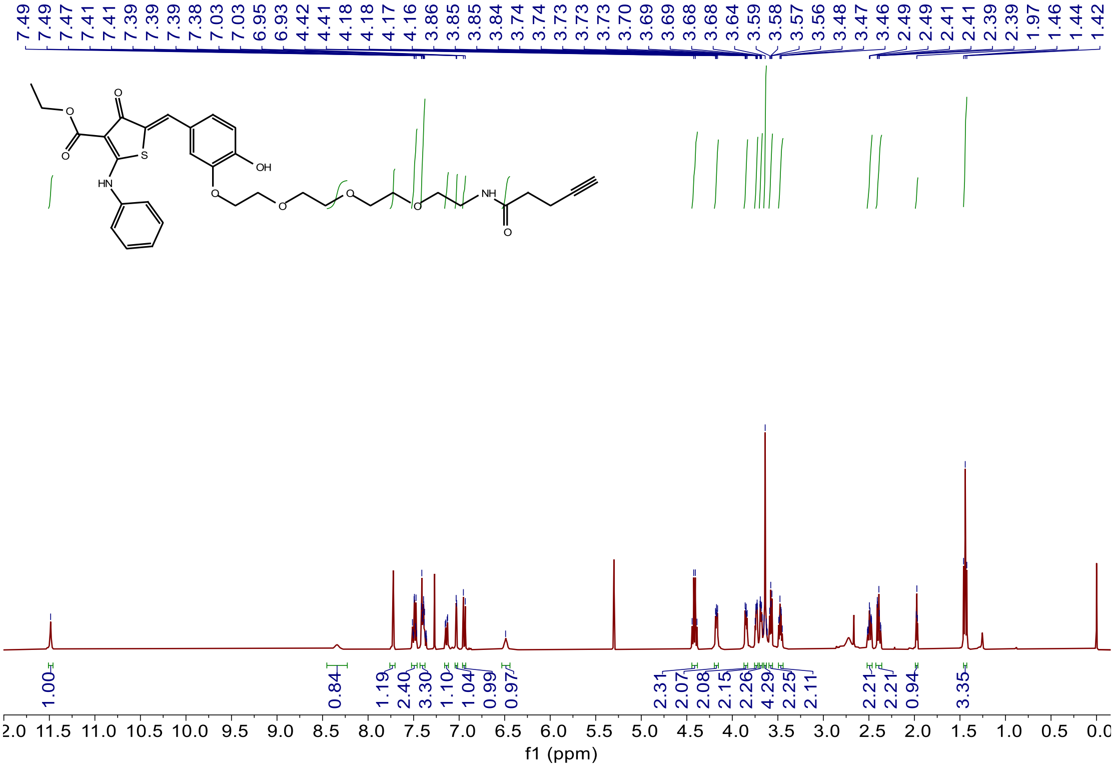

^{1}H NMR spectrum of compound **17** in $CDCl_3$

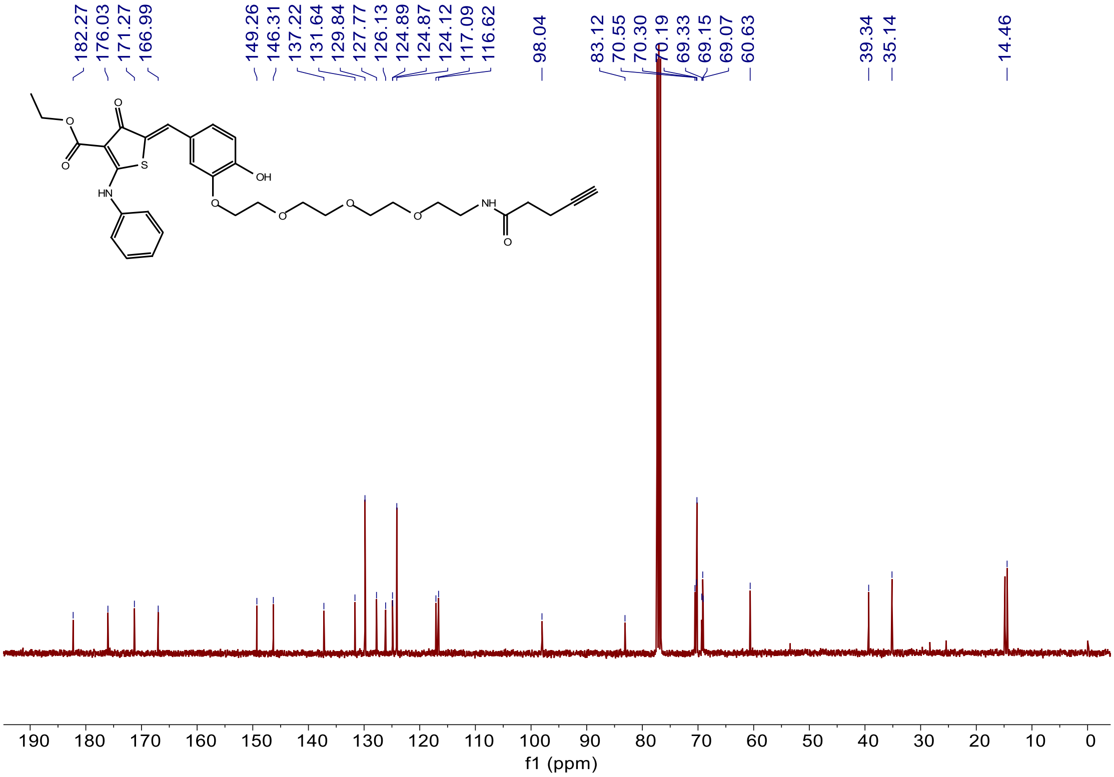

^{13}C NMR spectrum of compound **17** in $CDCl_3$

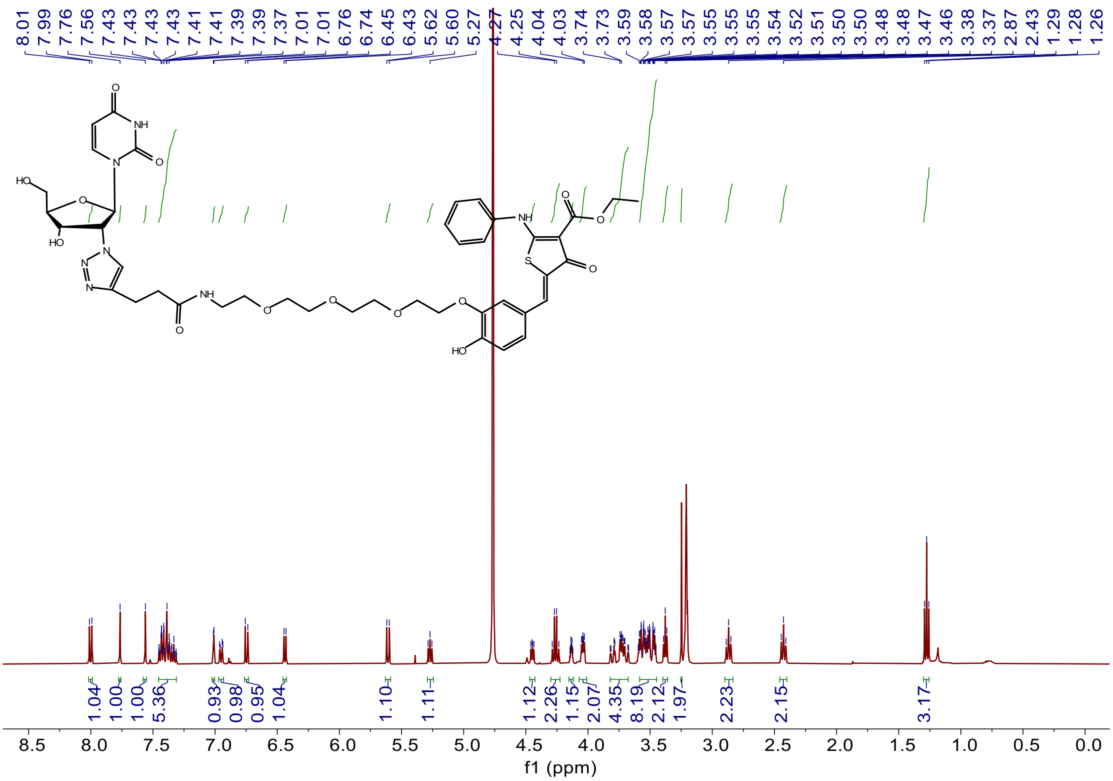

^{1}H NMR spectrum of **2'-mutRIBOTAC-U (18)** in methanol-d_4

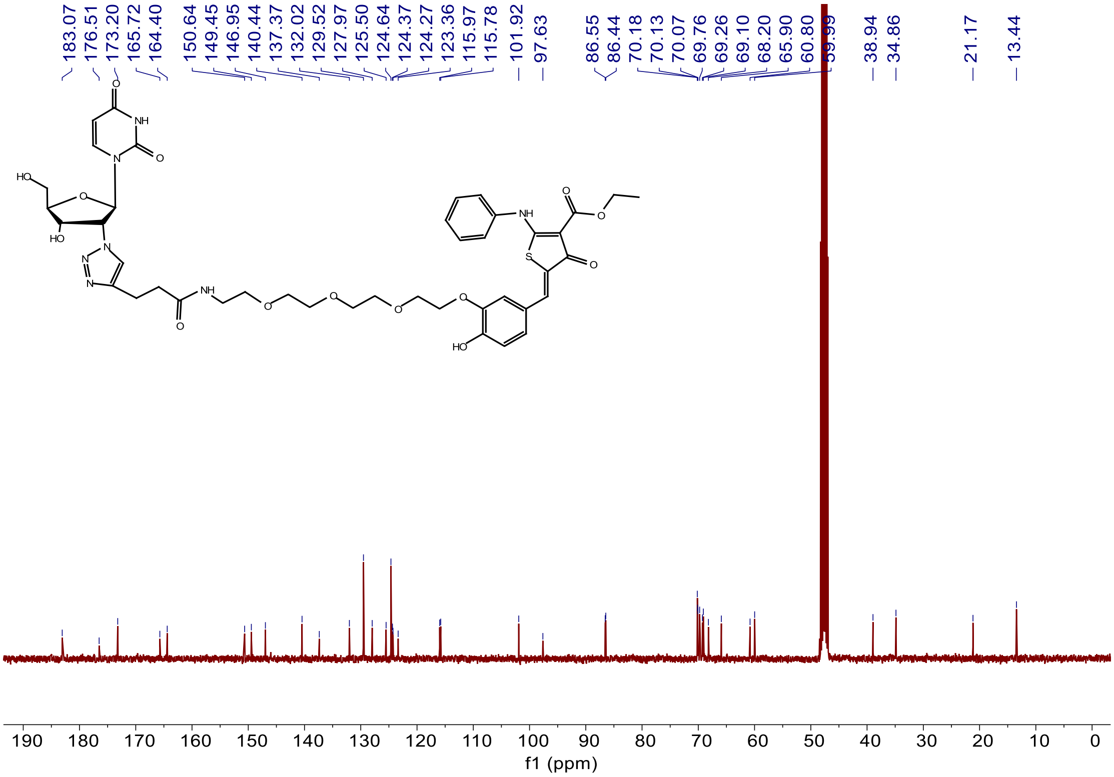

^{13}C NMR spectrum of **2'-mutRIBOTAC-U (18)** in methanol-d_4

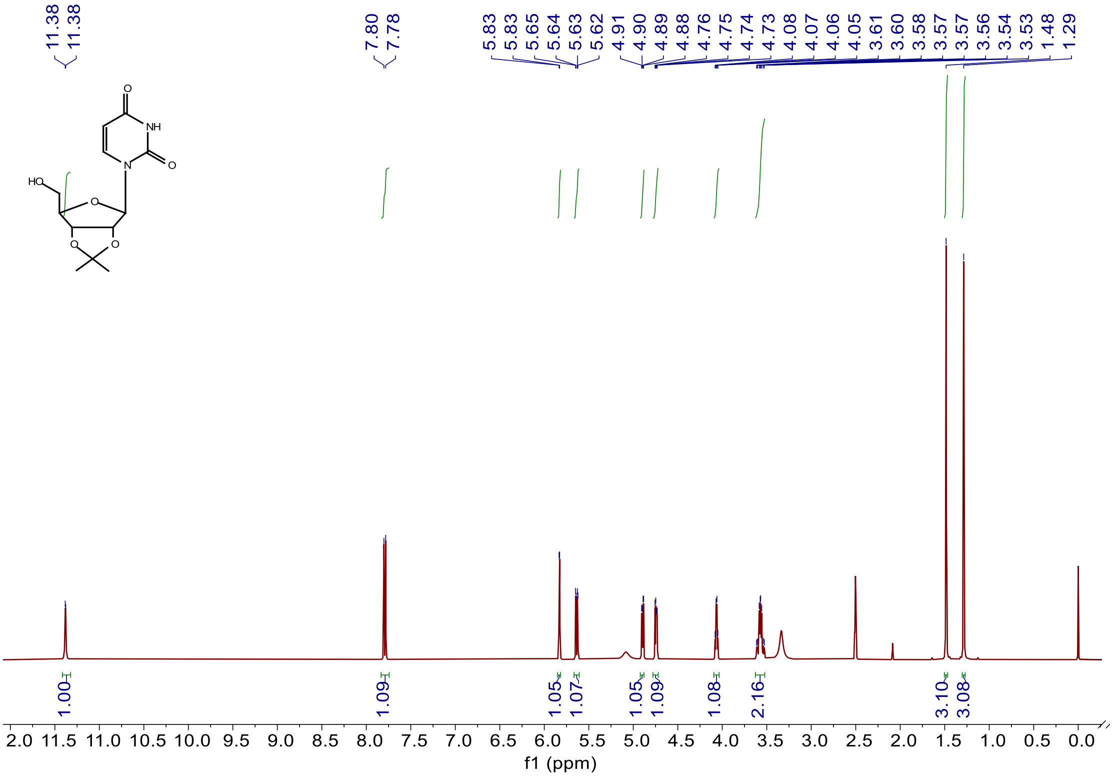

^{1}H NMR spectrum of compound **19** in DMSO-d_6

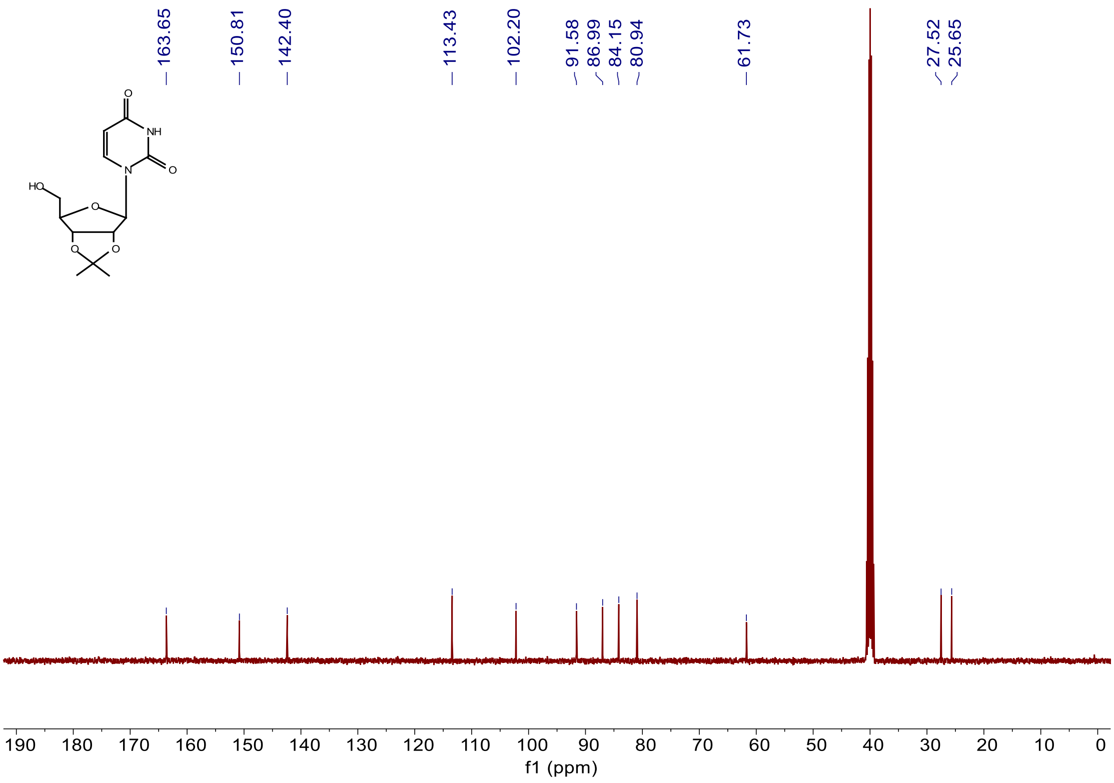

^{13}C NMR spectrum of compound **19** in DMSO-d_6

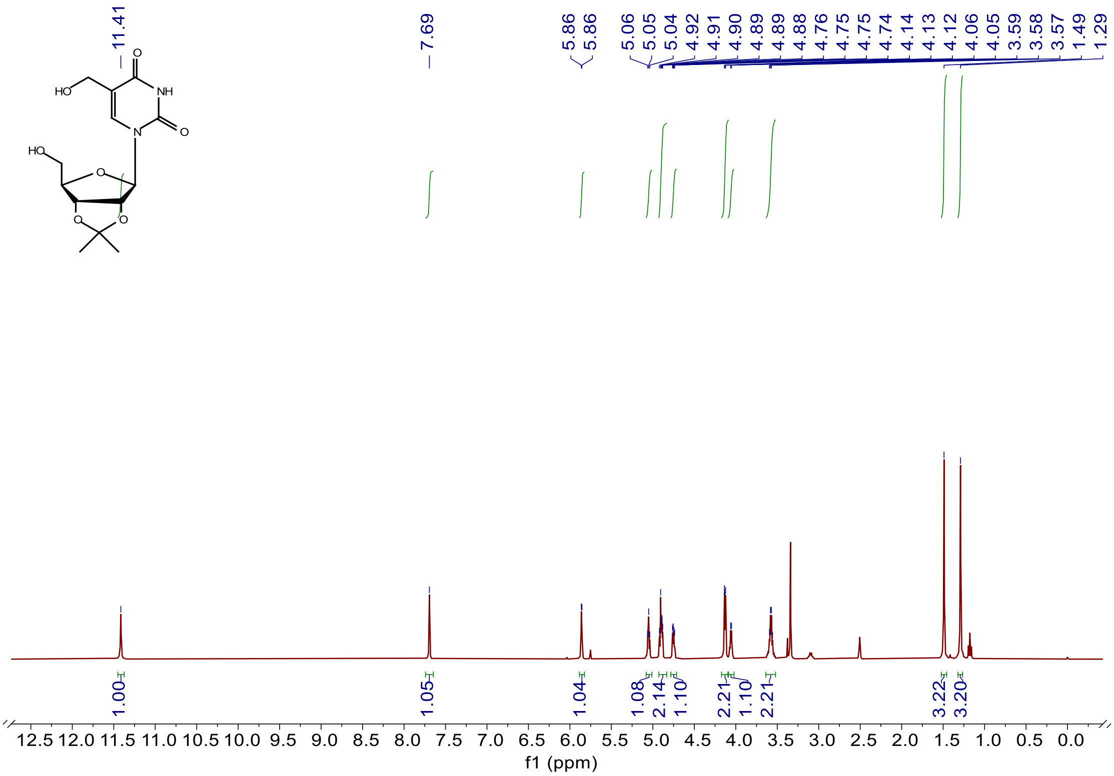

^{1}H NMR spectrum of compound **20** in DMSO-d_6

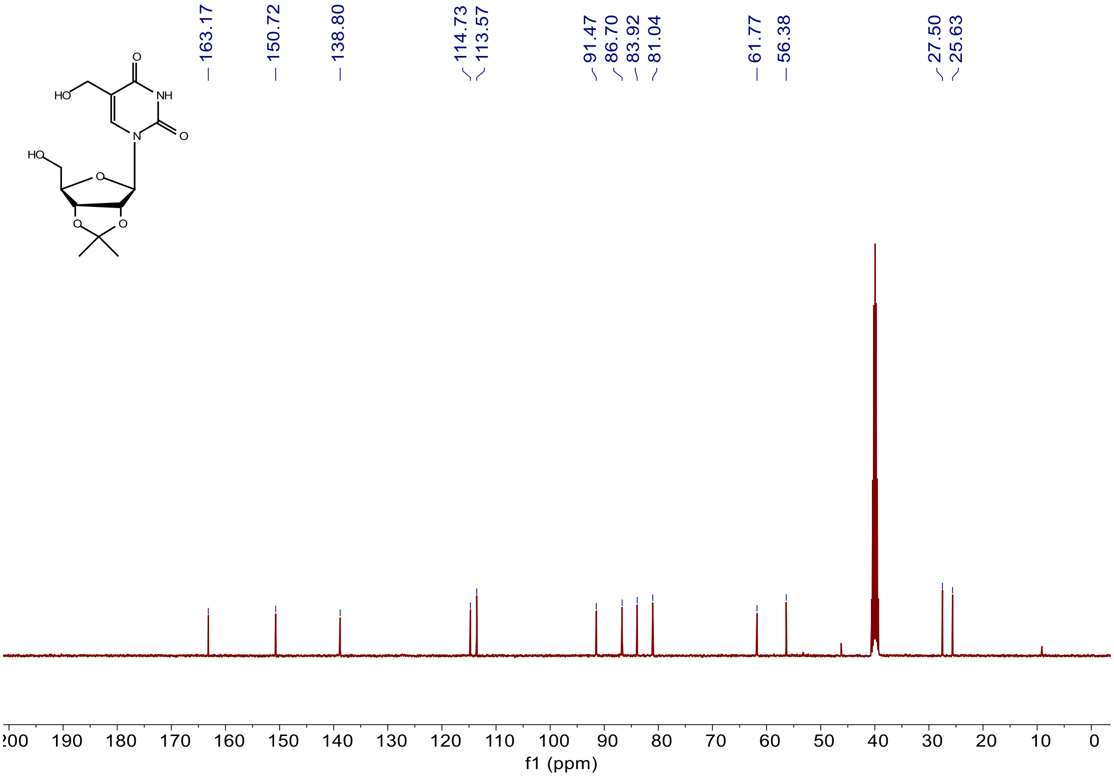

^{13}C NMR spectrum of compound **20** in DMSO-d_6

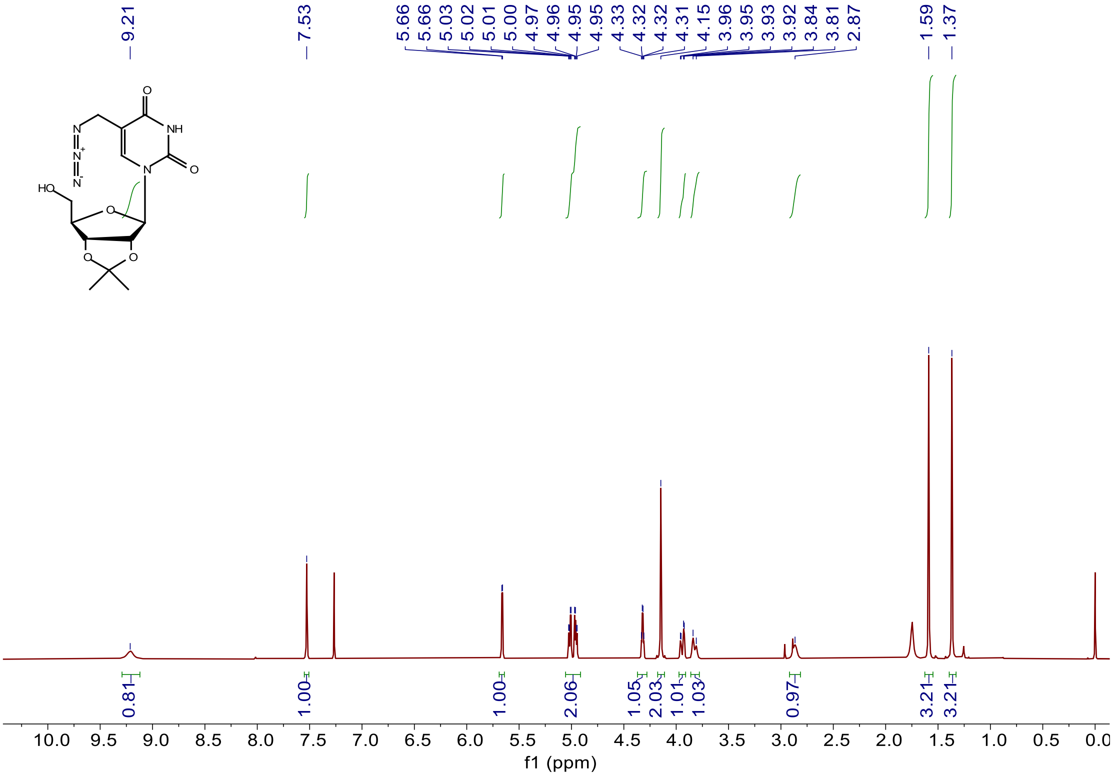

^{1}**H NMR** spectrum of compound **21** in DMSO-d_6

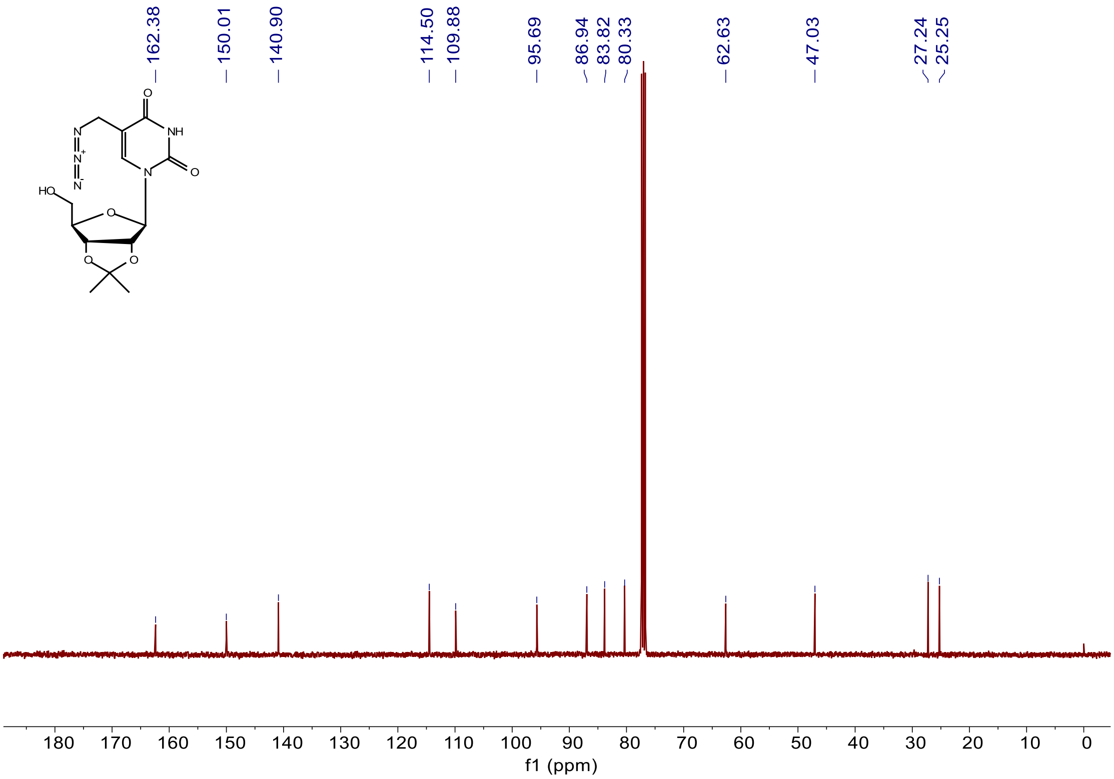

^{13}**C NMR** spectrum of compound **21** in DMSO-d_6

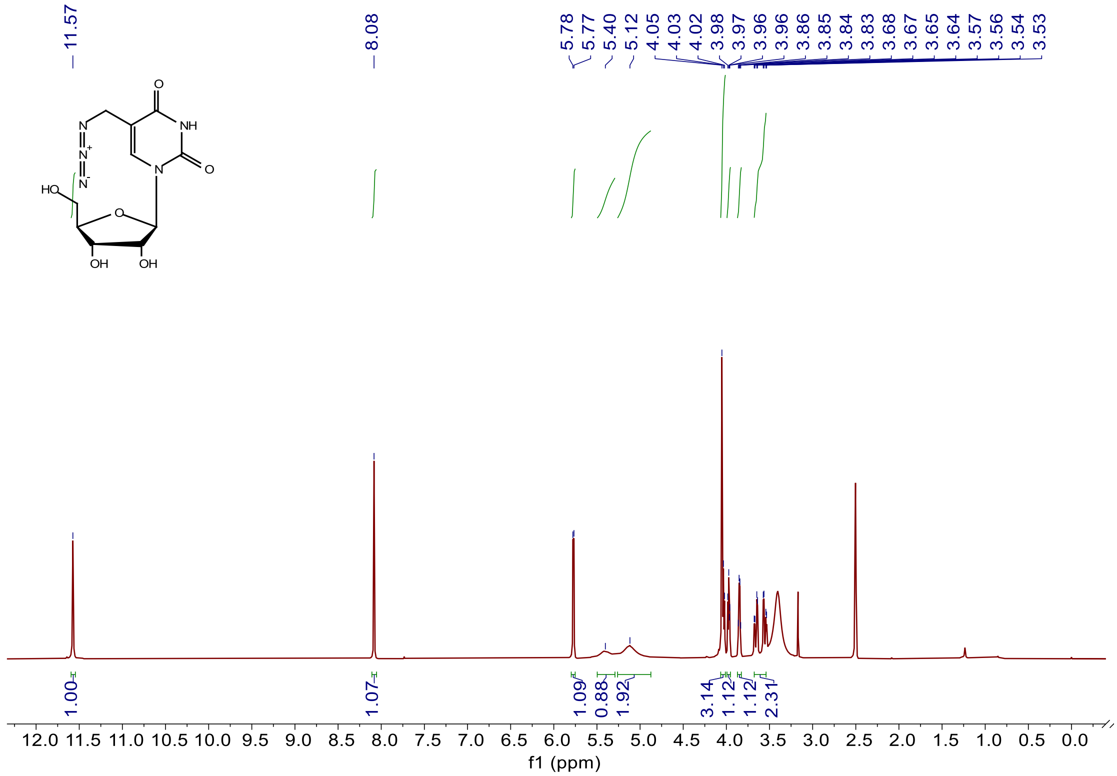

^{1}H NMR spectrum of compound **22** in DMSO-d_6

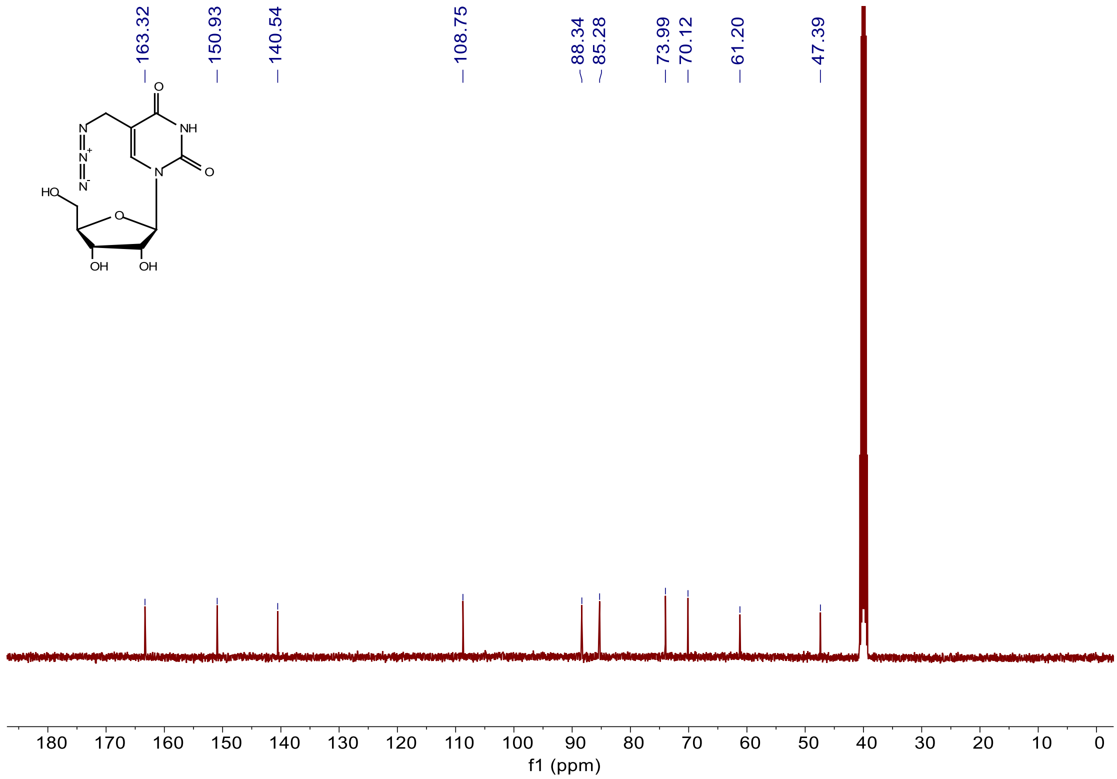

^{13}C NMR spectrum of compound **22** in DMSO-d_6

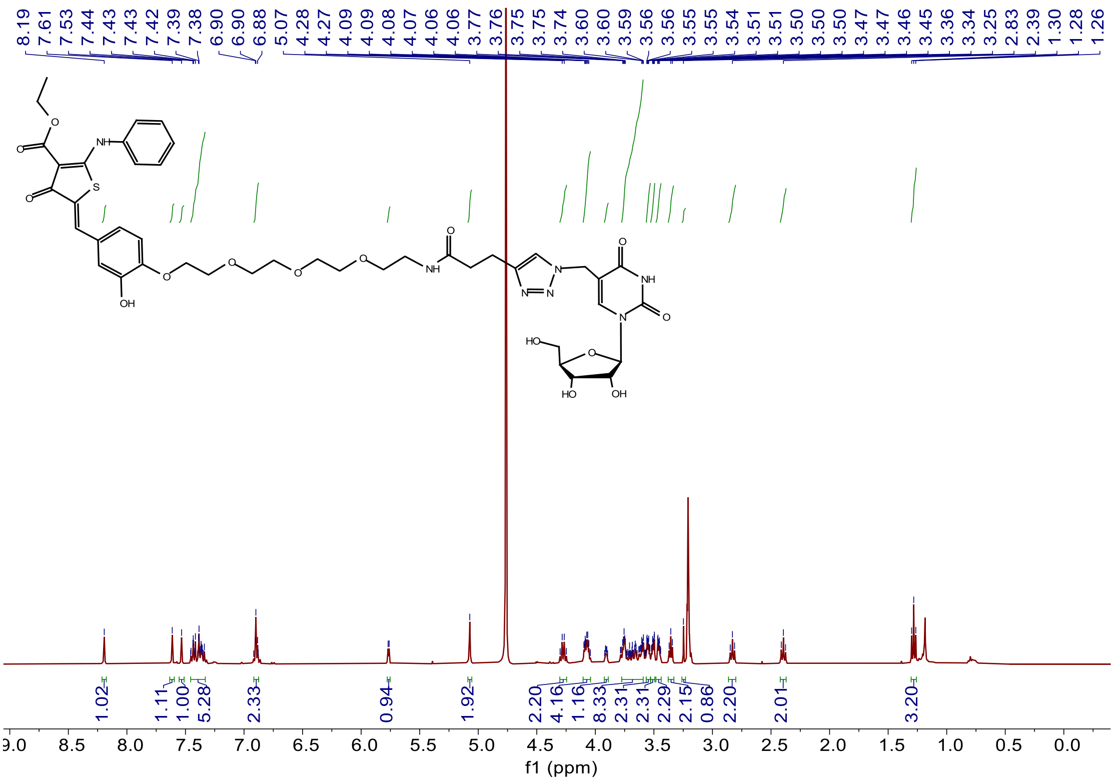

^{1}H NMR spectrum of **5-RIBOTAC-U (23)** in methanol-d_4

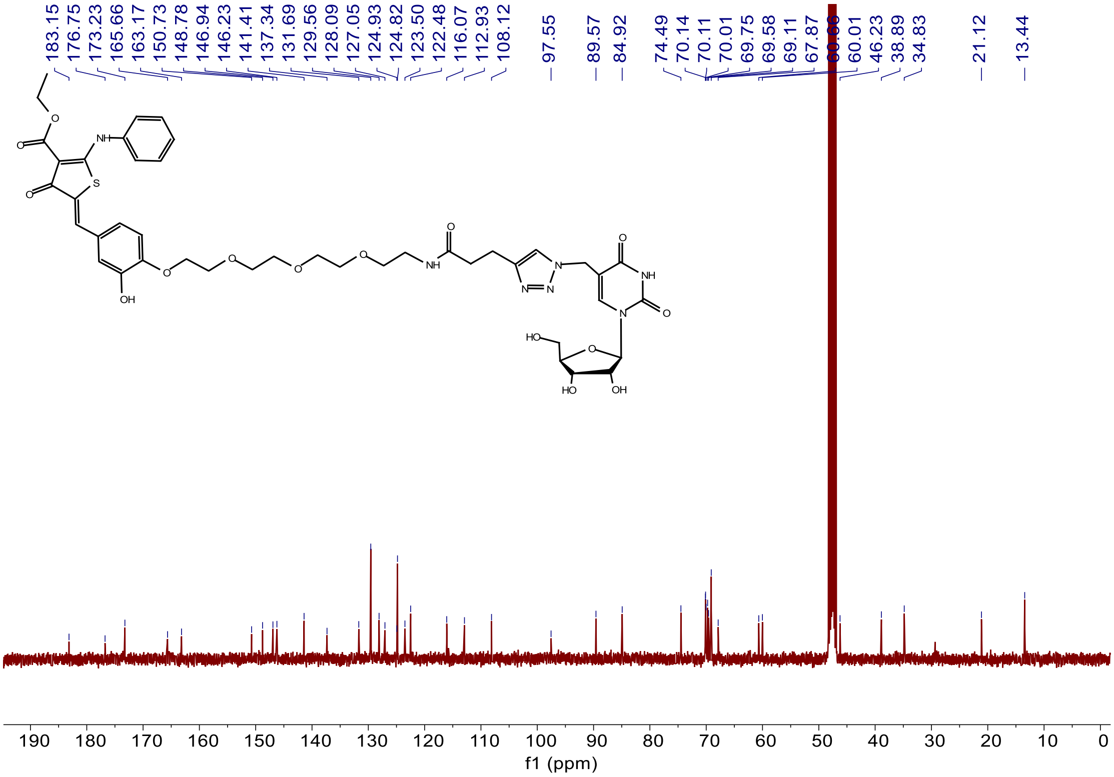

^{13}C NMR spectrum of **5-RIBOTAC-U (23)** in methanol-d_4

Data S2. HRMS Spectra

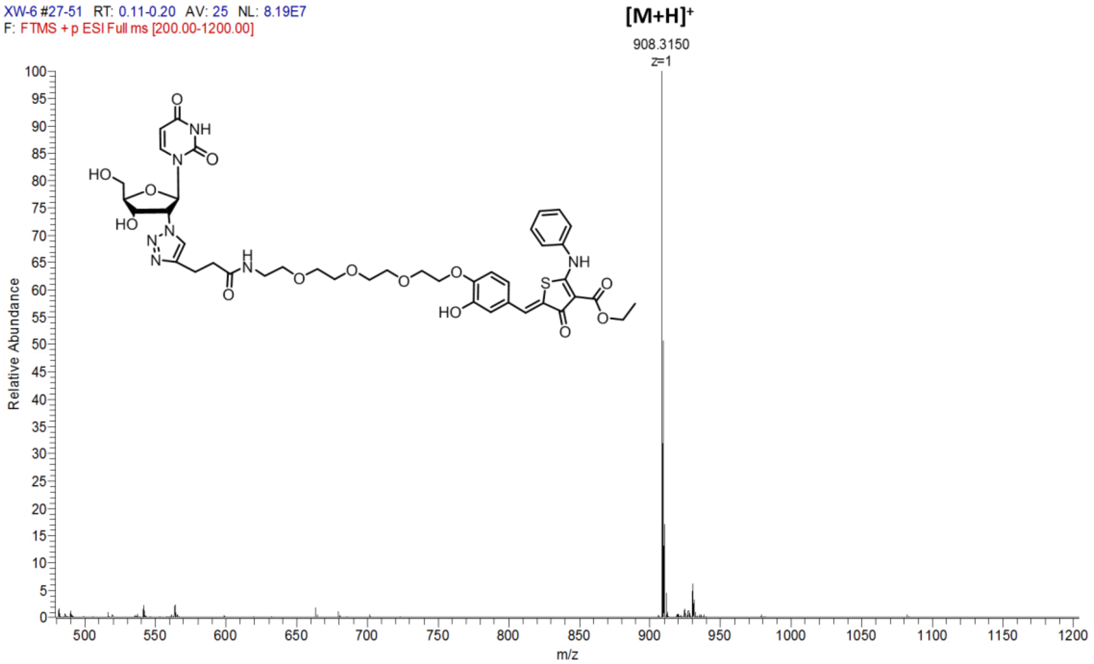

HRMS spectrum of 2'-RIBOTAC-U

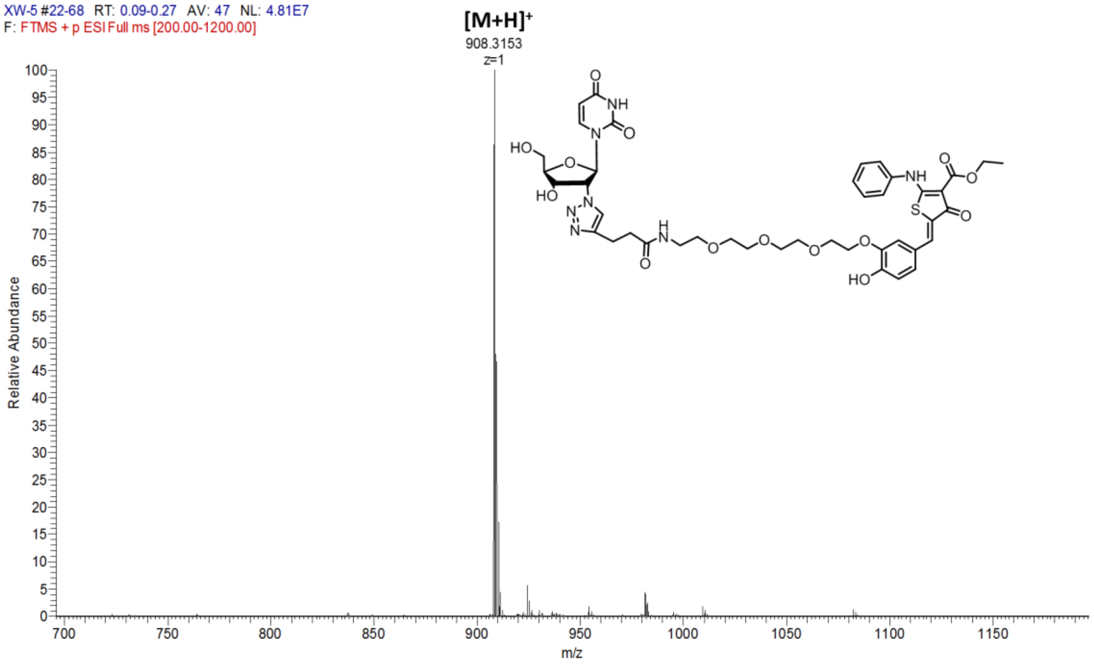

HRMS spectrum of 2'-mutRIBOTAC-U

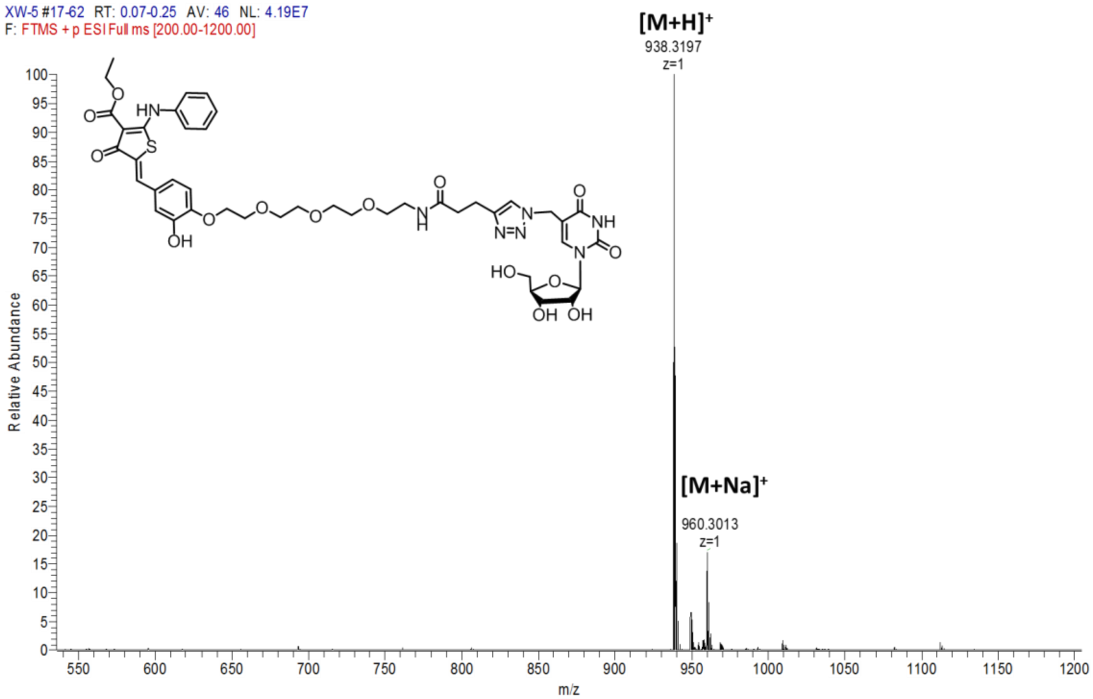

HRMS spectrum of 5-RIBOTAC-U

Supplementary Files:

Data S3 – Raw data for all qRT-PCR experiments

REFERENCES AND NOTES

1. M. Wang, R. Cao, L. Zhang, X. Yang, J. Liu, M. Xu, Z. Shi, Z. Hu, W. Zhong, G. Xiao, Remdesivir and chloroquine effectively inhibit the recently emerged novel coronavirus (2019-nCoV) in vitro. *Cell Res.* **30**, 269–271 (2020).

2. J. Liu, R. Cao, M. Xu, X. Wang, H. Zhang, H. Hu, Y. Li, Z. Hu, W. Zhong, M. Wang, Hydroxychloroquine, a less toxic derivative of chloroquine, is effective in inhibiting SARS-CoV-2 infection in vitro. *Cell Discov.* **6**, 16 (2020).

3. Y. Xie, W. Yin, Y. Zhang, W. Shang, Z. Wang, X. Luan, G. Tian, H. A. Aisa, Y. Xu, G. Xiao, J. Li, H. Jiang, S. Zhang, L. Zhang, H. E. Xu, J. Shen, Design and development of an oral remdesivir derivative VV116 against SARS-CoV-2. *Cell Res.* **31**, 1212–1214 (2021).

4. G. Li, R. Hilgenfeld, R. Whitley, E. De Clercq, Therapeutic strategies for COVID-19: Progress and lessons learned. *Nat. Rev. Drug Discov.* **22**, 449–475 (2023).

5. A. von Delft, M. D. Hall, A. D. Kwong, L. A. Purcell, K. S. Saikatendu, U. Schmitz, J. A. Tallarico, A. A. Lee, Accelerating antiviral drug discovery: Lessons from COVID-19. *Nat. Rev. Drug Discov.* **22**, 585–603 (2023).

6. C. Liu, Q. Zhou, Y. Li, L. V. Garner, S. P. Watkins, L. J. Carter, J. Smoot, A. C. Gregg, A. D. Daniels, S. Jervey, D. Albaiu, Research and development on therapeutic agents and vaccines for COVID-19 and related human coronavirus diseases. *ACS Cent. Sci.* **6**, 315–331 (2020).

7. J. Qiao, Y.-S. Li, R. Zeng, F.-L. Liu, R.-H. Luo, C. Huang, Y.-F. Wang, J. Zhang, B. Quan, C. Shen, X. Mao, X. Liu, W. Sun, W. Yang, X. Ni, K. Wang, L. Xu, Z.-L. Duan, Q.-C. Zou, H.-L. Zhang, W. Qu, Y.-H.-P. Long, M.-H. Li, R.-C. Yang, X. Liu, J. You, Y. Zhou, R. Yao, W.-P. Li, J.-M. Liu, P. Chen, Y. Liu, G.-F. Lin, X. Yang, J. Zou, L. Li, Y. Hu, G.-W. Lu, W.-M. Li, Y.-Q. Wei, Y.-T. Zheng, J. Lei, S. Yang, SARS-CoV-2 M^{pro} inhibitors with antiviral activity in a transgenic mouse model. *Science* **371**, 1374–1378 (2021).

8. W. Dai, B. Zhang, X.-M. Jiang, H. Su, J. Li, Y. Zhao, X. Xie, Z. Jin, J. Peng, F. Liu, C. Li, Y. Li, F. Bai, H. Wang, X. Cheng, X. Cen, S. Hu, X. Yang, J. Wang, X. Liu, G. Xiao, H. Jiang, Z.

Rao, L.-K. Zhang, Y. Xu, H. Yang, H. Liu, Structure-based design of antiviral drug candidates targeting the SARS-CoV-2 main protease. *Science* **368**, 1331–1335 (2020).

9. Z. Jin, X. Du, Y. Xu, Y. Deng, M. Liu, Y. Zhao, B. Zhang, X. Li, L. Zhang, C. Peng, Y. Duan, J. Yu, L. Wang, K. Yang, F. Liu, R. Jiang, X. Yang, T. You, X. Liu, X. Yang, F. Bai, H. Liu, X. Liu, L. W. Guddat, W. Xu, G. Xiao, C. Qin, Z. Shi, H. Jiang, Z. Rao, H. Yang, Structure of M^{pro} from SARS-CoV-2 and discovery of its inhibitors. *Nature* **582**, 289–293 (2020).

10. B. Hu, H. Guo, P. Zhou, Z. L. Shi, Characteristics of SARS-CoV-2 and COVID-19. *Nat. Rev. Microbiol.* **19**, 141–154 (2021).

11. W. T. Harvey, A. M. Carabelli, B. Jackson, R. K. Gupta, E. C. Thomson, E. M. Harrison, C. Ludden, R. Reeve, A. Rambaut; COVID-19 Genomics UK (COG-UK) Consortium, S. J. Peacock, D. L. Robertson, SARS-CoV-2 variants, spike mutations and immune escape. *Nat. Rev. Microbiol.* **19**, 409–424 (2021).

12. C. Chakraborty, M. Bhattacharya, A. R. Sharma, Emerging mutations in the SARS-CoV-2 variants and their role in antibody escape to small molecule-based therapeutic resistance. *Curr. Opin. Pharmacol.* **62**, 64–73 (2022).

13. D. C. Schultz, R. M. Johnson, K. Ayyanathan, J. Miller, K. Whig, B. Kamalia, M. Dittmar, S. Weston, H. L. Hammond, C. Dillen, J. Ardanuy, L. Taylor, J. S. Lee, M. Li, E. Lee, C. Shoffler, C. Petucci, S. Constant, M. Ferrer, C. A. Thaiss, M. B. Frieman, S. Cherry, Pyrimidine inhibitors synergize with nucleoside analogues to block SARS-CoV-2. *Nature* **604**, 134–140 (2022).

14. M. Seifert, S. C. Bera, P. Van Nies, R. N. Kirchdoerfer, A. Shannon, T.-T.-N. Le, X. Meng, H. Xia, J. M. Wood, L. D. Harris, F. S. Papini, J. J. Arnold, S. Almo, T. L. Grove, P.-Y. Shi, Y. Xiang, B. Canard, M. Depken, C. E. Cameron, D. Dulin, Inhibition of SARS-CoV-2 polymerase by nucleotide analogs from a single-molecule perspective. *eLife* **10**, e70968 (2021).

15. H.-J. Qian, Y. Wang, M.-Q. Zhang, Y.-C. Xie, Q.-Q. Wu, L.-Y. Liang, Y. Cao, H.-Q. Duan, G.-H. Tian, J. Ma, Z.-B. Zhang, N. Li, J.-Y. Jia, J. Zhang, H. A. Aisa, J.-S. Shen, C. Yu, H.-L. Jiang, W.-H. Zhang, Z. Wang, G.-Y. Liu, Safety, tolerability, and pharmacokinetics of VV116, an oral nucleoside analog against SARS-CoV-2, in Chinese healthy subjects. *Acta Pharmacol. Sin.* **43**, 3130–3138 (2022).

16. A. Schäfer, D. R. Martinez, J. J. Won, R. M. Meganck, F. R. Moreira, A. J. Brown, K. L. Gully, M. R. Zweigart, W. S. Conrad, S. R. May, S. Dong, R. Kalla, K. Chun, V. D. Pont, D. Babusis, J. Tang, E. Murakami, R. Subramanian, K. T. Barrett, B. J. Bleier, R. Bannister, J. Y. Feng, J. P. Bilello, T. Cihlar, R. L. Mackman, S. A. Montgomery, R. S. Baric, T. P. Sheahan, Therapeutic treatment with an oral prodrug of the remdesivir parental nucleoside is protective against SARS-CoV-2 pathogenesis in mice. *Sci. Transl. Med.* **14**, eabm3410 (2022).

17. A. Simonis, S. J. Theobald, G. Fätkenheuer, J. Rybniker, J. J. Malin, A comparative analysis of remdesivir and other repurposed antivirals against SARS-CoV-2. *EMBO Mol. Med.* **13**, e13105 (2021).

18. K. Zandi, F. Amblard, K. Musall, J. Downs-Bowen, R. Kleinbard, A. Oo, D. Cao, B. Liang, O. O. Russell, T. McBrayer, L. Bassit, B. Kim, R. F. Schinazi, Repurposing nucleoside analogs for human coronaviruses. *Antimicrob. Agents Chemother.* **65**, e01652-20 (2020).

19. J. Sourimant, C. M. Lieber, M. Aggarwal, R. M. Cox, J. D. Wolf, J.-J. Yoon, M. Toots, C. Ye, Z. Sticher, A. A. Kolykhalov, L. Martinez-Sobrido, G. R. Bluemling, M. G. Natchus, G. R. Painter, R. K. Plemper, 4′-Fluorouridine is an oral antiviral that blocks respiratory syncytial virus and SARS-CoV-2 replication. *Science* **375**, 161–167 (2022).

20. M. Chien, T. K. Anderson, S. Jockusch, C. Tao, X. Li, S. Kumar, J. J. Russo, R. N. Kirchdoerfer, J. Ju, Nucleotide analogues as inhibitors of SARS-CoV-2 polymerase, a key drug target for COVID-19. *J. Proteome Res.* **19**, 4690–4697 (2020).

21. L. Zhao, W. Zhong, Mechanism of action of favipiravir against SARS-CoV-2: Mutagenesis or chain termination? *Innovation* **2**, 100165 (2021).

22. F. Kabinger, C. Stiller, J. Schmitzová, C. Dienemann, G. Kokic, H. S. Hillen, C. Höbartner, P. Cramer, Mechanism of molnupiravir-induced SARS-CoV-2 mutagenesis. *Nat. Struct. Mol. Biol.* **28**, 740–746 (2021).

23. A. Jayk Bernal, M. M. Gomes da Silva, D. B. Musungaie, E. Kovalchuk, A. Gonzalez, V. Delos Reyes, A. Martín-Quirós, Y. Caraco, A. Williams-Diaz, M. L. Brown, J. Du, A. Pedley, C. Assaid, J. Strizki, J. A. Grobler, H. H. Shamsuddin, R. Tipping, H. Wan, A. Paschke, J. R. Butterton, M. G. Johnson, C. De Anda; MOVe-OUT Study Group, Molnupiravir for oral treatment of Covid-19 in nonhospitalized patients. *N. Engl. J. Med.* **386**, 509–520 (2022).

24. W. A. Fischer II, J. J. Eron Jr., W. Holman, M. S. Cohen, L. Fang, L. J. Szewczyk, T. P. Sheahan, R. Baric, K. R. Mollan, C. R. Wolfe, E. R. Duke, M. M. Azizad, K. Borroto-Esoda, D. A. Wohl, R. W. Coombs, A. J. Loftis, P. Alabanza, F. Lipansky, W. P. Painter, A phase 2a clinical trial of molnupiravir in patients with COVID-19 shows accelerated SARS-CoV-2 RNA clearance and elimination of infectious virus. *Sci. Transl. Med.* **14**, eabl7430 (2021).

25. F. Pourkarim, S. Pourtaghi-Anvarian, H. Rezaee, Molnupiravir: A new candidate for COVID-19 treatment. *Pharmacol. Res. Perspect.* **10**, e00909 (2022).

26. J. P. K. Bravo, T. L. Dangerfield, D. W. Taylor, K. A. Johnson, Remdesivir is a delayed translocation inhibitor of SARS-CoV-2 replication. *Mol. Cell* **81**, 1548–1552.e4 (2021).

27. E. P. Tchesnokov, C. J. Gordon, E. Woolner, D. Kocinkova, J. K. Perry, J. Y. Feng, D. P. Porter, M. Götte, Template-dependent inhibition of coronavirus RNA-dependent RNA polymerase by remdesivir reveals a second mechanism of action. *J. Biol. Chem.* **295**, 16156–16165 (2020).

28. C. J. Gordon, E. P. Tchesnokov, R. F. Schinazi, M. Götte, Molnupiravir promotes SARS-CoV-2 mutagenesis via the RNA template. *J. Biol. Chem.* **297**, 100770 (2021).

29. R. Swanstrom, R. F. Schinazi, Lethal mutagenesis as an antiviral strategy. *Science* **375**, 497–498 (2022).

30. G. Santi Laurini, N. Montanaro, D. Motola, Safety profile of molnupiravir in the treatment of COVID-19: A descriptive study based on FAERS data. *J. Clin. Med.* **12**, 34 (2022).

31. A. M. Yu, Y. H. Choi, M.-J. Tu, RNA drugs and RNA targets for small molecules: Principles, progress, and challenges. *Pharmacol. Rev.* **72**, 862–898 (2020).

32. J. L. Childs-Disney, X. Yang, Q. M. R. Gibaut, Y. Tong, R. T. Batey, M. D. Disney, Targeting RNA structures with small molecules. *Nat. Rev. Drug Discov.* **21**, 736–762 (2022).

33. A. Donlic, E. G. Swanson, L.-Y. Chiu, S. L. Wicks, A. U. Juru, Z. Cai, K. Kassam, C. Laudeman, B. G. Sanaba, A. Sugarman, E. Han, B. S. Tolbert, A. E. Hargrove, R-BIND 2.0: An updated database of bioactive RNA-targeting small molecules and associated RNA secondary structures. *ACS Chem. Biol.* **17**, 1556–1566 (2022).

34. C. S. Thakur, B. K. Jha, B. Dong, J. Das Gupta, K. M. Silverman, H. Mao, H. Sawai, A. O. Nakamura, A. K. Banerjee, A. Gudkov, R. H. Silverman, Small-molecule activators of RNase L with broad-spectrum antiviral activity. *Proc. Natl. Acad. Sci. U.S.A.* **104**, 9585–9590 (2007).

35. S. Mikutis, M. Rebelo, E. Yankova, M. Gu, C. Tang, A. R. Coelho, M. Yang, M. E. Hazemi, M. Pires de Miranda, M. Eleftheriou, M. Robertson, G. S. Vassiliou, D. J. Adams, J. P. Simas, F. Corzana, J. S. Schneekloth Jr., K. Tzelepis, G. J. L. Bernardes, Proximity-induced nucleic acid degrader (PINAD) approach to targeted RNA degradation using small molecules. *ACS Cent. Sci.* **9**, 892–904 (2023).

36. X. Su, W. Ma, D. Feng, B. Cheng, Q. Wang, Z. Guo, D. Zhou, X. Tang, Efficient inhibition of SARS-CoV-2 using chimeric antisense oligonucleotides through RNase L activation. *Angew. Chem. Int. Ed.* **60**, 21662–21667 (2021).

37. S. Haj-Yahia, A. Nandi, R. I. Benhamou, Targeted degradation of structured RNAs via ribonuclease-targeting chimeras (RiboTacs). *Expert Opin. Drug Discovery* **18**, 929–942 (2023).

38. Y. Tong, Y. Lee, X. Liu, J. L. Childs-Disney, B. M. Suresh, R. I. Benhamou, C. Yang, W. Li, M. G. Costales, H. S. Haniff, S. Sievers, D. Abegg, T. Wegner, T. O. Paulisch, E. Lekah, M.

Grefe, G. Crynen, M. Van Meter, T. Wang, Q. M. R. Gibaut, J. L. Cleveland, A. Adibekian, F. Glorius, H. Waldmann, M. D. Disney, Programming inactive RNA-binding small molecules into bioactive degraders. *Nature* **618**, 169–179 (2023).

39. H. S. Haniff, Y. Tong, X. Liu, J. L. Chen, B. M. Suresh, R. J. Andrews, J. M. Peterson, C. A. O'Leary, R. I. Benhamou, W. N. Moss, M. D. Disney, Targeting the SARS-CoV-2 RNA genome with small molecule binders and ribonuclease targeting chimera (RIBOTAC) degraders. *ACS Cent. Sci.* **6**, 1713–1721 (2020).

40. M. G. Costales, Y. Matsumoto, S. P. Velagapudi, M. D. Disney, Small molecule targeted recruitment of a nuclease to RNA. *J. Am. Chem. Soc.* **140**, 6741–6744 (2018).

41. P. Zhang, X. Liu, D. Abegg, T. Tanaka, Y. Tong, R. I. Benhamou, J. Baisden, G. Crynen, S. M. Meyer, M. D. Cameron, A. K. Chatterjee, A. Adibekian, J. L. Childs-Disney, M. D. Disney, Reprogramming of protein-targeted small-molecule medicines to RNA by ribonuclease recruitment. *J. Am. Chem. Soc.* **143**, 13044–13055 (2021).

42. A. Eccleston, A strategy for small-molecule RNA degraders. *Nat. Rev. Drug Discov.* **22**, 621 (2023).

43. K. D. Warner, C. E. Hajdin, K. M. Weeks, Principles for targeting RNA with drug-like small molecules. *Nat. Rev. Drug Discov.* **17**, 547–558 (2018).

44. L. Guan, M. D. Disney, Recent advances in developing small molecules targeting RNA. *ACS Chem. Biol.* **7**, 73–86 (2012).

45. T. Tran, M. D. Disney, Identifying the preferred RNA motifs and chemotypes that interact by probing millions of combinations. *Nat. Commun.* **3**, 1125 (2012).

46. D. Wang, Y. Zhang, R. E. Kleiner, Cell- and polymerase-selective metabolic labeling of cellular RNA with 2′-azidocytidine. *J. Am. Chem. Soc.* **142**, 14417–14421 (2020).

47. S. Beasley, A. Vandewalle, M. Singha, K. Nguyen, W. England, E. Tarapore, N. Dai, I. R. Corrêa Jr, S. X. Atwood, R. C. Spitale, Exploiting endogenous enzymes for cancer-cell selective metabolic labeling of RNA in vivo. *J. Am. Chem. Soc.* **144**, 7085–7088 (2022).

48. D. Wang, A. Shalamberidze, A. E. Arguello, B. W. Purse, R. E. Kleiner, Live-cell RNA imaging with metabolically incorporated fluorescent nucleosides. *J. Am. Chem. Soc.* **144**, 14647–14656 (2022).

49. Y. Zhang, R. E. Kleiner, A metabolic engineering approach to incorporate modified pyrimidine nucleosides into cellular RNA. *J. Am. Chem. Soc.* **141**, 3347–3351 (2019).

50. M. G. Costales, H. Aikawa, Y. Li, J. L. Childs-Disney, D. Abegg, D. G. Hoch, S. Pradeep Velagapudi, Y. Nakai, T. Khan, K. W. Wang, I. Yildirim, A. Adibekian, E. T. Wang, M. D. Disney, Small-molecule targeted recruitment of a nuclease to cleave an oncogenic RNA in a mouse model of metastatic cancer. *Proc. Natl. Acad. Sci. U.S.A.* **117**, 2406–2411 (2020).

51. Y.-Q. Min, M. Huang, K. Feng, Y. Jia, X. Sun, Y.-J. Ning, A new cellular interactome of SARS-CoV-2 nucleocapsid protein and its biological implications. *Mol. Cell. Proteomics* **22**, 100579 (2023).

52. M. Imai, K. Iwatsuki-Horimoto, M. Hatta, S. Loeber, P. J. Halfmann, N. Nakajima, T. Watanabe, M. Ujie, K. Takahashi, M. Ito, S. Yamada, S. Fan, S. Chiba, M. Kuroda, L. Guan, K. Takada, T. Armbrust, A. Balogh, Y. Furusawa, M. Okuda, H. Ueki, A. Yasuhara, Y. Sakai-Tagawa, T. J. S. Lopes, M. Kiso, S. Yamayoshi, N. Kinoshita, N. Ohmagari, S.-I. Hattori, M. Takeda, H. Mitsuya, F. Krammer, T. Suzuki, Y. Kawaoka, Syrian hamsters as a small animal model for SARS-CoV-2 infection and countermeasure development. *Proc. Natl. Acad. Sci. U.S.A.* **117**, 16587–16595 (2020).

53. A. Best Rocha, E. Stroberg, L. M. Barton, E. J. Duval, S. Mukhopadhyay, N. Yarid, T. Caza, J. D. Wilson, D. J. Kenan, M. Kuperman, S. G. Sharma, C. P. Larsen, Detection of SARS-CoV-2 in formalin-fixed paraffin-embedded tissue sections using commercially available reagents. *Lab. Invest.* **100**, 1485–1489 (2020).

54. A. Wahl, L. E. Gralinski, C. E. Johnson, W. Yao, M. Kovarova, K. H. Dinnon III, H. Liu, V. J. Madden, H. M. Krzystek, C. De, K. K. White, K. Gully, A. Schäfer, T. Zaman, S. R. Leist, P. O. Grant, G. R. Bluemling, A. A. Kolykhalov, M. G. Natchus, F. B. Askin, G. Painter, E. P. Browne, C. D. Jones, R. J. Pickles, R. S. Baric, J. V. Garcia, SARS-CoV-2 infection is effectively treated and prevented by EIDD-2801. *Nature* **591**, 451–457 (2021).

55. B. F. Malone, J. K. Perry, P. D. B. Olinares, H. W. Lee, J. Chen, T. C. Appleby, J. Y. Feng, J. P. Bilello, H. Ng, J. Sotiris, M. Ebrahim, E. Y. D. Chua, J. H. Mendez, E. T. Eng, R. Landick, M. Götte, B. T. Chait, E. A. Campbell, S. A. Darst, Structural basis for substrate selection by the SARS-CoV-2 replicase. *Nature* **614**, 781–787 (2023).

56. S. Masyeni, M. Iqhrammullah, A. Frediansyah, F. Nainu, T. Tallei, T. B. Emran, Y. Ophinni, K. Dhama, H. Harapan, Molnupiravir: A lethal mutagenic drug against rapidly mutating severe acute respiratory syndrome coronavirus 2—A narrative review. *J. Med. Virol.* **94**, 3006–3016 (2022).

57. S. K. Dey, S. R. Jaffrey, RIBOTACs: Small molecules target RNA for degradation. *Cell Chem. Biol.* **26**, 1047–1049 (2019).

58. H. L. Lightfoot, G. F. Smith, Targeting RNA with small molecules-A safety perspective. *Br. J. Pharmacol.*, 10.1111/bph.16027 (2023).

59. K. Feng, Y. Q. Min, X. Sun, F. Deng, P. Li, H. Wang, Y. J. Ning, Interactome profiling reveals interaction of SARS-CoV-2 NSP13 with host factor STAT1 to suppress interferon signaling. *J. Mol. Cell Biol.* **13**, 760–762 (2021).

60. F. d'Orchymont, J. P. Holland, Supramolecular Rotaxane-Based Multi-Modal Probes for Cancer Biomarker Imaging. *Angew. Chem. Int. Ed.* **61**, e202204072 (2022).

61. Y. Mehellou, R. Valente, H. Mottram, E. Walsby, K. I. Mills, J. Balzarini, C. McGuigan, Phosphoramidates of 2′-β-d-arabinouridine (AraU) as phosphate prodrugs; design, synthesis, in vitro activity and metabolism. *Bioorg. Med. Chem.* **18**, 2439–2446 (2010).

62. D. Mathur, N. Rana, C. E. Olsen, V. S. Parmar, A. K. Prasad, Cu(I)-catalyzed efficient synthesis of 2′-triazolo-nucleoside conjugates. *J. Heterocycl. Chem.* **52**, 701–710 (2015).

63. M. Fujihashi, T. Ishida, S. Kuroda, L. P. Kotra, E. F. Pai, K. Miki, Substrate distortion contributes to the catalysis of orotidine 5′-monophosphate decarboxylase. *J. Am. Chem. Soc.* **135**, 17432–17443 (2013).

64. A.-H. Adel, E. S. El Ashry, Efficient synthesis of 5-hydroxymethyl pyrimidines and their nucleosides using microwave irradiation. *Synlett*, 2043–2044 (2002).

65. G. Leszczynska, P. Leonczak, K. Wozniak, A. Malkiewicz, Chemical synthesis of the 5-taurinomethyl (−2-thio) uridine modified anticodon arm of the human mitochondrial tRNALeu (UUR) and tRNALys. *RNA* **20**, 938–947 (2014).

66. P. Zhou, X.-L. Yang, X.-G. Wang, B. Hu, L. Zhang, W. Zhang, H.-R. Si, Y. Zhu, B. Li, C.-L. Huang, H.-D. Chen, J. Chen, Y. Luo, H. Guo, R.-D. Jiang, M.-Q. Liu, Y. Chen, X.-R. Shen, X. Wang, X.-S. Zheng, K. Zhao, Q.-J. Chen, F. Deng, L.-L. Liu, B. Yan, F.-X. Zhan, Y.-Y. Wang, G.-F. Xiao, Z.-L. Shi, A pneumonia outbreak associated with a new coronavirus of probable bat origin. *Nature* **579**, 270–273 (2020).
